# Chiral Aminoalcohols and Squaric Acid Amides as Ligands for Asymmetric Borane Reduction of Ketones: Insight to In Situ Formed Catalytic System by DOSY and Multinuclear NMR Experiments

**DOI:** 10.3390/molecules26226865

**Published:** 2021-11-14

**Authors:** Yana Nikolova, Georgi M. Dobrikov, Zhanina Petkova, Pavletta Shestakova

**Affiliations:** Institute of Organic Chemistry with Centre of Phytochemistry, Bulgarian Academy of Sciences, Bl. 9, Acad. G. Bonchev Str., 1113 Sofia, Bulgaria; Georgi.Dobrikov@orgchm.bas.bg (G.M.D.); Zhanina.Petkova@orgchm.bas.bg (Z.P.)

**Keywords:** asymmetric reduction, borane complexes, chiral aminoalcohols, DOSY NMR, squaramides

## Abstract

A series of squaric acid amides (synthesized in 66–99% isolated yields) and a set of chiral aminoalcohols were comparatively studied as ligands in a model reaction of reduction of α-chloroacetophenone with BH_3_•SMe_2_. In all cases, the aminoalcohols demonstrated better efficiency (up to 94% *ee*), while only poor asymmetric induction was achieved with the corresponding squaramides. A mechanistic insight on the in situ formation and stability at room temperature of intermediates generated from ligands and borane as possible precursors of the oxazaborolidine-based catalytic system has been obtained by ^1^H DOSY and multinuclear 1D and 2D (^1^H, ^10/11^B, ^13^C, ^15^N) NMR spectroscopy of equimolar mixtures of borane and selected ligands. These results contribute to better understanding the complexity of the processes occurring in the reaction mixture prior to the possible oxazaborolidine formation, which play a crucial role on the degree of enantioselectivity achieved in the borane reduction of α-chloroacetophenone.

## 1. Introduction

Asymmetric reduction of ketones still attracts significant interest since it is one of the most efficient ways of generating enantiomerically pure secondary alcohols as important intermediates or chiral building blocks for the synthesis of biologically relevant structures [1,2,3,4,5,6,7,8]. Among the various well-known approaches, borane reduction has been demonstrated to allow fine-tuning for attaining a higher degree of selectivity. Remarkable success in this reaction has been achieved using chiral oxazaborolidines prepared from (semi)synthetic aminoalcohols derived from natural amino acids, terpenoids [9,10,11], etc. Commonly, more effective among them are the bicyclic oxazaborolidines incorporating rigid or sterically constrained aminoalcohol moieties [12]. To date, the established synthetic procedures for the preparation of oxazaborolidines usually require excess of boron reagent (BH_3_•THF or BH_3_•SMe_2_), prolonged reaction times, elevated temperatures and dry conditions for the isolation step [10,13,14,15,16,17]. More efficient are protocols for synthesis of alkyl- and aryl-B-substituted oxazaborolidines by refluxing toluene solutions of the corresponding aminoalcohol and boronic acids or boroxines [12,18]. The synthetic procedure requiring the shortest reaction time makes use of bis(trifluoroethyl) boronates as a boron source [19]. To make the catalytic reaction less cumbersome, in another approach, the targeted oxazaborolidine catalytic system is prepared in situ. In this case, however, an experimental proof for the formation of catalytic species as well as their actual structures is often missing.

Interesting results have been reported in which squaric acid is used as a backbone to bind chiral aminoalcohol fragments [20,21,22,23]. The squaramides obtained have been used as ligands in borane reduction of activated ketones, providing high enantioselectivity (up to 99% *ee*). Based on these results, it can be concluded that the rigid and flat squaric acid skeleton in combination with chiral aminoalcohol fragments provides a structurally well-defined chiral environment for the asymmetric reaction. For explanation of the extraordinarily high asymmetric induction, a transition state model involving the squaramide ligand in the formation of the catalyst complex has been proposed [21,22,23]. Consequently, it is conceivable that novel structures obtained through the combination of squaric acid core as a backbone and suitable chiral amine and/or aminoalcohol moieties might have a positive influence on the borane reductions. The ease of substitution of squaric acid esters with fragments containing chiral amine or aminoalcohol moieties has enabled the synthesis of diverse chiral squaramide derivatives with various applications in asymmetric catalysis, mainly as organocatalysts [24,25,26,27,28,29,30,31]. Squaric acid derivatives are able to form complexes with first- and second-row transition metals [32,33,34]. The synthesis and structural elucidation of such complexes were thoroughly described. However, there are no literature data concerning the formation of complexes of squaric acid derivatives with boranes.

Herein, we report the synthesis of a series of squaramides incorporating structurally diverse aminoalcohol fragments and their application as chiral ligands in borane reduction (BH_3_•SMe_2_) of α-chloroacetophenone. Further, it is important to compare the obtained efficiency of the squaramides-catalyzed reductions with those of the corresponding free aminoalcohols, thus evaluating the contribution of the squaric acid fragment to the stereochemical outcome of the reaction. Particular attention is directed to study intermediates formed by the reaction between BH_3_•SMe_2_ and the chiral ligands (squaramides or the corresponding free aminoalcohols) applying ^1^H diffusion ordered NMR spectroscopy (DOSY) in combination with multinuclear (^1^H, ^10/11^B, ^13^C, ^15^N) 1D and 2D NMR experiments. DOSY [35] has proven to be a valuable tool for the investigation of molecular interactions and aggregation phenomena, including complex mixtures [36,37,38,39,40,41], supramolecular aggregates [42,43,44,45] and complexes [46,47,48,49], and to the best of our knowledge, has not been previously used to identify in situ generated intermediate species involved in oxazaborolidine formation in borane reduction of ketones. The technique has a well-established methodology, and during recent years, it has been successfully applied for the investigation of reaction intermediates in different reactions [50,51,52,53,54,55,56].

## 2. Results and Discussion

### 2.1. Synthesis

**Scheme 1:**
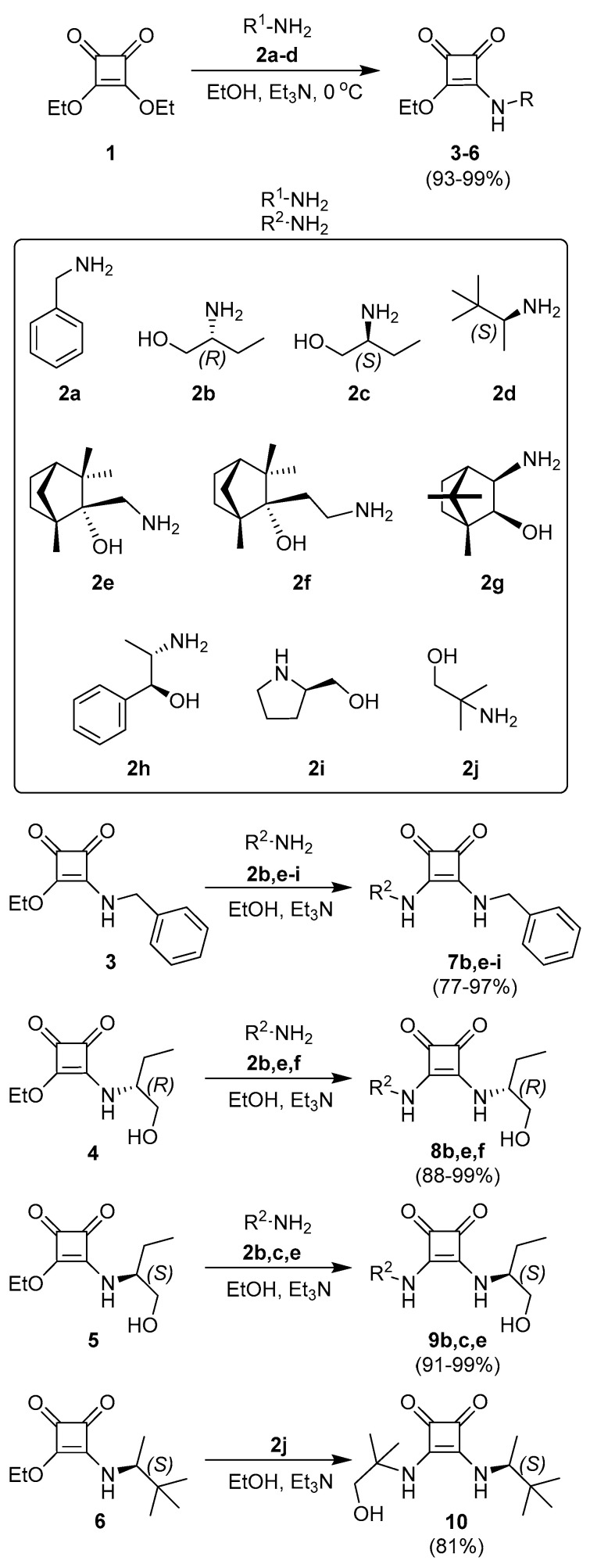
Synthesis of squaramides **3**–**10** by using chiral and nonchiral amines or aminoalcohols.

Two structural types of squaric acid amides, namely mono- and di-substituted derivatives, were synthesized. The monoamides **3**–**6** were prepared in high yields (93–99%) from **1** and amines **2a**–**d** with the purpose to use them as starting compounds for the synthesis of the disubstituted derivatives (Scheme 1). These starting compounds were designed to contain non-chiral and chiral amine moieties (**3** and **6**, respectively) and 2-aminobutanol fragment in the form of both enantiomers (**4** and **5**). For the synthesis of the disubstituted squaramides **7**–**10** (77–99% yields), the set of amines and aminoalcohols (**2a**–**j**) presented in Scheme 1 was used.

Compounds **2a**–**d** and **2h**–**j** are commercially available and were used directly as purchased. Chiral aminoalcohols **2e** [57], **2f** [57] and **2g** [58] were synthesized according to published procedures. Indeed, starting from enantiomerically pure natural compounds with known configuration, the stereochemistry of the expected products could be readily determined. This was further confirmed by comparing the properties (NMR, specific rotation) of the compounds with the reported data. Next, the preparation of diamides **7**–**10** was attempted. These compounds were designed to contain at least one chiral aminoalcohol or amine fragment.

**Scheme 2:**
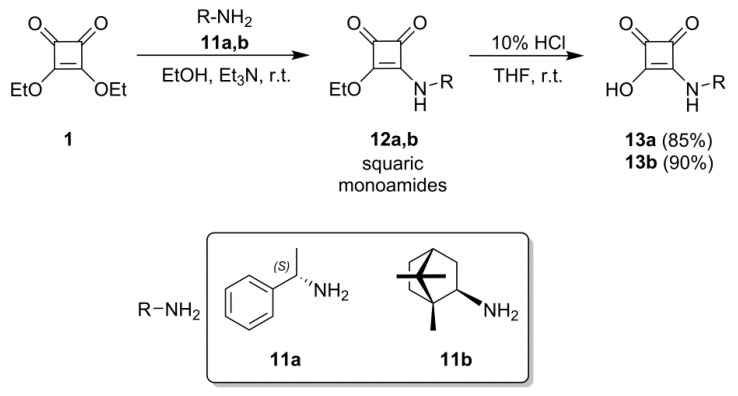
Synthesis of chiral squaramides **13a**,**b** bearing a hydroxyl group at the squaric core.

In a further reaction sequence, the squaric monoamides **13a,b** were synthesized by using amines **11a,b** (Scheme 2). Compounds **13a,b** were designed to incorporate hydroxy group directly attached to the squaric core.

**Scheme 3:**
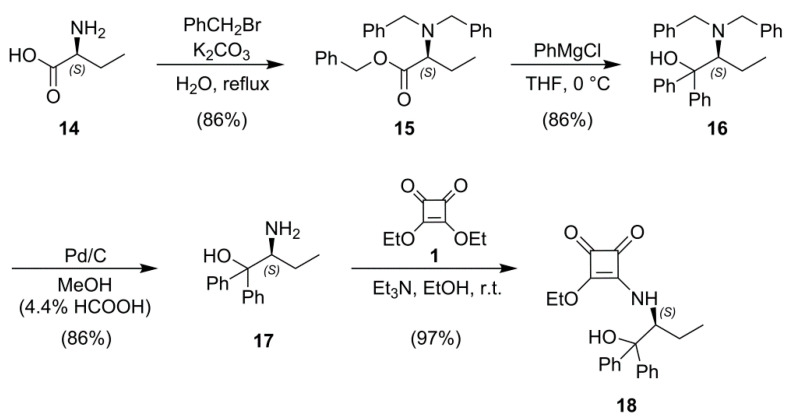
Synthesis of chiral diphenyl-substituted 2-aminobutanol analogue **17** and the corresponding squaric acid amide **18**.

Next, it was interesting to obtain aminoalcohol **17**, which is analogous to 2-aminobutanol (**2c**) and contains sterically demanding groups next to the hydroxy group (Scheme 3). Amino acid **14** was the suitable starting compound and it was easily transformed into benzyl ester **15**, simultaneously protecting the amine nitrogen with benzyl groups [59]. The subsequent addition of PhMgCl afforded **16** in high yield, which was finally transformed into the aminoalcohol **17**. Then, **17** reacts with squaric acid to form the corresponding monoamide **18** in high yield.

The synthesized squaric acid derivatives and the corresponding free aminoalcohols applied for their preparation were used as chiral ligands (L) in the borane reduction (BH_3_•SMe_2_) of α-chloroacetophenone (**19**) as a model reaction system (Table 1). The catalyst formation occurred in situ by mixing the corresponding ligand (0.1 equiv.) with BH_3_•SMe_2_ (1.2 equiv.) at room temperature in THF (stirring for 2 h) and then heating the mixture for 1 h at 50 °C prior to the addition of the ketone. The reduction was high yielding within very short reaction times (20–30 min). Generally, the enantioselectivities obtained using the squaric acid derivatives were moderate. Only in the case of ligands **4**, **8f** and **7g** did the enantioselectivity exceed 50%. In comparison, the enantioselectivities achieved with the corresponding free aminoalcohols were in all cases significantly higher (Table 1, entries 19–27). Excellent results have been obtained with both enantiomers of 2-aminobutanol and it is surprising that they have not yet been applied as ligands in borane reductions. Even better enantioselectivity (94% *ee*, determined by GC analysis with a chiral column; configurations were assigned by comparison of the sign of the specific rotation with that reported in the literature) was achieved with the newly synthesized aminoalcohol **17**, leading to the conclusion that the steric hindrance near the hydroxy group is important for improving the asymmetric induction. Therefore, considering the squaramides applied as ligands, it seems that only the chiral aminoalcohol fragment is responsible for the level of enantioinduction achieved in the asymmetric reaction.

The possible influence of the squaric acid core was studied with the synthesis and application of ligands **13a** and **13b**. These two compounds are formally similar to aminoalcohols in which the hydroxy group is directly attached to the squaric skeleton. In both cases, no enantioselectivity was observed (Table 1, entries 16 and 17). Likewise, in the case of ligand **10**, there is no asymmetric induction, although it formally possesses the attributes of an aminoalcohol, free hydroxy group and N-atom-bearing chiral fragment (Table 1, entry 15).

The significant role of the chiral aminoalcohol fragment incorporated in the squaramides is best illustrated in compound **9b**. In this case, there is a full compensation of the two enantiomeric 2-aminobutanol fragments attached to the squaric core, leading to a complete loss of enantioselectivity. Additionally, comparing the results obtained with squaramides **7f** and **8f**, and those with the free aminoalcohol **2f**, it is clear that the enantioselectivity achieved with **8f** is due to the presence of the 2-aminobutanol moiety.

Consequently, we could not obtain arguments about the positive influence of the squaric acid fragment as a backbone of the synthesized ligands on the enantioselectivity. The free aminoalcohols provided better results in all cases. Therefore, additional experiments were performed to study the influence of the reaction conditions on the reduction of **19** by using aminoalcohol **2c** (Table 2). The general conclusion is that prolonged time for catalyst system formation (compare entries 1 and 2, Table 2), increased temperature (compare entries 5 and 6, Table 2) and shorter reduction times lead to better enantioselectivities in both cases. The preference towards the *S*-enantiomer using this catalytic system can be explained by a plausible transition state (see Supplementary Materials), where two possible adducts (*cis* and *trans*) regarding the orientation of the ethyl substituent and BH_3_ are considered.

It is important to note that the applied conditions for the catalyst system formation cannot be considered as sufficient for oxazaborolidine formation, taking into account the prolonged reaction times and higher temperature described in the literature for this process [10,15,16,17,19]. Nevertheless, the species formed by mixing the ligands with BH_3_•SMe_2_ are obviously catalytically active (with different efficiency), and it is highly justified to study the occurring in situ processes through NMR experiments in detail.

### 2.2. NMR Study of the Catalytic Systems

With the aim to better understand the reasons behind the significantly lower enantioselectivity obtained using chiral squaric acid derivatives as ligands compared to the corresponding free chiral aminoalcohols, more detailed studies were performed. According to literature data, different reactive intermediates, as well as monomeric and dimeric oxazaborolidine species, may exist in dynamic equilibrium in the reaction mixture depending on the ligand structure and concentration, type of the solvent and borane reagent [61,62,63,64,65,66,67]. The possible presence of these equilibrating species in situ is a key feature of the catalyst system influencing its performance and activity. Therefore, we suggest that the stability of the intermediate species generated during the first step of the catalytic system formation by mixing the ligand and the borane reagent at room temperature may influence the activity of the oxazaborolidine catalyst presumably generated in the next step upon heating. To clarify the role of the ligand structure on the formation and stability of these intermediate species, a combination of 1D (^1^H and ^10/11^B) and 2D (^1^H COSY, ^1^H-^13^C HSQC, ^1^H-^15^N HMBC/HSQC and ^1^H DOSY) NMR techniques was used. The comparative study was performed in situ by mixing BH_3_•SMe_2_ with three different types of ligands in a 1:1 molar ratio at room temperature: (i) free 2-aminobutanol **2c** (without squaric acid fragment), (ii) structure **5**, representing a squaric acid derivative incorporating both the amido fragment and hydroxy group, and (iii) structure **6**, representing a squaric acid derivative containing the amido fragment only. The choice of ligands was based on the results for asymmetric borane reduction of α-chloroacetophenone (Table 1), demonstrating that among the used ligands, the highest enantioselectivity was achieved with aminoalcohols **2c** and **17**, while the squaric acid derivatives **5** and **6** are among the ligands providing moderate and very poor enantioselectivity.

The ^1^H spectra of ligands **2c**, **5** and **6** (0.1 M solutions) and the assignment of the signals are provided in the Supplementary Materials (Figures S1–S3). The ^1^H spectra of all ligands show sharp well-resolved resonances for all structural fragments. In the spectrum of **2c**, the NH_2_ and OH protons have a broad resonance at around 1.68 ppm due to their labile nature, while sharp signals were observed for OH and/or NH protons in the spectra of the two squaramide ligands **5** and **6**. The ^1^H spectra of the two squaramide derivatives **5** and **6** show two sets of signals for each structural fragment of the molecules, indicating the presence of two isomers (Supplementary Figures S2 and S3). Previous studies showed that in squaramide solutions, there is a slow equilibrium between the *syn*- and *anti*-isomers due to hindered rotation around the squaramide bond [54]. The signals of *syn*-N-substituents are shifted downfield relative to those of *anti*-N-substituents due to the paramagnetic anisotropy of the carbonyl group from the squaramide moiety. Thus, the major conformer of ligands **5** and **6** was assigned to the *syn*-form, which is in agreement with the literature data for series of squaramides where the *syn*-rotamer was always found to be favored over the *anti*-rotamer [68]. The NH signals were assigned by ^1^H-^15^N HSQC and ^1^H-^15^N HMBC experiments, which allow observation of ^1^H-^15^N indirect spin-spin coupling over one, two or three bonds. Examples of the 2D ^1^H-^15^N spectra are provided in the Supplementary Materials (Figures S4 and S5).

**Figure 1:**
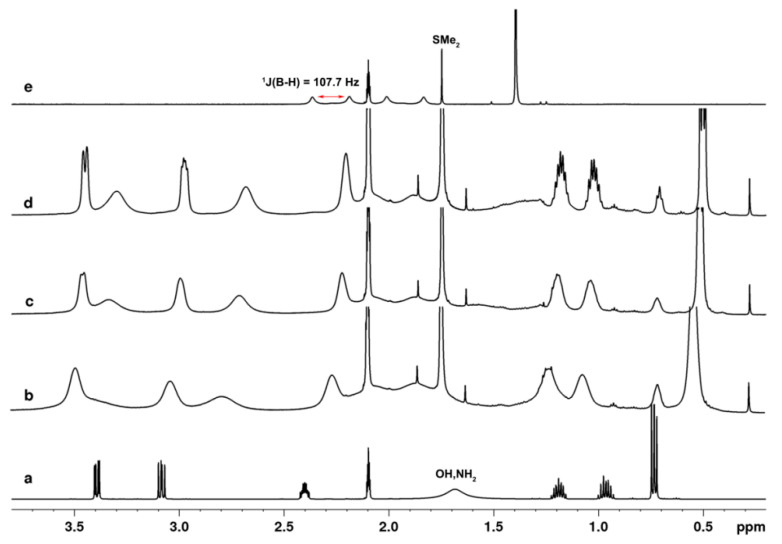
^1^H NMR spectra of: (**a**) pure ligand **2c** (0.1 M) in toluene-d_8_, and mixture of 0.1 M of **2c** and BH_3_•SMe_2_ (1:1 molar ratio) in toluene-d_8_ measured at different time intervals after sample preparation: (**b**) 15 min, (**c**) 4 h 30 min and (**d**) 4 days. The ^1^H spectrum of BH_3_•SMe_2_ in toluene-d_8_ is also shown for comparison (**e**).

The results presented in Table 1 clearly demonstrate that all free aminoalcohols used as ligands provided higher enantioselectivity in the studied asymmetric borane reductions as compared to the corresponding squaramide-based ligands. With the aim to clarify the role of the ligand structure on the formation and stability of the intermediates, the interaction of BH_3_•SMe_2_ with aminoalcohol **2c** was initially investigated. The ^1^H NMR spectra of ligand **2c** in toluene-d_8_ before and after the addition of BH_3_•SMe_2_, measured at different time intervals, are presented in Figure 1.

**Scheme 4:**
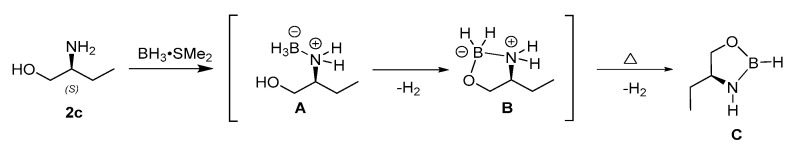
Possible intermediates (**A** and **B**) generated in situ at room temperature after mixing ligand **2c** and BH_3_•SMe_2_. In a next step, upon heating the ligand-borane mixture, oxazaborolidine **C** is formed after the release of H_2_.

It is obvious that upon addition of BH_3_•SMe_2_, the signals of **2c** are broader and shifted compared to the resonances of the pure ligand. This broadening of the ligand signals indicates that there is an interaction between **2c** and BH_3_•SMe_2_, leading to the formation of new species. The interaction is accompanied by the release of SMe_2_, as evidenced by the appearance of the singlet at 1.75 ppm having the same chemical shift value as the free SMe_2_. A small amount of SMe_2_ was also detected in the solution of pure BH_3_•SMe_2_ in toluene-d_8_ (Figure 1e). We suggest that the first step of the interaction of ligand **2c** and BH_3_•SMe_2_ proceeds with coordination and formation of species of type **A**, followed by protolysis and evolution of species of type **B** (Scheme 4). These species most probably exist simultaneously in the reaction mixture; however, their discrimination is not possible based on ^1^H spectra due to signals overlapping. Clear evidence for their formation is the appearance of the two additional signals at 2.80 and 3.37 ppm. These resonances were assigned to the protons from the NH_2_ group of the chiral ligand bound through B−N coordination. In the ligand-borane complexes **A** and **B**, these protons are less mobile compared to the free ligand, which makes their observation as separate signals possible. In addition, these protons are diastereotopic, hence they have different chemical shifts.

**Figure 2:**
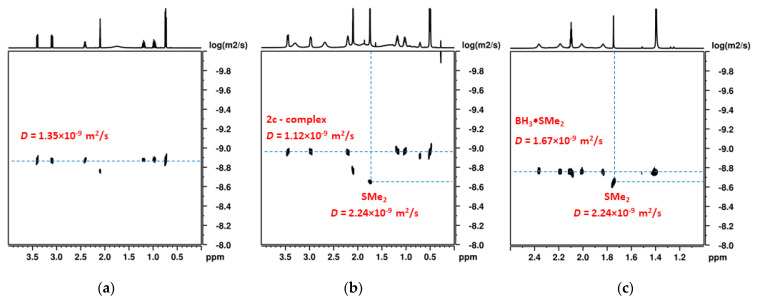
^1^H DOSY spectra of: (**a**) pure ligand **2c** in toluene−d_8_, (**b**) **2c** in the presence of BH_3_·SMe_2_ and (**c**) BH_3_•SMe_2_ in toluene-d_8_.

The ^1^H spectra measured at different time intervals show that with time, the linewidth of all signals decreases, and the signals shift slightly up-field with respect to their chemical shifts in the spectrum measured immediately after mixing (Figure 1b–d). The sharpening of the signals with the increase of the incubation time suggests that the reaction probably proceeds slowly towards the more rigid **B** species. The formation of intermediates **A** and **B** at room temperature, which could be considered as precursors for the oxazaborolidine (**C**) after heating and subsequent release of H_2_, was further evidenced by ^1^H DOSY NMR (Figure 2) and HRMS analysis (see Supplementary Materials) of the mixture of **2c** and BH_3_•SMe_2_. The DOSY spectrum shows that the diffusion coefficient of **A** and **B** (D = 1.12 × 10^−9^ m^2^s^−1^) was lower as compared to the diffusion coefficient of the free ligand (D = 1.35 × 10^−9^ m^2^s^−1^). The diffusion coefficient of 2.24 × 10^−9^ m^2^s^−1^ determined at the chemical shift of the signal at 1.75 ppm was identical to the one of the pure SMe_2_ in toluene-d_8_. The same diffusion coefficient was determined at the chemical shift of this signal from the DOSY spectrum of pure BH_3_•SMe_2_ in toluene-d_8_.

The release of SMe_2_ was also observed in previous studies where the equilibrium dissociation of BH_3_•SMe_2_ was proposed as a first step of the reaction mechanism in the reduction of nitriles by BH_3_•SMe_2_ [69]. The broad hump in the region from 1.5 to 2.3 ppm (Figure 1b) is assigned to the BH_3_ protons. The fine structure of the signal observed in the ^1^H spectrum of the pure BH_3_•SMe_2_ resulting from ^1^J(^11^B-^1^H) = 107.7 Hz indirect spin–spin coupling (Figure 1e) is lost due to involvement of the borane protons in the formation of the new species with lower symmetry of the boron environment.

**Figure 3:**
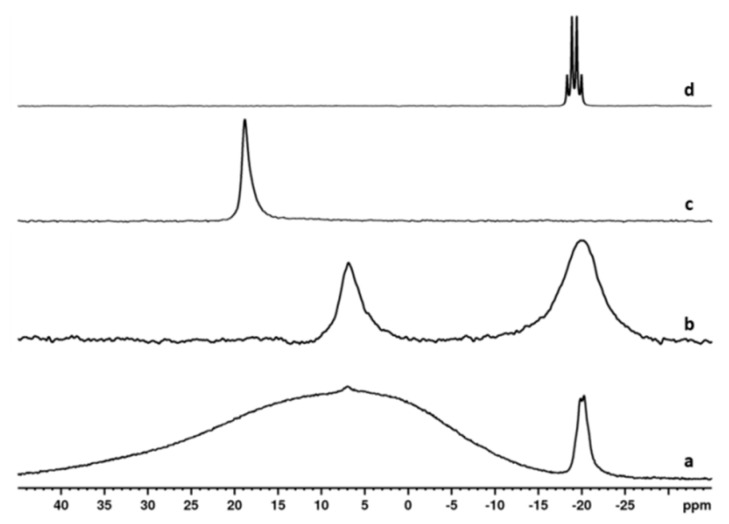
(**a**) ^11^B NMR spectrum of mixture of **2c** and BH_3_•SMe_2_ in toluene-d_8_ at 20 °C, (**b**) ^10^B NMR spectrum of mixture of **2c** and BH_3_•SMe_2_ in toluene-d_8_ at 20 °C, (**c**) ^10^B spectrum of **2c** and BH_3_•SMe_2_ mixture in toluene-d_8_ at 50 °C and (**d**) ^11^B NMR spectrum of BH_3_•SMe_2_ in toluene-d_8_.

To prove the simultaneous presence of species **A** and **B**, ^11^B and ^10^B spectra of the mixture were acquired. The ^11^B spectrum (Figure 3a) shows one signal at −19.97 ppm with an unresolved quartet-like structure (^1^J_B-H_ = 74 Hz) that was assigned to N-BH_3_ coordinated species **A**, according to literature data [66]. The second signal at around 7 ppm was assigned to N-B-O complex **B** [70]. Supplementary Figure S6 shows a comparison of the ^11^B spectra of the mixture of ligand **2c** and BH_3_•SMe_2_ in toluene-d_8_ with the spectra of pure BH_3_•SMe_2_ in toluene-d_8_. The high-field shift of about 1 ppm and the broadening of the quartet for BH_3_ due to its involvement in complex **A** is clearly visible. The application of broadband proton decoupling resulted in collapse of the multiplet structure and narrowing of the signal (Supplementary Figure S6b,d). Unfortunately, the broad background signal due to probe-head components covering the range from −15 to 40 ppm hampered the clear observation of the signal originating from the complex **B**. Previous studies demonstrated that the unwanted background signal is inherently suppressed in ^10^B NMR spectra [71]. Figure 3b shows the ^10^B spectrum of the mixture, where the signal of the complex **B** at around 7 ppm is now clearly visible.

The drawbacks of ^10^B spectra (I^10^B = 3, Q^10^B = 8.46 fm^2^, NA = 19.9%) are the lower sensitivity and the generally broader signals as compared with ^11^B spectra (I^11^B = 3/2, Q^11^B = 4.06 fm^2^, NA = 80.1%) due to the respectively lower natural abundance and higher quadrupolar effect of the ^10^B isotope. Nevertheless, ^10^B spectra present a convenient alternative of ^11^B spectra in situations where the contribution of the background signal cannot be avoided. Importantly, the ^10/11^B spectra are not quantitative, since the relaxation of the quadrupolar B nuclei strongly depends on the symmetry of the boron environment. For example, the relaxation of the boron in structures with higher symmetry such as BH_3_•SMe_2_ is generally slower as compared to the relaxation in species with lower symmetry such as **A** and **B**.

In a next step, the mixture of **2c** and BH_3_•SMe_2_ was heated in situ in the NMR tube at a temperature of 50 °C. The ^10^B spectrum (Figure 3c) now shows only one signal at 18.82 ppm that is characteristic of monomeric oxazaborolidine **C** [10,72,73,74]. Therefore, we consider this observation as evidence that upon heating of the reaction mixture, the intermediates **A** and **B** are subsequently transformed to oxazaborolidine catalyst (**C**) after evolution of H_2_. To the best of our knowledge, this is the first experimental proof by NMR for the in situ formation of the oxazaborolidine catalyst.

**Figure 4:**
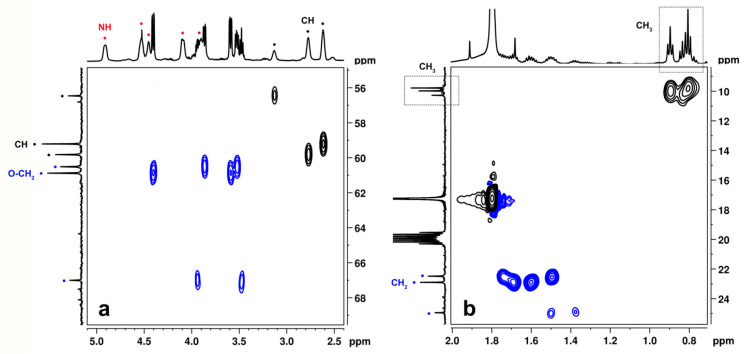
^1^H-^13^C HSQC NMR spectrum of 2 M mixture of ligand **2c** and BH_3_•SMe_2_ in toluene-d_8_. The correlation peaks originating from the three sets of 2-aminobutanol-based structural fragments are indicated as follows: (**a**) the OCH_2_ groups (blue dots) and the CH(NH_2_) groups (black dots), and (**b**) the CH_2_ groups (blue dots) and CH_3_ groups enclosed in the dashed rectangle. The six protons from the three NH_2_ groups (red dots) were identified due to a lack of cross-peaks with C-atoms over one bond.

**Figure 5:**
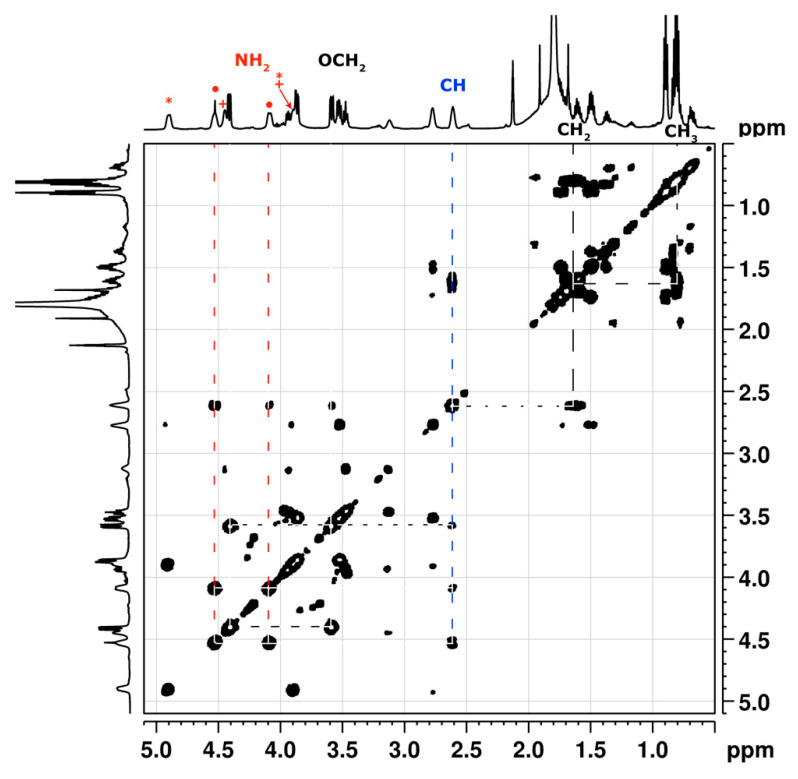
COSY NMR spectrum of 2 M mixture of ligand **2c** and BH_3_•SMe_2_ in toluene-d_8_ showing the presence of three 2-aminobutanol-based spectral patterns. As an example, the dashed lines show the cross-peak connectivities between the structural fragments within one of the 2-aminobutanol-based complexes. The signals of the two diastereotopic protons of the three NH_2_ groups are shown with red symbols.

The influence of the concentration on the nature and stability of the intermediates was studied in a next step. The ^1^H and ^13^C spectra of the mixture with 2 M concentration of the ligand at room temperature are presented in the Supplementary Materials, Figure S7a,b. The spectra indicate that at higher ligand concentrations, there is a complex equilibrium between at least three different species involving **2c**. This is further indicated by ^1^H-^13^C HSQC and ^1^H COSY spectra of the mixture, which display three separate sets of O-CH_2_, N-CH and CH_2_-CH_3_ spectral patterns (Figure 4 and Figure 5).

**Figure 6:**
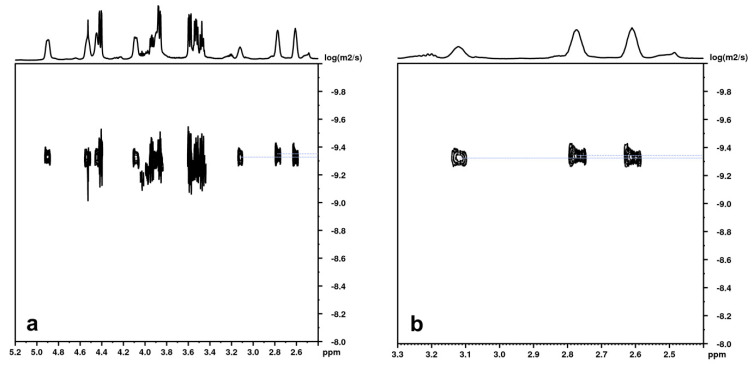
^1^H DOSY spectrum of the 2 M mixture of ligand **2c** and BH_3_•SMe_2_ in toluene-d_8_: (**a**) full spectrum, and (**b**) expanded area with the three sets of CH protons used for determination of the diffusion coefficients of the three complexes. The diffusion coefficients were calculated by fitting the diffusion profile of each signal (the normalized signal intensity as a function of the gradients’ strength) to a variant of the Stejskal–Tanner equation.

The signals of the NH_2_ protons could be clearly identified from the ^1^H-^13^C HSQC spectrum due to the lack of correlation cross-peaks with C-atoms over one bond, thus confirming the formation of complexes with preserved NH_2_ moiety. The DOSY spectrum of the mixture (Figure 6) shows that two of the complexes have very close diffusion coefficients, with an averaged value of 4.48 × 10^−10^ m^2^s^−1^, while the third complex has a slightly higher diffusion coefficient of 4.68 × 10^−10^ m^2^s^−1^. The diffusion coefficients of the intermediates in the mixture with higher concentration are by a factor of two lower as compared to the value (D = 1.12 × 10^−9^ m^2^s^−1^) measured in the mixture with lower concentration (see above and Figure 2). The diffusion coefficient of the SMe_2_ was identical in both samples, ruling out any possibility of viscosity-induced differences of the D values measured in the two samples. Therefore, our interpretation is that at higher concentrations, dimeric intermediates with different coordination modes are present in dynamic equilibrium. Different types of dimeric oxazaborolidine-based structures have been proposed in the literature, including the (N,N-adducts), (N,O-adducts) and (O,O-adducts). The small difference between the diffusion coefficients of the dimeric species could be explained by the different shape of the intermediates resulting from the different coordination modes [61,62,72].

Four broad signals at 24, 9.6, 6.7 and −19.8 ppm were observed in the ^10^B spectrum of the mixture (Supplementary Figure S8). The chemical shifts of the signals are generally consistent with the values reported for some cyclic organoborane Lewis-base adducts and borane chelate complexes with B←N and/or B←O coordinated moieties [75,76].

Further, we investigated the influence of the solvent on the possibility for formation of different intermediates between **2c** and BH_3_•SMe_2_ in the low-concentration regime (0.1 M), similar to the reaction conditions. For this purpose, the samples were dissolved in THF-d_8_. Contrary to the sample in toluene-d_8_ (Figure 1), the ^1^H NMR spectra show that in THF-d_8_, several stable intermediates were formed, as demonstrated by the increased numbers of signals (Supplementary Figure S9). By analysis of the correlation cross-peaks observed in the COSY spectrum (Supplementary Figure S9c), we identified at least three main spin systems based on the 2-aminobutanol fragment CH_2_-CH-CH_2_. The formation of intermediates with different degrees of association was also confirmed by ^1^H DOSY. The DOSY spectrum of the mixture in THF-d_8_ shows the presence of two main components with diffusion coefficients of 1.02 × 10^−9^ m^2^s^−1^ and 0.81 × 10^−9^ m^2^s^−1^ that are significantly lower as compared to the diffusion coefficient of the pure ligand in THF-d_8_ (1.68 × 10^−9^ m^2^s^−1^). These results imply that in THF, the formation of the dimeric species occurs even at lower concentrations.

**Figure 7:**
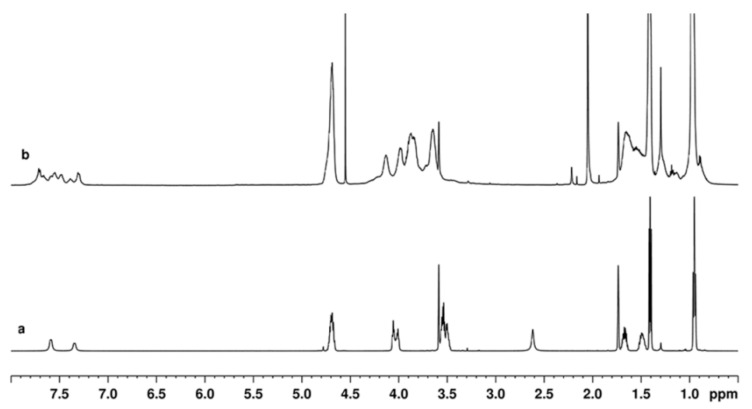
^1^H NMR spectra of: (**a**) pure ligand **5** in THF-d_8_, and (**b**) mixture of **5** and BH_3_•SMe_2_ in THF-d_8_.

With the aim to assess the influence of the squaric acid fragment on the formation and performance of the catalytic system, we investigated ligand **5** containing both the squaric acid skeleton and aminoalcohol function. The ^1^H spectrum of the mixture of ligand **5** and BH_3_•SMe_2_ in THF-d_8_ shows shifting and broadening of the ligand signals (Figure 7b). The number of signals was also increased, which indicated that the interaction between ligand **5** and BH_3_•SMe_2_ resulted in the formation of multiple species. Due to the complexity of the spectrum, it was not possible to draw conclusions about the structure of these adducts.

**Figure 8:**
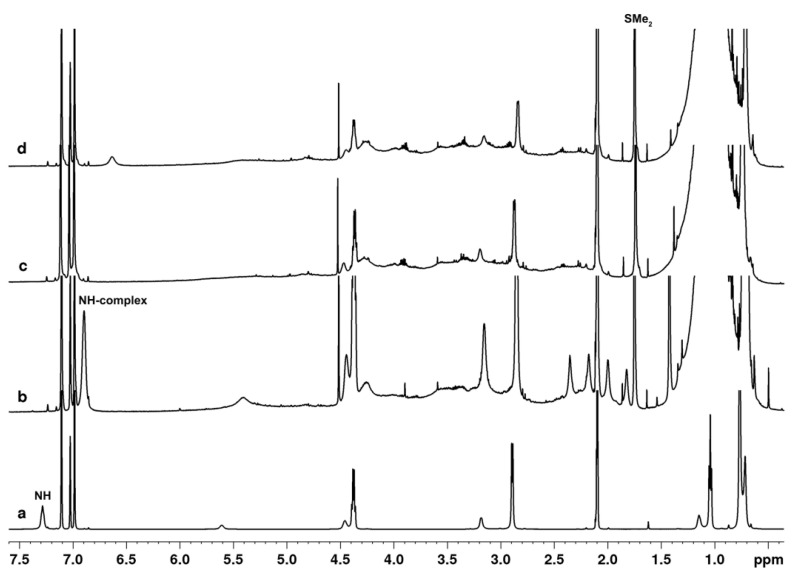
^1^H NMR spectra of: (**a**) pure ligand **6** in toluene-d_8_, and mixture of **6** and BH_3_•SMe_2_ in toluene-d_8_ measured at different time intervals after sample preparation: (**b**) 15 min, (**c**) 3 h 30 min and (**d**) 5 h.

The ^1^H NMR spectra of pure ligand **6** in toluene-d_8_ and of **6** in the presence of BH_3_•SMe_2_ measured at different time intervals after sample preparation are presented in Figure 8. The spectra demonstrate that in the presence of BH_3_•SMe_2_, the signal of the NH proton of the ligand is shifted up-field by 0.39 ppm and all other signals of the ligand are broader as compared to the spectra of the pure ligand (Figure 8b). The ^1^H spectra measured several hours after mixing show the appearance of a number of unresolved additional signals. In parallel to that, the intensity of the signals of the ligand gradually decreased with time, indicating the occurrence of degradation reactions simultaneously with the possible formation of intermediates (Figure 8c,d).

**Figure 9:**
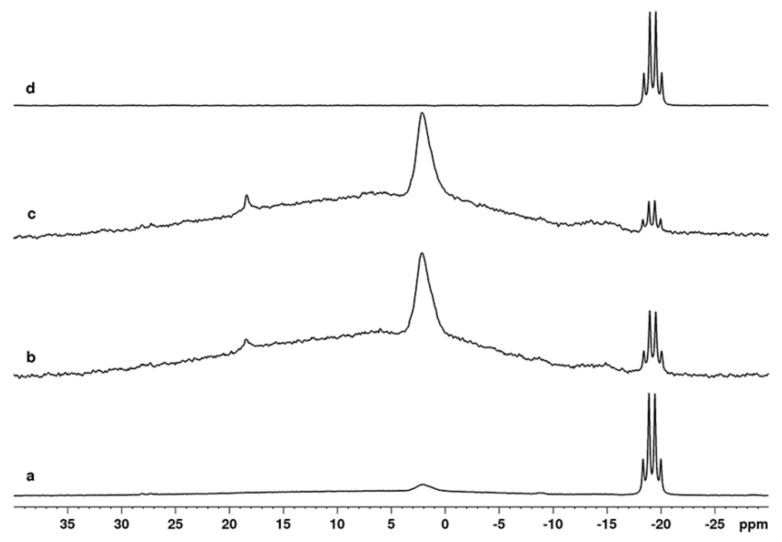
^11^B NMR spectra of mixture of ligand **6** and BH_3_•SMe_2_ in toluene-d_8_ measured at different time intervals after sample preparation: (**a**) 15 min, (**b**) 3 h 30 min and (**c**) 5 h. The ^11^B spectrum of BH_3_•SMe_2_ in toluene-d_8_ is also shown for comparison (**d**).

The formation of intermediate between the ligand **6** and BH_3_•SMe_2_ was also confirmed by ^11^B NMR spectroscopy. The ^11^B spectra of the mixture show the presence of a characteristic quartet at −19.17 ppm as well as an additional signal at 2.1 ppm assigned to the complex (Figure 9). The involvement of BH_3_•SMe_2_ in side reactions was also confirmed since the intensity of the signal at −19.17 ppm decreased with time (Figure 9b,c). Thus, the NMR data point out that the stability of the possible intermediate formed between squaric acid derivative **6** containing the amido function only and BH_3_•SMe_2_ is very poor.

The overall analysis of the comparative NMR data for the three model ligands shows that the highest stability was demonstrated for the intermediates including aminoalcohol ligand **2c**, where the squaric acid fragment is missing. The complexes between ligand **5** and borane remained stable within the next 10 h, contrary to the complex with the participation of ligand **6** that also contained the squaric acid fragment, but only the amide function in the side chain.

Therefore, the stability of the intermediates follows the row **2c** > **5** > **6**, which correlates with the observed trend in the enantioselectivity—83% *ee* (**2c**), 31% *ee* (**5**) and 0% *ee* (**6**)—in catalytic asymmetric borane reduction of α-chloroacetophenone by applying these chiral squaric acid amides and aminoalcohols as ligands (see Table 1). Comparison of the results for ligands **5** and **6** implies that the simultaneous presence of hydroxy and amido functionalities in the structure of ligand **5** contributes to the better stability of the intermediates and impedes the process of ligand destruction eventually induced by the squaric moiety.

Considering that under the real reaction conditions the amount of the reducing borane is much higher, we can suggest that the squaramide ligands undergo much faster degradation due to unspecific borane reduction, thus preventing the formation of stable intermediates and their subsequent transformation to catalytic oxazaborolidine species upon heating. Therefore, we can conclude that squaramides are poor ligands for the asymmetric CBS reduction because they degrade under the reaction conditions used in our study, unlike simpler aminoalcohols, which can form competent oxazaborolidines.

## 3. Materials and Methods

### 3.1. Chemistry

The synthesis of all compounds has been performed using commercially available chemicals and materials (amines **2a** and **11a**, aminoalcohols **2a**–**d** and **2h**–**j**, 3,4-diethoxycyclobut-3-ene-1,2-dione (**1**), ketone **19** and BH_3_•SMe_2_) from Sigma-Aldrich (St. Louis, MI, USA), Merck (Tokyo, Japan), Fluka (Buchs, Switzerland), Acros (Waltham, MA, USA), Alfa Aesar (Haverhill, MA, USA) and Tokyo Chemical Industry (TCI, Tokyo, Japan). Commercially available chiral amines and aminoalcohols were purchased from Sigma-Aldrich with *ee* > 98. Aminoalcohols **2e**–**g** and amine **11b** were prepared according to known procedures [57,77,78,79]. The catalytic reactions were carried out using α-chloroacetophenone freshly crystalized from heptane.

All compounds were obtained with high chemical purity using column chromatography or crystallization. The structures and configurations of all new compounds were unambiguously determined by 1D and 2D NMR experiments (including detailed descriptions of ^1^H and ^13^C chemical shifts and coupling constants), mass spectrometry, optical rotation, elemental analysis and melting point temperatures.

The organometallic reactions were carried out in flame-dried Schlenk flasks under an argon atmosphere. Tetrahydrofuran (THF) was distilled over sodium/benzophenone. Toluene was distilled over Na[Et_4_Al]. For thin-layer chromatography (TLC), aluminum sheets pre-coated with silica gel 60 F_254_ (Merck) were used. Flash column chromatography was carried out using silica gel 60 (0.040–0.063 mm, 230–400 mesh ASTM, Merck). Commercially available solvents for reactions, TLC and column chromatography were used after distillation—petroleum ether (PE), diethyl ether (Et_2_O), dichloromethane (DCM), methyl *tert*-butyl ether (MTBE), tetrahydrofuran (THF), methanol (MeOH), ethanol (EtOH), isopropyl alcohol (*i*-PrOH), ethylacetate (EtOAc), cyclohexane (CH) and acetone. Melting points were determined in capillary tubes on an MPA100 OptiMelt automated melting point system (SRS, Sunnyvale, CA, USA) without corrections. Mass spectra (MS) were recorded on a Thermo Scientific High-Resolution Magnetic Sector MS DFS by chemical ionization (CI), and are reported as fragmentation in m/z with relative intensities (%). Mass analysis of intermediates’ formation was performed on a Q Exactive Plus Hybrid Quadrupole-Orbitrap Mass Spectrometer (ESI HRMS), Thermo Scientific, in positive mode. Optical rotation [α] measurements were obtained using Perkin–Elmer 241 and Jasco-P-2000 polarimeters. The *ee* values were determined on a Shimadzu GC-17A gas chromatograph with an FID detector and chiral column MN-Hydrodex β-TBDAc (0.25 mm × 25 m). The column temperature was 160 °C (isothermal). The injector and detector temperatures were 240 °C. Helium was the carrier gas at a flow rate of 1 mL/min, split 1:20. Retention times: t_R_ = 6.0 min, t_S_ = 6.3 min. Elemental analyses were performed by the Microanalytical Laboratory for Elemental Analysis of the Institute of Organic Chemistry, Bulgarian Academy of Sciences. The numbering of atoms in formulas is not in conformity with the IUPAC names of the compounds. Chemical shifts assigned with an asterisk (*) are tentative. The assignments of protons as H_a_ and H_b_ are tentative.

### 3.2. NMR Spectroscopy

The NMR spectra were recorded on a Bruker Avance II+ 600 (600.01 MHz for ^1^H, 150.87 MHz for ^13^C, 64.46 MHz for ^10^B, 192.51 MHz for ^11^B, 60.81 MHz for ^15^N NMR) spectrometer. In the case of CDCl_3_, TMS was used as an internal standard for chemical shifts (δ, ppm) and ^1^H spectra were calibrated to the signal of TMS (δ = 0.0000). For other deuterated solvents, ^1^H spectra were calibrated to the residual solvent peaks (DMSO-d_6_ δ = 2.50). ^13^C spectra were calibrated in all cases to the residual solvent peaks (CDCl_3_ δ = 77.00, DMSO-d_6_ δ = 39.52). The following additional NMR techniques were used for all compounds: DEPT 135, COSY, HSQC, HMBC and NOESY. ^1^H and ^13^C NMR data are reported as follows: chemical shift, multiplicity (s = singlet, d = doublet, t = triplet, q = quartet, br = broad, m = multiplet), integration, identification and coupling constants (in Hz).

The ^1^H DOSY spectra were measured on a Bruker Avance II+ 600 NMR spectrometer using a 5 mm direct detection dual-broadband probe, with a gradient coil delivering maximum gradient strength of 53 G/cm. All experiments were performed at a temperature of 293 K. The DOSY spectra were measured using a convection compensating double-stimulated echo-based pulse sequence, with monopolar square-shaped gradient pulses. The spectra were acquired with 64 K time domain data points in t2 dimension, 32 gradient strength increments, diffusion delay of 100 ms, gradient pulse length of 2 ms, 16 transients for each gradient step and a relaxation delay of 2 s. The gradient strength was incremented from 4% to typically 70% of the maximum gradient output (from 1.92 to 33.7 G/cm) to achieve optimal signal attenuation. The spectra were processed with exponential window function (line broadening factor 0.2), 64 K data points in F2 and 256 data points in the diffusion dimension. The evaluation of the diffusion coefficients was performed by fitting the diffusion profile (the normalized signal intensity as a function of the gradient strength G) at the chemical shift of selected signals in the DOSY spectrum with an exponential function using the variant of the Stejskal–Tanner equation adapted to the particular pulse sequence used.

The ^10/11^B spectra were measured using a single-pulse experiment using the following acquisition parameters: spectral width 150 ppm, time domain data points 64 K, 90° pulse length 15.4 us, relaxation delay 0.5 s, number of transients typically 4000. The spectra were referenced to 15% BF_3_•Et_2_O (0 ppm) solution in CDCl_3_ used as an external standard.

## 4. Conclusions

We synthesized a series of chiral compounds (squaramides containing N- and O-functionality, and some aminoalcohols) in order to study their efficacy as ligands in the model reduction reaction of α-chloroacetophenone with BH_3_•SMe_2_. Our results clearly demonstrate that the free aminoalcohols were more efficient ligands to induce enantioselectivity (up to 94% *ee*) in comparison to the corresponding squaramides. The catalytic system was studied in situ in reaction mixtures modeling the catalyst formation between BH_3_•SMe_2_ and compounds **2c**, **5** and **6** by means of DOSY and multinuclear 1D and 2D (^1^H, ^10/11^B, ^13^C, ^15^N) NMR spectroscopy. The results obtained imply that the aminoalcohols form stable intermediates after reacting with BH_3_•SMe_2_ at room temperature. Upon heating the reaction mixture at 50 °C, these intermediates are subsequently transformed to oxazaborolidine. It was found that the presence of the squaric acid moiety in the ligand structure contributes to low stability of the intermediates, leading to destabilization of the catalyst system and to its further destruction. Unlike the simple aminoalcohols that remained stable under the reaction conditions used in our study (high excess of the borane source), the squaramide ligands underwent fast nonspecific destruction, which hampered the in situ formation of a stable catalytic system. These results explain the systematically lower enantioselectivities obtained when squaramide derivatives were used as chiral ligands in the reaction of asymmetric borane reduction of α-chloroacetophenone.

## Figures and Tables

**Table 1:** Catalytic asymmetric borane reduction of α-chloroacetophenone by applying chiral squaric acid amides and aminoalcohols as ligands.

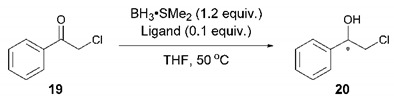							
Squaric Acid Amides				Free Aminoalcohols			
Entry	Ligand	Yield (%)	*ee*^a^ (Config.)	Entry	Ligand	Yield (%)	*ee*^a^ (Config.)
1	**4**	99	53 (*R*)	19	**2b**	98	79 (*R*)
2	**5**	99	31 (*S*)				
3	**7b**	72	39 (*R*)				
4	**8b**	99	42 (*R*)	20	**2c**	97	83 (*S*)
5	**9b**	86	0				
6	**9c**	99	19 (*S*)				
7	**7f**	92	0	21	**2f**	99	9 (*S*)
8	**8f**	99	56 (*R*)				
9	**7e**	95	10 (*R*)	22	**2e**	99	49 (*R*)
10	**8e**	99	49 (*R*)				
11	**9e**	99	35 (*S*)				
12	**7h**	80	13 (*S*)	23	**2h**	80	61 (*S*)
13	**7i**	99	37 (*S*)	24	**2i** ^b^	98	44 (*R*)
14	**7g**	99	56 (*R*)	25	**2g** ^c^	94	79 (*S*)
15	**10**	98	0	26	**6**	96	0
16	**13a**	99	0	-	**-**	-	-
17	**13b**	93	0	-	**-**	-	-
18	**18**	92	26 (*S*)	27	**17**	99	94 (*S*)

^a^ The *ee* values were determined by capillary GC analysis with a chiral column MN-Hydrodex β-TBDAc (0.25 mm × 25 m). Configurations were assigned by comparison of the sign of the specific rotation with that reported in the literature. ^b^ Reference [11]. ^c^ Reference [60].

**Table 2:** Optimization of the reaction conditions for borane reduction of α-chloroacetophenone catalyzed by **2c**.

Entry	Ligand (Equiv.) ^a^	Temperature (°C)	Solvent	Time (min)	Yield (%)	*ee*^c^ (Config.)
1	**2c** (0.1)	50	THF	20	96	83 (*S*)
2	**2c** (0.1) ^b^	50	THF	5	91	86 (*S*)
3	**2c** (0.1)	25	THF	30	92	73 (*S*)
4	**2c** (0.1)	0	THF	240	99	22 (*S*)
5	**2c** (0.1)	50	Toluene	20	97	70 (*S*)
6	**2c** (0.1)	100	Toluene	5	91	80 (*S*)

^a^ Catalyst system was formed by mixing the ligand and BH_3_•SMe_2_ in the corresponding solvent and stirring the mixture for 2 h at room temperature and 1 h at the corresponding reaction temperature. ^b^ The catalyst system was formed by stirring for 4 h at the reaction temperature. ^c^ The *ee* values were determined by capillary GC analysis with a chiral column MN-Hydrodex β-TBDAc (0.25 mm × 25 m). Configurations were assigned by comparison with the sign of the specific rotation with that reported in the literature.

## Data Availability

Not applicable.

## Supplementary Materials

The following are available online: Details on the preparation, purification, spectral and analytical data for compounds **3**–**10**, **12**, **13**, **17** and **18**, as well as other general comments and methodologies regarding synthetic procedures and NMR (Figures S1–S9) experiments are introduced in detail in the Supplementary Materials.

## Sample Availability

Samples of the compounds are not available from the authors.
