# Supplementary material for: Chiral Aminoalcohols and Squaric Acid Amides as Ligands for Asymmetric Borane Reduction of Ketones: Insight to In Situ Formed Catalytic System by DOSY and Multinuclear NMR Experiments
Source: Molecules. 2021 Nov 14;26(22):6865. doi: 10.3390/molecules26226865 (PMC8624562; doi:10.3390/molecules26226865)
Supplement: Supplementary file 1 [file molecules-26-06865-s001.zip › molecules-1439549-supplementary.pdf]

Supplementary Materials

for

Chiral Aminoalcohols and Squaric Acid Amides as Ligands for Asymmetric Borane Reduction of Ketones: Insight to In situ Formed Catalytic System by DOSY and Multinuclear NMR Experiments

Yana Nikolova, Georgi M. Dobrikov, Zhanina Petkova and Pavletta Shestakova

Institute of Organic Chemistry with Centre of Phytochemistry,
Bulgarian Academy of Sciences, Bl. 9, Acad. G. Bonchev Str., Sofia 1113, Bulgaria

Experimental procedures, analytical data and NMR spectra

Preparation of 3-(benzylamino)-4-ethoxycyclobut-3-en-1,2-dione (3)

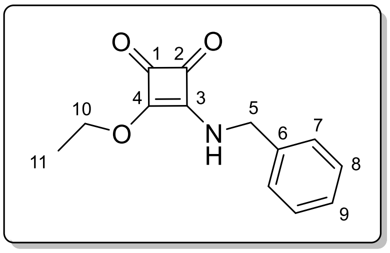

A solution of 3,4-diethoxycyclobut-3-ene-1,2-dione **1** (1.73 mmol, 0.25 ml) and Et_3N (1.73 mmol, 0.24 ml) in 5 ml EtOH was cooled to 0 °C. Benzylamine was added (1.73 mmol, 0.19 ml) and the reaction mixture was stirred at room temperature for 24 h. The solvent was removed under reduced pressure and the crude product was purified by column chromatography (30 g silica gel, eluent: DCM/MTBE = 10:1) to give 0.39 g (99%) **3** as colorless oil.

<u>*Analytical and spectral data:*</u>
MS (ESI) *m/z* (rel. int.): 232 (100, M+1), 204 (82, M−CH_2CH_3). Anal. calcd. for $C_{13}H_{13}NO_3$ (231.25): C, 67.52; H, 5.67; N, 6.06. Found: C, 67.55; H, 5.63; N, 6.09 %. ^{1}H NMR (600.1 MHz, DMSO-d$_6$, 373 K): δ = 8.79 (br s, 1H, N<u>H</u>). 7.34-7.38 (m, 2H, H-8), 7.27-7.33 (m, 3H, H-7, H-9), 4.67 (q, 2H, H-10, *J* = 7.0 Hz), 4.60 (br s, 2H, H-5), 1.38 (t, 3H, H-11, *J* = 7.0). ^{13}C NMR (150.9 MHz, DMSO-d$_6$, 373 K): δ = 188.72* (1C, C-1), 182.04* (1C, C-2), 176.56* (1C, C-3), 172.24* (1C, C-4), 137.74 (1C, C-6), 127.92 (2C, C-8), 126.86 (2C, C-7), 126.81 (1C, C-9), 68.25 (1C, C-10), 46.72 (1C, C-5), 14.82 (1C, C-11).

Preparation of squaric monoamides 4 and 5

General procedure:

A solution of 3,4-diethoxycyclobut-3-ene-1,2-dione **1** (7.04 mmol, 1.03 ml) and Et_3N (7.74 mmol, 1.08 ml) in 20 ml EtOH was cooled to 0 °C. Then the corresponding enantiomer of 2-aminobutan-1-ol **2b** or **2c** (7.04 mmol, 0.66 ml) was added and the resulting mixture was stirred at room temperature for 24 h. The solvent was removed under reduced pressure and the crude product was purified by column chromatography (75 g silica gel, eluent: DCM/acetone = 5:1) to give 1.48 g (99%) **4** or **5** as colorless oil.

3-ethoxy-4-(((*R*)-1-hydroxybutan-2-yl)amino)cyclobut-3-ene-1,2-dione (4)

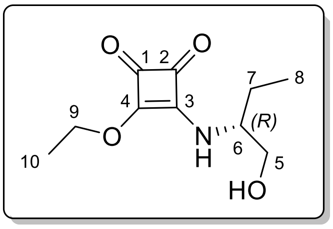

Analytical and spectral data:

$[\alpha]_D^{20}$ = +50.2 (*c* 0.94, $CHCl_3$). MS (CI) *m*/*z* (rel. int.): 214 (M+1, 100). Anal. calcd. for $C_{10}H_{15}NO_4$ (213.23): C, 56.33; H, 7.09; N, 6.57. Found: C, 56.37; H, 7.02; N, 6.53 %. ^1H NMR (600.13 MHz, $CDCl_3$, 298 K): δ = 7.13 (br d, 1H, NH, *J* = 8.9 Hz), 4.84-4.71 (m, 2H, H-9), 3.83-3.76 (m, 1H, H_a-5), 3.73-3.67 (m, 1H, H_b-5), 3.67-3.57 (m, 1H, H-6), 2.81 (br s 1H, OH), 1.71-1.53 (m, 2H, H-7), 1.50-1.42 (m, 3H, H-10), 1.02-0.94 (m, 3H, H-8). ^{13}C NMR (150.92 MHz, $CDCl_3$, 298 K): δ = 189.86* (1C, C-1), 182.56* (1C, C-2), 177.20* (1C, C-3), 172.46* (1C, C-4), 69.90 (1C, C-9), 64.23 (1C, C-5), 58.94 (1C, C-6), 24.89 (1C, C-7), 15.82 (1C, C-10), 10.33 (1C, C-8).

3-ethoxy-4-(((*S*)-1-hydroxybutan-2-yl)amino)cyclobut-3-ene-1,2-dione (5)

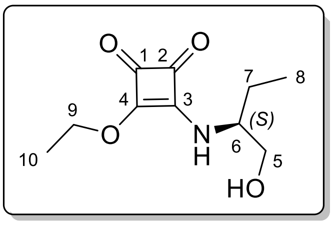

Analytical and spectral data:

$[\alpha]_D^{20}$ = −56.1 (*c* 1.01, $CHCl_3$). MS (CI) *m*/*z* (rel. int.): 214 (M+1, 100). Anal. calcd. for $C_{10}H_{15}NO_4$ (213.23): C, 56.33; H, 7.09; N, 6.57. Found: C, 56.27; H, 7.18; N, 6.52 %. ^1H NMR (600.13 MHz, $CDCl_3$, 293 K): δ = 7.27 (br d, 1H, NH, *J* = 7.3 Hz), 4.83-4.68 (m, 2H, H-9), 3.78 (dd, 1H, H_a-5, *J* = 11.2 Hz, *J* = 2.9 Hz), 3.73-3.56 (m, 2H, H_b-5, H-6), 3.01 (br s 1H, OH), 1.69-1.52 (m, 2H, H-7), 1.45 (t, 3H, H-10, *J* = 7.0 Hz), 0.96 (t, 3H, H-8, *J* = 7.4 Hz). ^{13}C NMR (150.92 MHz, $CDCl_3$, 293 K): δ = 189.88* (1C, C-1), 182.52* (1C, C-2), 177.12* (1C, C-3), 172.45* (1C, C-4), 69.88 (1C, C-9), 64.15 (1C, C-5), 58.98 (1C, C-6), 24.84 (1C, C-7), 15.82 (1C, C-10), 10.34 (1C, C-8).

Preparation of (*S*)-3-((3,3-dimethylbutan-2-il)amino)-4- ethoxycyclobut-3-en-1,2-dione (6)

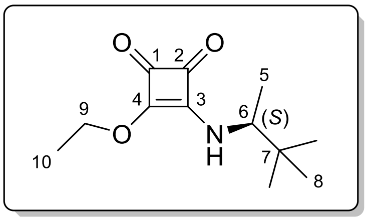

A solution of 3,4-diethoxycyclobut-3-ene-1,2-dione **1** (2.94 mmol, 0.43 ml) and Et_3N (2.94 mmol, 0.41 ml) in 15 ml EtOH was cooled to 0 °C. 2-amino-2-methylpropan-1-ol was added (2.94 mmol, 0.39 ml) and the reaction mixture was stirred at room temperature for 24 h. The solvent was removed under reduced pressure and the crude product was purified by column chromatography (30 g silica gel, eluent: PE/EtOAc = 2:1) to give 0.64 g (93%) **6** as colorless crystals.

Analytical and spectral data:

m.p. 133-135 °C. $[\alpha]_D^{20}$ = −5.3 (*c* 1.08, $CHCl_3$). MS (ESI) *m*/*z* (rel. int.): 226 (100, M+1), 198 (23, M−CH_2CH_3), 142 (60, M−$CH(CH_3)$*t*-Bu), 114 (63, M−CH_2CH_3, $CH(CH_3)$*t*-Bu). Anal. calcd. for $C_{12}H_{19}NO_3$ (225.29): C, 63.98; H, 8.50; N, 6.22. Found: C, 63.94; H, 8.55; N, 6.20 %. ^1H NMR (600.1 MHz, $CDCl_3$, 293 K): δ = 5.93 (br d, 1H, N<u>H</u>, *J* = 8.6 Hz), 4.85-4.70 (m, 2H, H-9), 3.57-3.49 (m, 1H, H-6), 1.46 (t, 3H, H-10, *J* = 7.0 Hz), 1.21 (d, 3H, H-5, *J* = 6.8 Hz), 0.92 (br s, 9H, H-8). ^{13}C NMR (150.9 MHz, $CDCl_3$, 293 K): δ = 189.24* (1C, C-1), 182.81* (1C, C-2), 176.51* (1C, C-3), 171.99* (1C, C-4), 69.60 (1C, C-9), 59.95 (1C, C-6), 34.91 (1C, C-7), 25.75 (3C, C-8), 16.57 (1C, C-5), 15.89 (1C, C-10).

Preparation of squaric diamides 7-10

General procedure:

A solution of monoamide **3-6** (1.0 equiv) in 5 ml EtOH was treated with Et_3N (1.1 equiv). Then the requisite aminoalcohol (1.0 equiv) was added and the resulting mixture was stirred at room temperature for 24 h. The solvent was removed under reduced pressure and the crude product was purified by column chromatography or crystallization.

3-(benzylamino)-4-(((*R*)-1-hydroxybutan-2-yl)amino)cyclobut-3-ene-1,2-dione (7b)

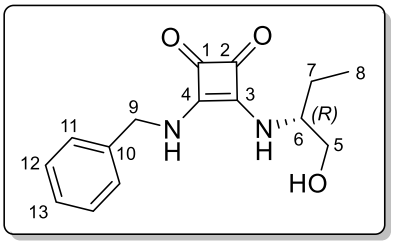

Following the general procedure, the crude product was obtained and then purified by simple filtration and washing with PE to give 0.24 g (93%) **7b** as colorless crystals.

Analytical and spectral data:

m.p. 252-254 °C. $[\alpha]^{20}_{D}$ = +19.7 (*c* 1.00, DMSO). MS (CI) *m/z* (rel. int.): 275 (M+1, 100). Anal. calcd. for $C_{15}H_{18}N_2O_3$ (274.32): C, 65.68; H, 6.61; N, 10.21. Found: C, 65.77; H, 6.72; N, 10.17 %. ^{1}H NMR (600.1 MHz, DMSO-d$_6$, 313 K): δ = 7.73 (br s, 1H, NH), 7.41-7.33 (m, 4H, H-11, H-12), 7.33-7.27 (m, 1H, H-13), 4.83 (br t, 1H, OH, *J* = 4.9 Hz), 4.79-4.68 (m, 2H, H-9), 3.98-3.75 (m, 1H, H-6), 3.50-3.41 (m, 2H, H-5), 3.25 (s, 1H, NH), 1.65-1.52 (m, 1H, H$_a$-7), 1.52-1.38 (m, 1H, H$_b$-7), 0.87 (t, 3H, H-8, *J* = 7.5 Hz) ^{13}C NMR (150.9 MHz, DMSO-d$_6$, 313 K): δ = 182.34* (1C, C-1), 182.26* (1C, C-2), 168.05* (1C, C-3), 167.16* (1C, C-4), 138.87 (1C, C-10), 128.52 (2C, C-12), 127.43 (2C, C-11), 127.28 (1C, C-13), 63.21 (1C, C-5), 56.87 (1C, C-6), 46.73 (1C, C-9), 24.68 (1C, C-7), 10.00 (1C, C-8).

3-(benzylamino)-4-((((1*R*,2*R*,4*S*)-2-hydroxy-1,3,3-trimethylbicyclo-[2.2.1]heptan-2-yl)methyl)amino)cyclobut-3-ene-1,2-dione (7e)

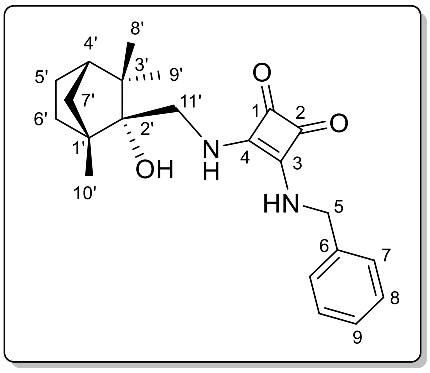

Following the general procedure, the crude product was obtained and then purified by column chromatography (30 g silica gel, eluent: DCM/acetone = 5:1) to give 0.061 g (77%) **7e** as colorless crystals.

Analytical and spectral data:

m.p. 268-269 °C. $[\alpha]^{20}_{D}$ = +18.3 (*c* 0.23, DMSO). MS (CI) *m/z* (rel. int.): 369 (M+1, 100), 351 (M−OH, 65), 216 (M−fenchol, 23). Anal. calcd. for $C_{22}H_{28}N_2O_3$ (368.48): C, 71.71; H, 7.66; N, 7.60. Found: C, 71.83; H, 7.55; N, 7.68 %. ^{1}H NMR (600.1 MHz, DMSO-d$_6$, 293 K): δ = 8.16-8.08 (m, 1H, NH), 7.41-7.36 (m, 2H, H-8), 7.35-7.28 (m, 3H, H-7, H-9), 4.73 (qd, 2H, H-5, *J* = 14.7 Hz, *J* = 6.2 Hz), 4.27 (s, 1H, OH), 3.88 (dd, 1H, H$_a$-11', *J* = 13.4 Hz, *J* = 7.0 Hz), 3.49 (dd, 1H, H$_b$-11', *J* = 13.5 Hz, *J* = 3.9 Hz), 2.04-1.96 (m, 1H, H$_{endo}$-6'), 1.66-1.56 (m, 2H, H$_{endo}$-5', H$_{syn}$-7'), 1.54 (br d, 1H, H-4', *J* = 3.6 Hz), 1.35 (tt, 1H, H$_{exo}$-5', *J* = 12.4 Hz, *J* = 4.7 Hz), 1.09 (d, 1H, H$_{anti}$-7', *J* = 10.0 Hz), 0.97 (overlapped s, 3H, H-8'), 0.96 (overlapped s, 3H, H-10'), 0.93-0.86 (overlapped m, 1H, H$_{exo}$-6'), 0.90 (overlapped s, 3H, H-9'). ^{13}C NMR (150.9 MHz, DMSO-d$_6$, 293 K): δ = 182.99* (1C, C-1), 182.17* (1C, C-2), 167.69* (1C, C-3), 167.57* (1C, C-4), 138.94 (1C, C-6), 128.74 (2C, C-8), 127.55 (2C, C-7), 127.49 (1C, C-9), 78.86 (1C, C-2'), 51.58 (1C, C-1'), 49.25 (1C, C-4'), 48.67 (1C, C-11'), 46.76 (1C, C-5), 43.73 (1C, C-3'), 41.03 (1C, C-7'), 30.11 (1C, C-6'), 26.29 (1C, C-8'), 24.89 (1C, C-5'), 22.67 (1C, C-9'), 17.54 (1C, C-10').

3-(benzylamino)-4-((2-((1*R*,2*R*,4*S*)-2-hydroxy-1,3,3-trimethylbicyclo-[2.2.1]heptan-2-yl)ethyl)amino)cyclobut-3-ene-1,2-dione (7f)

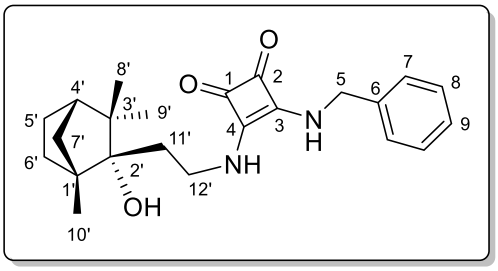

Following the general procedure, the crude product was obtained and then purified by simple filtration and washing with PE to give 0.19 g (97%) **7f** as colorless crystals.

Analytical and spectral data:

m.p. 224-225 °C. $[\alpha]_D^{20}$ = −7.1 (*c* 1.00, DMSO). MS (CI) *m/z* (rel. int.): 383 (M+1, 87), 365 (M−OH, 100), 215 (97), 173 (23), 145 (20). Anal. calcd. for $C_{23}H_{30}N_2O_3$ (382.50): C, 72.22; H, 7.91; N, 7.32. Found: C, 72.11; H, 8.03; N, 7.23 %. ^{1}H NMR (600.1 MHz, DMSO-d_6, 298 K): δ = 7.70 (br s, 1H, N<u>H</u>), 7.40-7.35 (m, 2H, H-8), 7.35-7.32 (m, 2H, H-7), 7.32-7.27 (m, 1H, H-9), 7.28-7.15 (overlapped m, 1H, N<u>H</u>Ar), 4.71 (br d, 2H, H-5, *J* = 4.0 Hz), 3.90 (s, 1H, O<u>H</u>), 3.65 (br s, 2H, H-12'), 2.03-1.96 (m, 1H, H_{endo}-6'), 1.77 (dt, 1H, H_a-11', *J* = 13.7 Hz, *J* = 7.5 Hz), 1.70 (dt, 1H, H_b-11', *J* = 13.8 Hz, *J* = 7.8 Hz), 1.63-1.54 (m, 2H, H_{endo}-5', H_{syn}-7'), 1.51 (d, 1H, H-4', *J* = 4.1 Hz), 1.32 (tt, 1H, H_{exo}-5', *J* = 12.4 Hz, *J* = 4.6 Hz), 1.01 (dd, 1H, H_{anti}-7', *J* = 10.1 Hz, *J* = 1.4 Hz), 0.99 (s, 3H, H-8'), 0.96 (s, 3H, H-10'), 0.90-0.83 (overlapped m, 1H, H_{exo}-6'), 0.88 (overlapped s, 3H, H-9'). ^{13}C NMR (150.9 MHz, DMSO-d_6, 298 K): δ = 183.05* (1C, C-1), 182.50* (1C, C-2), 168.14* (1C, C-3), 167.86* (1C, C-4), 139.45 (1C, C-6), 129.13 (2C, C-8), 128.05 (2C, C-7), 127.90 (1C, C-9), 79.74 (1C, C-2'), 52.88 (1C, C-1'), 49.95 (1C, C-4'), 47.26 (1C, C-5), 44.50 (1C, C-3'), 41.35 (1C, C-12), 41.29 (1C, C-7'), 37.90 (1C, C-11'), 30.61 (1C, C-6'), 28.09 (1C, C-8'), 25.25 (1C, C-5'), 23.06 (1C, C-9'), 18.58 (1C, C-10').

3-(benzylamino)-4-(((1*S*,2*R*,3*S*,4*R*)-3-hydroxy-4,7,7-trimethylbicyclo-[2.2.1]heptan-2-yl)amino)cyclobut-3-ene-1,2-dione (7g)

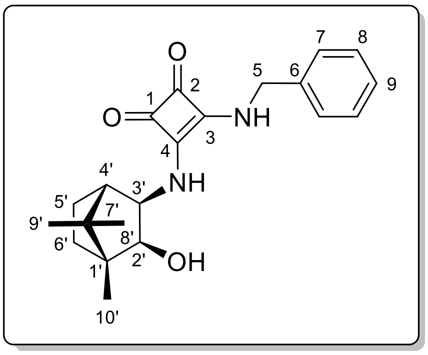

Following the general procedure, the crude product was obtained and then purified by column chromatography (30 g silica gel, eluent: a) DCM/acetone = 5:1, b) DCM/acetone = 1:1) to give 0.09 g (88%) **7g** as colorless crystals.

Analytical and spectral data:

m.p. 114 °C. $[\alpha]_D^{20}$ = +66.7 (*c* 0.60, $CHCl_3$). MS (CI) *m/z* (rel. int.): 355 (M+1, 100). Anal. calcd. for $C_{21}H_{26}N_2O_3$ (354.45): C, 71.16; H, 7.39; N, 7.90. Found: C, 71.07; H, 7.44; N, 7.97

%. ^{1}H NMR (600.1 MHz, DMSO-d$_6$, 293 K): δ = 8.28 (br t, 1H, CH$_2$NH, J = 5.3 Hz), 7.40-7.37 (m, 2H, H-8), 7.34-7.29 (m, 3H, H-7, H-9), 5.58 (d, 1H, NH, J = 5.7 Hz), 4.77 (dd, 1H, H$_a$-5, J = 14.5 Hz, J = 6.5 Hz), 4.67 (dd, 1H, H$_b$-5, J = 14.5 Hz, J = 5.7 Hz), 3.99 (t, 1H, H-2', J = 7.2 Hz), 3.66 (m, 1H, H-3'), 3.34 (s, 1H, OH), 1.71 (br d, 1H, H-4', J = 4.2 Hz), 1.65-1.58 (m, 1H, H$_{exo}$-5'), 1.44-1.38 (m, 1H, H$_{exo}$-6'), 1.10-1.01 (overlapped m, 2H, H$_{endo}$-6', H$_{endo}$-5'), 0.99 (s, 3H, H-9'), 0.84 (s, 3H, H-10'), 0.73 (s, 3H, H-8'). ^{13}C NMR (150.9 MHz, DMSO-d$_6$, 293 K): δ = 183.20* (1C, C-1), 183.11* (1C, C-2), 167.92* (1C, C-3), 167.70* (1C, C-4), 139.43 (1C, C-6), 129.23 (2C, C-8), 128.22 (2C, C-7), 127.99 (1C, C-9), 78.12 (1C, C-3'), 61.20 (1C, C-2'), 53.16 (1C, C-4'), 49.12 (1C, C-7'), 47.24 (1C, C-5), 46.52 (1C, C-1'), 33.06 (1C, C-6'), 26.11 (1C, C-5'), 22.04 (1C, C-8'), 21.57 (1C, C-9'), 12.21 (1C, C-10').

3-(benzylamino)-4-(((1*S*,2*S*)-1-hydroxy-1-phenylpropan-2-yl)amino)-cyclobut-3-ene-1,2-dione (7h)

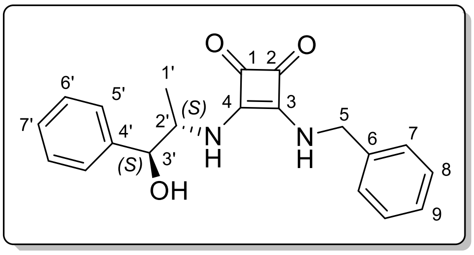

Following the general procedure, the crude product was obtained and then purified by crystallization from EtOH:H$_2$O = 2.5:0.5 ml to give 0.033 g (94%) **7h** as colorless crystals.

Analytical and spectral data:

m.p. 278 °C (with decomp.). $[\alpha]^{20}_{D}$ = +63.3 (*c* 0.30, DMSO). MS (CI) *m/z* (rel. int.): 337 (M+1, 100), 319 (M−OH, 87), 229 (M−C$_6$H$_5$CH$_2$OH, 36). Anal. calcd. for $C_{20}H_{20}N_2O_3$ (336.39): C, 71.41; H, 5.99; N, 8.33. Found: C, 71.47; H, 6.03; N, 8.38 %. ^{1}H NMR (600.1 MHz, DMSO-d$_6$, 293 K): δ = 7.87-7.78 (m, 1H, CH$_2$NH), 7.45 (br d, 1H, CHNH, J = 7.0 Hz), 7.41-7.36 (m, 2H, H-8), 7.34-7.25 (m, 7H, H-7, H-9, H-5', H-6'), 7.23-7.19 (m, 1H, H-7'), 4.67 (br d, 2H, H-5, J = 5.7 Hz), 4.63 (br s, 1H, 1H, H-3'), 4.25 (br s, 1H, H-2'), 1.25-1.15 (m, 3H, H-1'). ^{13}C NMR (150.9 MHz, DMSO-d$_6$, 293 K): δ = 182.33* (1C, C-1), 182.16* (1C, C-2), 167.79* (1C, C-3), 167.08* (1C, C-4), 142.96 (1C, C-4'), 139.03 (1C, C-6), 128.71 (2C, C-8), 127.78 (2C, C-7), 127.50 (2C, C-6'), 127.46 (1C, C-9), 127.06 (1C, C-7'), 126.42 (2C, C-5'), 74.57 (1C, C-3'), 54.97 (1C, C-2'), 46.73 (1C, C-5), 19.36 (1C, C-1').

3-(benzylamino)-4-((*S*)-2-(hydroxymethyl)pyrrolidin-1-yl)cyclobut-3-ene-1,2-dione (7i)

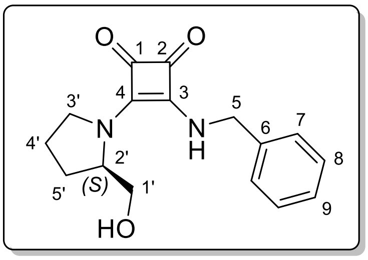

Following the general procedure the crude product was obtained and then purified by column chromatography (30 g silica gel, eluent: DCM/acetone = 2:1) to give 0.11 g (85%) **7i** as colorless crystals.

Analytical and spectral data:

m.p. 153-155 °C. $[\alpha]_D^{20}$ = −78.2 (*c* 0.95, DMSO). MS (CI) *m/z* (rel. int.): 287 (M+1, 100). Anal. calcd. for $C_{16}H_{18}N_2O_3$ (286.33): C, 67.12; H, 6.34; N, 9.78. Found: C, 67.20; H, 6.41; N, 9.86 %. ^{1}H NMR (600.1 MHz, $CDCl_3$, 323 K): δ = 7.35-7.29 (m, 4H, H-7, H-8), 7.28-7.23 (m, 1H, H-9), 4.91 (d, 1H, H_a-5, *J* = 14.5 Hz), 4.72 (d, 1H, H_b-5, *J* = 14.6 Hz), 4.15-3.98 (m, 2H, H-2', H_a-1'), 3.78-3.72 (m, 1H, H_a-3'), 3.64 (t, 1H, H_b-3', *J* = 10.1 Hz), 3.54 (dd, 1H, H_b-1', *J* = 11.5 Hz, *J* = 2.7 Hz), 2.02 (m, 1H, H_a-4'), 1.97-1.89 (m, 2H, H_a-5', H_b-4'), 1.66-1.58 (m, 1H, H_b-5'). ^{13}C NMR (150.9 MHz, DMSO-d_6, 293 K): δ = 188.67* (1C, C-1), 182.56* (1C, C-2), 167.77* (1C, C-3), 166.76* (1C, C-4), 139.94 (1C, C-6), 128.90 (2C, C-8), 127.85 (1C, C-9), 127.58 (2C, C-7), 63.91 (1C, C-1'), 61.10 (1C, C-2'), 49.18 (1C, C-3'), 46.94 (1C, C-5), 27.08 (1C, C-4'), 23.54 (1C, C-5').

3,4-bis(((*R*)-1-hydroxybutan-2-yl)amino)cyclobut-3-ene-1,2-dione (8b)

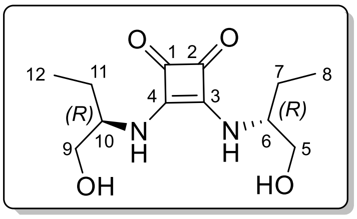

Following the general procedure, the crude product was obtained and then purified by simple filtration and washing with PE/MTBE = 10:1 to give 0.53 g (88%) **8b** as colorless crystals.

Analytical and spectral data:

m.p. 275-276 °C (with decomp.). $[\alpha]_D^{20}$ = +83.0 (*c* 1.00, DMSO). MS (CI) *m/z* (rel. int.): 257 (M+1, 100). Anal. calcd. for $C_{12}H_{20}N_2O_4$ (256.30): C, 56.24; H, 7.87; N, 10.93. Found: 56.32; H, 7.81; N, 10.96 %. ^{1}H NMR (600.1 MHz, DMSO-d_6, 293 K): δ = 7.40 (br d, 2H, N_H_, *J* = 7.1 Hz), 4.95 (m, 2H, O_H_), 3.85 (m, 2H, H-6, H-10), 3.58-3.32 (m, overlapped with water peak, 4H, H-5, H-9), 1.68-1.52 (m, 2H, H_a-7, H_a-11), 1.51-1.34 (m, 2H, H_b-7, H_b-11), 0.88 (t, 6H, H-8, H-12, *J* = 7.4 Hz). ^{13}C NMR (150.9 MHz, DMSO-d_6, 293 K): δ = 182.12 (2C, C-1, C-2), 167.87 (2C, C-3, C-4), 63.29 (2C, C-5, C-9), 56.88 (2C, C-6, C-10), 25.04 (2C, C-7, C-11), 10.32 (2C, C-8, C-12).

3-((((1*R*,2*R*,4*S*)-2-hydroxy-1,3,3-trimethylbicyclo[2.2.1]heptan-2-yl)methyl)amino)-4-(((*R*)-1-hydroxybutan-2-yl)amino)cyclobut-3-ene-1,2-dione (8e)

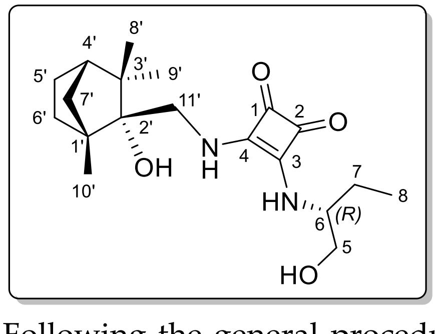

Following the general procedure, the crude product was obtained and then purified by column chromatography (30 g silica gel, eluent: a) DCM/acetone = 5:1, b) DCM/acetone = 1:1) to give 0.10 g (83%) **8e** as colorless crystals.

Analytical and spectral data:

m.p. 225-227 °C. $[\alpha]_D^{20}$ = +34.7 (*c* 1.00, DMSO). MS (CI) *m/z* (rel. int.): 351 (M+1, 100), 333 (M−OH, 51), 198 (M−fenchol, 17). Anal. calcd. for $C_{19}H_{30}N_2O_4$ (350.46): C, 65.12; H, 8.63; N, 7.99. Found: C, 65.22; H, 8.51; N, 8.10 %. ^{1}H NMR (600.1 MHz, DMSO-d_6, 293 K): δ = 7.79 (d, 1H, CHN<u>H</u>, *J* = 9.0 Hz), 7.41-7.29 (m, 1H, CH_2N<u>H</u>), 4.91 (t, 1H, CH_2O<u>H</u>, *J* = 5.2 Hz), 4.26 (s, 1H, O<u>H</u>), 3.92-3.83 (m, 2H, H_a-11', H-6), 3.49(dd, 1H, H_b-11', *J* = 13.3 Hz, *J* = 3.6 Hz), 3.45 (t, 2H, H-5, *J* = 5.0 Hz), 2.06-1.97 (m, 1H, H_{endo} -6'), 1.67-1.56 (m, 3H, H_{endo}-5', H_a-7, H_{syn}-7'), 1.54 (br d, 1H, H-4', *J* = 4.0 Hz), 1.47-1.40(m, 1H, H_b-7), 1.36 (tt, 1H, H_{exo}-5', *J* = 12.3 Hz, *J* = 4.7 Hz), 1.09 (dd, 1H, H_{anti}-7', *J* = 10.1 Hz, *J* = 1.1 Hz), 0.98 (s, 3H, H-8'), 0.97 (s, 3H, H-10'), 0.92 (s, 3H, H-9'), 0.91-0.87 (overlapped m, 1H, H_{exo}-6'), 0.85 (overlapped t, 3H, H-8, *J* = 7.4 Hz). ^{13}C NMR (150.9 MHz, DMSO-d_6, 293 K): δ = 182.74* (1C, C-1), 181.94* (1C, C-2), 168.12* (1C, C-3), 167.46* (1C, C-4), 78.87 (1C, C-2'), 63.32 (1C, C-5), 56.61 (1C, C-6), 51.59 (1C, C-1'), 49.29 (1C, C-4'), 48.67 (1C, C-11'), 43.74 (1C, C-3'), 41.05 (1C, C-7'), 30.12 (1C, C-6'), 26.31 (1C, C-8'), 24.98 (1C, C-7), 24.91 (1C, C-5'), 22.68 (1C, C-9'), 17.56 (1C, C-10'), 10.17 (1C, C-8).

3-((2-((1*R*,2*R*,4*S*)-2-hydroxy-1,3,3-trimethylbicyclo[2.2.1]heptan-2-yl)ethyl)amino)-4-(((*R*)-1-hydroxybutan-2-yl)amino)cyclobut-3-ene-1,2-dione (8f)

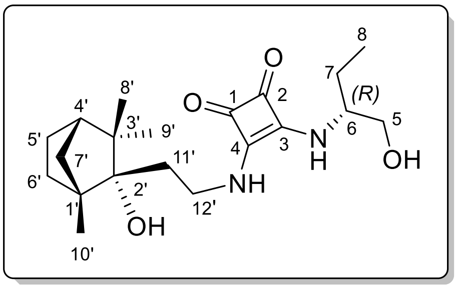

Following the general procedure the crude product was obtained and then purified by simple filtration and washing with PE to give 0.18 g (99%) **8f** as colorless crystals.

Analytical and spectral data:

m.p. 199-200 °C. $[\alpha]_D^{20}$ = +7.6 (*c* 0.81, DMSO). MS (CI) *m/z* (rel. int.): 365 (M+1, 100), 347 (M−OH, 73), 197 (81). Anal. calcd. for $C_{20}H_{32}N_2O_4$ (364.49): C, 65.91; H, 8.85; N, 7.69. Found: C, 65.83; H, 8.93; N, 7.76 %. ^{1}H NMR (600.1 MHz, DMSO-d_6, 293 K): 7.42-7.23 (m, 2H, N<u>H</u>), 4.92 (m, 1H, O<u>H</u>), 3.95 (s, 1H, O<u>H</u>), 3.86 (br s, 1H, H-6), 3.66 (br s, 2H, H-12), 3.50-3.42 (m, 2H, H-5), 2.04-1.96 (m, 1H, H_{endo}-6'), 1.81-1.74 (m, 1H, H_a-11'), 1.74-1.67 (m, 1H, H_b-11'), 1.64-1.54 (m, 3H, H_a-7, H_{endo}-5', H_{syn}-7'), 1.52(br d, 1H, H-4', *J* = 4.1 Hz), 1.44 (dt, 1H, H_b-7, *J* = 14.8 Hz, *J* = 7.6 Hz), 1.33 (overlapped tt, 1H, H_{exo}-5', *J* = 12.5 Hz, *J* = 4.6 Hz), 1.02 (dd, 1H, H_{anti}-7', *J* = 10.1 Hz, *J* = 1.5 Hz), 1.01 (s, 3H, H-8'), 0.98 (s, 3H, H-10'), 0.88 (s, 3H, H-9'), 0.89-0.83 (overlapped m, 1H, H_{exo}-6'), 0.87 (overlapped t, 3H, H-8, *J* = 7.5 Hz). ^{13}C NMR (150.9 MHz, DMSO-d_6, 293 K): δ = 182.72* (1C, C-1), 182.18* (1C, C-2), 168.34* (1C, C-3), 167.86* (1C, C-4), 79.69 (1C, C-2'), 63.73 (1C, C-5), 57.27 (1C, C-6), 52.92 (1C, C-1'), 49.99 (1C, C-4'), 44.53 (1C, C-3'), 41.32 (2C, C-12, C-7'), 38.00 (1C, C-11'), 30.66 (1C, C-6'), 28.13 (1C, C-8'), 25.31 (2C, C-7, C-5'), 23.12 (1C, C-9'), 18.65 (1C, C-10'), 10.74 (1C, C-8).

3-(((*R*)-1-hydroxybutan-2-yl)amino)-4-(((*S*)-1-hydroxybutan-2-yl)amino)cyclobut-3-ene-1,2-dione (9b)

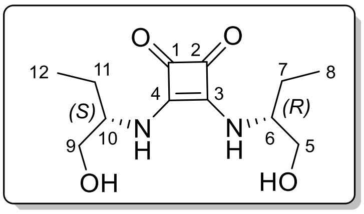

Following the general procedure, the crude product was obtained and then purified by simple filtration and washing with PE/MTBE = 10:1 to give 0.60 g (99%) **9b** as colorless crystals.

Analytical and spectral data:

m.p. 197-198 °C. $[\alpha]_D^{20}$ = 0 (*c* 1.03, DMSO). MS (CI) *m/z* (rel. int.): 257 (M+1, 100). Anal. calcd. for $C_{12}H_{20}N_2O_4$ (256.30): C, 56.24; H, 7.87; N, 10.93. Found: C, 56.20; H, 7.94; N, 10.91 %. ^{1}H NMR (600.1 MHz, DMSO-d_6, 293 K): δ = 7.42 (br d, 2H, NH, *J* = 7.7 Hz), 4.97 (m, 2H, OH), 3.85 (m, 2H, H-6, H-10), 3.56-3.32 (m, overlapped with water peak, 4H, H-5, H-9), 1.68-1.52 (m, 2H, H_a-7, H_a-11), 1.51-1.33 (m, 2H, H_b-7, H_b-11), 0.86 (t, 6H, H-8, H-12, *J* = 7.4 Hz). ^{13}C NMR (150.9 MHz, DMSO-d_6, 293 K): δ = 182.11 (2C, C-1, C-2), 167.85 (2C, C-3, C-4), 63.35 (2C, C-5, C-9), 56.85 (2C, C-6, C-10), 25.00 (2C, C-7, C-11), 10.32 (2C, C-8, C-12).

3,4-bis(((*S*)-1-hydroxybutan-2-yl)amino)cyclobut-3-ene-1,2-dione (9c)

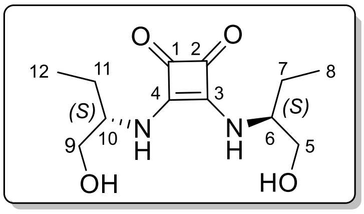

Following the general procedure, the crude product was obtained and then purified by simple filtration and washing with PE/MTBE = 10:1 to give 0.57 g (95%) **9c** as colorless crystals.

Analytical and spectral data:

m.p. 275-276 °C (with decomp.). $[\alpha]_D^{20}$ = −80.0 (*c* 1.00, DMSO). MS (CI) *m/z* (rel. int.): 257 (M+1, 100). Anal. calcd. for $C_{12}H_{20}N_2O_4$ (256.30): C, 56.24; H, 7.87; N, 10.93. Found: C, 56.27; H, 7.93; N, 10.98 %. ^{1}H NMR (600.1 MHz, DMSO-d_6, 293 K): δ = 7.40 (br d, 2H, NH, *J* = 7.7 Hz), 4.95 (m, 2H, OH), 3.85 (m, 2H, H-6, H-10), 3.49-3.40 (m, overlapped with water peak, 4H, H-5, H-9), 1.66-1.52 (m, 2H, H_a-7, H_a-11), 1.49-1.37 (m, 2H, H_b-7, H_b-11), 0.87 (t, 6H, H-8, H-12, *J* = 7.4 Hz). ^{13}C NMR (150.9 MHz, DMSO-d_6, 293 K): δ = 182.12 (2C, C-1, C-2), 167.87 (2C, C-3, C-4), 63.29 (2C, C-5, C-9), 56.88 (2C, C-6, C-10), 25.04 (2C, C-7, C-11), 10.32 (2C, C-8, C-12).

3-((((1*R*,2*R*,4*S*)-2-hydroxy-1,3,3-trimethylbicyclo[2.2.1]heptan-2-yl)methyl)amino)-4-(((*S*)-1-hydroxybutan-2-yl)amino)cyclobut-3-ene-1,2-dione (9e)

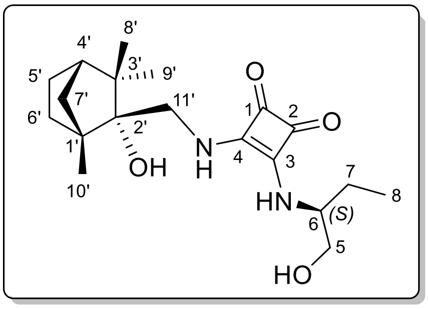

Following the general procedure, the crude product was obtained and then purified by column chromatography (30 g silica gel, eluent: DCM/acetone = 1:1) to give 0.096 g (91%) **9e** as colorless crystals.

<u>*Analytical and spectral data:*</u>
m.p. 235-236 °C. $[\alpha]_D^{20}$ = −14.7 (*c* 0.72, DMSO). MS (CI) *m/z* (rel. int.): 351 (M+1, 100), 333 (M−OH, 55), 198 (M−fenchol, 18). Anal. calcd. for $C_{19}H_{30}N_2O_4$ (350.46): C, 65.12; H, 8.63; N, 7.99. Found: C, 65.17; H, 8.58; N, 7.98 %. ^{1}H NMR (600.1 MHz, DMSO-d$_6$, 293 K): δ = 7.78 (d, 1H, CHN<u>H</u>, *J* = 8.9 Hz), 7.38-7.31 (m, 1H, N<u>H</u>), 4.92 (t, 1H, CH$_2$O<u>H</u>, *J* = 5.1 Hz), 4.25 (s, 1H, O<u>H</u>), 3.92-3.84 (m, 2H, H$_a$-11', H-6), 3.49(dd, 1H, H$_b$-11', *J* = 13.4 Hz, *J* = 3.5 Hz), 3.45 (t, 2H, H-5, *J* = 4.9 Hz), 2.05-1.98 (m, 1H, H$_{endo}$-6'), 1.66-1.56 (m, 3H, H$_{endo}$-5', H$_a$-7, H$_{syn}$-7'), 1.55 (br d, 1H, H-4', *J* = 3.8 Hz), 1.43(dt, 1H, H$_b$-7, *J* = 14.2 Hz, *J* = 7.6 Hz), 1.36 (tt, 1H, H$_{exo}$-5', *J* = 12.4 Hz, *J* = 4.7 Hz), 1.09 (d, 1H, H$_{anti}$-7', *J* = 9.8 Hz), 0.98 (s, 6H, H-8', H-10'), 0.92 (s, 3H, H-9'), 0.91-0.87 (overlapped m, 1H, H$_{exo}$-6'), 0.85 (overlapped t, 3H, H-8, *J* = 7.4 Hz). ^{13}C NMR (150.9 MHz, DMSO-d$_6$, 293 K): δ = 182.74* (1C, C-1), 181.96* (1C, C-2), 168.09* (1C, C-3), 167.46* (1C, C-4), 78.83 (1C, C-2'), 63.30 (1C, C-5), 56.60 (1C, C-6), 51.59 (1C, C-1'), 49.25 (1C, C-4'), 48.61 (1C, C-11'), 43.71 (1C, C-3'), 41.03 (1C, C-7'), 30.13 (1C, C-6'), 26.32 (1C, C-8'), 25.00 (1C, C-7), 24.92 (1C, C-5'), 22.73 (1C, C-9'), 17.52 (1C, C-10'), 10.17 (1C, C-8).

3-(((*S*)-3,3-dimethylbutan-2-yl)amino)-4-((1-hydroxy-2-methylpropan-2-yl)amino)cyclobut-3-ene-1,2-dione (10)

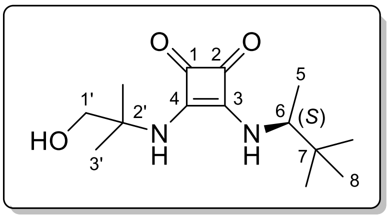

Following the general procedure, the crude product was obtained and then purified by column chromatography (30 g silica gel, eluent: a) DCM/acetone = 5:1, b) DCM/acetone = 3:1) to give 0.12 g (81%) **10** as colorless crystals.

<u>*Analytical and spectral data:*</u>
m.p. 276-278 °C. $[\alpha]_D^{20}$ = −1.3 (*c* 0.96, DMSO). MS (CI) *m/z* (rel. int.): 269 (M+1, 100), 197 (M−*i*-BuOH, 15). Anal. calcd. for $C_{14}H_{24}N_2O_3$ (268.36): C, 62.66; H, 9.01; N, 10.44. Found: C, 62.59; H, 8.95; N, 10.40 %. ^{1}H NMR (600.1 MHz, DMSO-d$_6$, 293 K): δ = 7.49 (s, 1H, N<u>H</u>C(CH$_3$)$_2$), 7.48 (s, 1H, N<u>H</u>CH), 5.15 (t, 1H, O<u>H</u>, *J* = 5.6 Hz), 3.91 (qd, 1H, H-6, *J* = 9.9 Hz,

J = 6.8 Hz), 3.42 (d, 2H, H-1', J = 5.4 Hz), 1.28 (d, 6H, H-3', J = 1.4 Hz), 1.10 (d, 3H, H-5, J = 6.8 Hz), 0.86 (s, 9H, H-8). ^{13}C NMR (150.9 MHz, DMSO-d$_6$, 293 K): δ = 182.21* (1C, C-1), 180.92* (1C, C-2), 168.55* (1C, C-3), 167.71* (1C, C-4), 68.96 (1C, C-1'), 58.10 (1C, C-6), 56.21 (1C, C-2'), 34.81 (1C, C-7), 25.91 (3C, C-8), 25.48 (2C, C-3'), 16.97 (1C, C-5).

Preparation of 12a and 12b

General procedure:

A solution of 3,4-diethoxycyclobut-3-ene-1,2-dione **1** (1.0 equiv) and Et_3N (1.1 equiv) in 15 ml EtOH was cooled to 0 °C. Then the requisite amine (1.1 equiv) was added and the resulting mixture was stirred at room temperature for 24 h. The solvent was removed under reduced pressure and the crude product was purified by column chromatography.

(*S*)-3-etoxy-4-((1-phenylethyl)amino)cyclobut-3-ene-1,2-dione (12a)

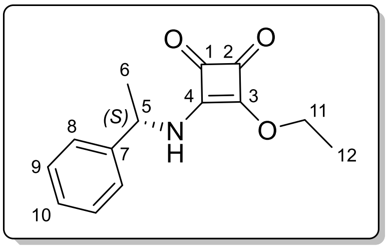

Following the general procedure, 3,4-diethoxycyclobut-3-ene-1,2-dione **1** (1.08 mmol, 0.16 ml), Et_3N (1.19 mmol, 0.17 ml) and (*S*)-1-phenylethylamine **11a** (1.19 mmol, 0.15 ml) were stirred for 24 h. The crude product was purified by column chromatography (60 g silica gel, eluent: DCM/MTBE = 20:1) to give 0.26 g (99%) **12a** as pale yellow oil.

Analytical and spectral data:

$[\alpha]_D^{20}$ = −53.1 (*c* 1.11, $CHCl_3$). MS (ESI) *m/z* (rel. int.): 246 (100, M+1), 142 (M−CH(CH_3)Ph), 114 (92, M−CH_2CH_3, CH(CH_3)Ph). Anal. calcd. for $C_{14}H_{15}NO_3$ (245.28): C, 68.56; H, 6.16; N, 5.71. Found: C, 68.60; H, 6.24; N, 5.75 %. ^{1}H NMR (600.1 MHz, $CDCl_3$, 298 K): δ = 7.39-7.34 (m, 2H, H-9), 7.34-7.27 (m, 3H, H-8, H-10), 6.71 (br s, 1H, N<u>H</u>), 4.94-4.83 (m, 1H, H-5), 4.73 (q, 2H, H-11, J = 7.1 Hz). 1.64 (d, 3H, H-6, J = 6.9 Hz), 1.43-1.37 (m, 1H, H-12). ^{13}C NMR (150.9 MHz, $CDCl_3$, 298 K): δ = 183.20* (1C, C-1), 177.67* (1C, C-2), 177.39* (1C, C-3), 171.57* (1C, C-4), 142.21 (1C, C-7), 128.96 (2C, C-9), 128.07 (1C, C-10), 125.93 (2C, C-8), 69.65 (1C, C-11), 54.70 (1C, C-5), 22.90 (1C, C-6), 15.71 (1C, C-12).

3-ethoxy-4-(((1*R*,2*R*,4*R*)-1,7,7-trimethylbicyclo[2.2.1]heptan-2-yl)amino)cyclobut-3-ene-1,2-dione (12b)

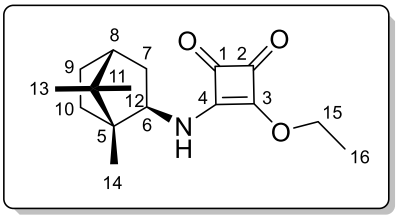

Following the general procedure, 3,4-diethoxycyclobut-3-ene-1,2-dione **1** (1.08 mmol, 0.16 ml), Et_3N (1.19 mmol, 0.17 ml) and bornylamine **11b** (*exo*:*endo* = 88:12) (1.08 mmol, 0.17 g)

were stirred for 24 h. The crude product was purified by column chromatography (60 g silica gel, eluent: DCM/CH/*i*-PrOH = 5:1:0.025) to give 0.20 g (66%) **12b** as colorless crystals.

<u>*Analytical and spectral data:*</u>
m.p. 134-135 °C. $[\alpha]_D^{20}$ = +58.5 (*c* 0.83, $CHCl_3$). MS (CI) *m/z* (rel. int.): 278 (M+1, 100), 142 (M−bornane, 33), 137 (bornane−1, 16). Anal. calcd. for $C_{16}H_{23}NO_3$ (277.36): C, 69.29; H, 8.36; N, 5.05. Found: C, 69.16; H, 8.26; N, 5.10 %. ^{1}H NMR (600.1 MHz, $CDCl_3$, 298 K): δ = 5.65 (br d, 1H, N<u>H</u>, *J* = 8.6 Hz), 4.84-4.68 (m, 2H, H-15), 3.60 (td, 1H, H-6), 1.93-1.87 (m, 1H, H_{exo}-7), 1.83-1.79 (m, 1H, H-8), 1.79-1.68 (m, 2H, H_{endo}-7, H_{exo}-9), 1.65-1.59 (m, 1H, H_{exo}-10), 1.50-1.46 (t, 3H, H-16, *J* = 7.1 Hz), 1.19-1.10 (m, 2H, H_{endo}-9, H_{endo}-10), 0.92 (s, 3H, H-13), 0.86 (s, 6H, H-12, H-14). ^{13}C NMR (150.9 MHz, $CDCl_3$, 298 K): δ = 188.99* (1C, C-1), 183.04* (1C, C-2), 176.74* (1C, C-3), 172.02* (1C, C-4), 69.57 (1C, C-15), 63.20 (1C, C-6), 49.80 (1C, C-11), 47.02 (1C, C-5), 44.70 (1C, C-8), 39.00 (1C, C-7), 35.49 (1C, C-10), 26.81 (1C, C-9), 20.25 (1C, C-13), 15.88 (1C, C-16), 11.61 (1C, C-14, C-12).

Preparation of 13a and 13b

A solution of **12a** or **12b** (1 equiv.) in 2 ml THF was treated with 10 ml 10% aqueous HCl. The resulting mixture was stirred at room temperature for 24 h. The residue was filtered, washed with water and evaporated *in vacuo*.

(*S*)-3-hydroxy-4-((1-phenylethyl)amino)cyclobut-3-ene-1,2-dione (13a)

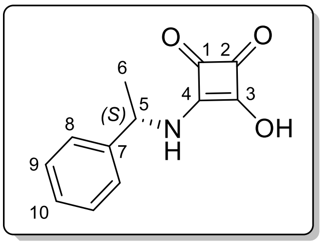

Following the general procedure, 0.20 g (85%) **13a** were obtained as **c**olorless powder.

<u>*Analytical and spectral data:*</u>
m.p. 202-203 °C. $[\alpha]_D^{20}$ = −200.6 (*c* 0.99, acetone). MS (CI) *m/z* (rel. int.): 217 (M+1, 100), 142 (M−phenyl, 36), 114 (M−ethylbenzene, 98), 105 (ethylbenzene−1, 92). Anal. calcd. for $C_{12}H_{11}NO_3$ (217.22): C, 66.35; H, 5.10; N, 6.45. Found: C, 66.27; H, 5.20; N, 6.40 %. ^{1}H NMR (600.1 MHz, DMSO-d_6, 338 K): δ = 10.25 (br s, 1H, O<u>H</u>), 8.65 (br d, 1H, NH, *J* = 7.0 Hz), 7.39-7.32 (m, 4H, H-8, H-9), 7.29-7.24 (m, 1H, H-10), 5.14-5.06 (m, 1H, H-5), 1.53 (d, 3H, H-6, *J* = 6.9 Hz). ^{13}C NMR (150.9 MHz, DMSO-d_6, 338 K): δ = 185.03* (1C, C-1), 184.29* (1C, C-2), 173.38* (1C, C-3 or C-4), 143.93 (1C, C-7), 128.63 (2C, C-9), 127.30 (1C, C-10), 126.09 (2C, C-8), 53.42 (1C, C-5), 22.62 (1C, C-6).

3-hydroxy-4-(((1*R*,2*R*,4*R*)-1,7,7-trimethylbicyclo[2.2.1]heptan-2-yl)amino)cyclobut-3-ene-1,2-dione (13b)

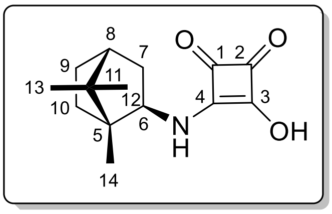

Following the general procedure, 0.16 g (90%) **13a** were obtained as colorless powder.

Analytical and spectral data:

m.p. 220 °C (with decomp.). $[\alpha]_D^{20}$ = +15.9 (*c* 1.00, DMSO). MS (CI) *m/z* (rel. int.): 250 (M+1, 58), 137 (bornane−1, 100), 114 (M−bornane, 42), 81 (M−bornylamine, 38). Anal. calcd. for $C_{14}H_{19}NO_3$ (249.31): C, 67.45; H, 7.68; N, 5.62. Found: C, 67.34; H, 7.57; N, 5.54 %. ^{1}H NMR (600.1 MHz, DMSO-d$_6$, 293 K): δ = 7.94 (br d, 1H, NH, *J* = 7.7 Hz), 3.81-3.70 (m, 1H, H-6), 1.88-1.80 (m, 1H, H_{exo}-7), 1.79-1.71 (m, 1H, H_{endo}-7), 1.71-1.68 (m, 1H, H-8), 1.68-1.61 (m, 1H, H_{exo}-9), 1.53-1.46 (m, 1H, H_{exo}-10), 1.13-1.05 (m, 2H, H_{endo}-9, H_{endo}-10), 0.91 (s, 3H, H-13), 0.79 (s, 3H, H-12), 0.77 (s, 3H, H-14). ^{13}C NMR (150.9 MHz, DMSO-d$_6$, 293 K): δ = 185.14* (1C, C-1 or C-2), 174.54* (1C, C-3 or C-4), 62.22 (1C, C-6), 50.30 (1C, C-11), 46.92 (1C, C-5), 44.65 (1C, C-8), 37.18 (1C, C-7), 35.50 (1C, C-10), 27.08 (1C, C-9), 20.99 (1C, C-12), 20.29 (1C, C-13), 11.68 (1C, C-14).

Preparation of aminoalcohol 17

A solution of amino acid **14** (1.94 mmol, 0.20 g) in 5 ml H_2O was treated with K_2CO_3 (5.82 mmol, 0.81 g) and BnBr (5.82 mmol, 0.69 ml). The reaction mixture was heated at reflux for 3 h then allowed to cool to room temperature and extracted with Et_2O. The combined organic extracts were washed with brine, then dried and concentrated *in vacuo*. The crude product was purified by column chromatography (36 g silica gel; eluent: PE/MTBE = 100:1) to give 0.62 g (86%) of **15** as pale yellow oil. The obtained product (1.31 mmol, 0.49 g) was dissolved under inert atmosphere in 4 ml dry THF and then treated with PhMgCl (2M solution in THF) (5.26 mmol, 2.63 ml) at 0 °C. The resulting mixture was stirred for 5 min at 0 °C and 4 h at room temperature until **15** was consumed (TLC: PE/MTBE = 25:1). After quenching with aqueous NH_4Cl, the mixture was filtered through Celite® and extracted twice with ether. The combined organic extracts were dried with $MgSO_4$ and concentrated *in vacuo*. The crude product was purified by column chromatography (28 g silica gel; eluent: PE/MTBE = 30:1) to give 0.48 g (86%) of **16** as colorless crystals. Then **16** (0.83 mmol, 0.35 g) was dissolved in 10 ml MeOH with 4.4% HCOOH and the solution was degassed with argon. Pd/C (0.33 mmol, 0.35 g) was added and the reaction was monitored by TLC (MTBE) – reaction time 2h. The residue was filtered through Celite®, washed with DCM and concentrated *in vacuo*. The crude product was purified by column chromatography (14 g silica gel, eluent: PE/MTBE = 1:2) to give 0.17g (86%) **17** as off-white crystals.

(*S*)-2-(dibenzylamino)butanoate (15)

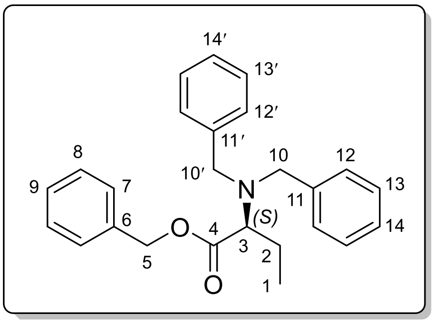

Analytical and spectral data:

$[\alpha]_D^{20}$ = +11.7 (*c* 1.37, $CHCl_3$). MS (ESI) *m*/*z* (rel. int.): 374 (100, M+1), 282 (39, M–Ph). Anal. calcd. for $C_{30}H_{31}NO$ (373.50): C, 80.40; H, 7.29; N, 3.75. Found: C, 80.35; H, 7.30; N, 3.82 %.^{1}H NMR (600.1 MHz, $CDCl_3$, 300 K): δ = 7.43-7.37 (m, 4H, H-7, H-8), 7.37-7.33 (m, 1H, H-9), 7.33-7.30* (m, 4H, H-12, H-13), 7.30-7.26* (m, 4H, H-12', H-13'), 7.24-7.19 (m, 2H, H-14, H-14'), 5.25 (d, 1H, H_a-5, *J* = 12.3 Hz), 5.15 (d, 1H, H_b-5, *J* = 12.3 Hz), 3.91 (d, 2H, H-10, *J* = 14.0 Hz), 3.51* (d, 2H, H-10, *J* = 14.0 Hz), 3.27 (t, 1H, H-3, *J* = 7.6 Hz), 1.83-1.71 (m, 2H, H-2), 0.90 (t, 3H, H-1, *J* = 7.4 Hz). ^{13}C NMR (150.9 MHz, $CDCl_3$, 300 K): δ = 172.91 (1C, C-4), 139.72 (2C, C-11), 136.19 (1C, C-6), 128.79* (4C, C-12, C-13), 128.59* (2C, C-7), 128.44* (2C, C-8), 128.29 (1C, C-9), 128.20* (4C, C-12, C-13, C-12', C-13'), 126.91 (2C, C-14, C-14'), 65.86 (1C, C-5), 62.59 (1C, C-3), 54.46 (2C, C-10), 22.70 (1C, C-2), 11.00 (1C, C-1).

(*S*)-2-(dibenzylamino)-1,1-diphenylbutan-1-ol (16)

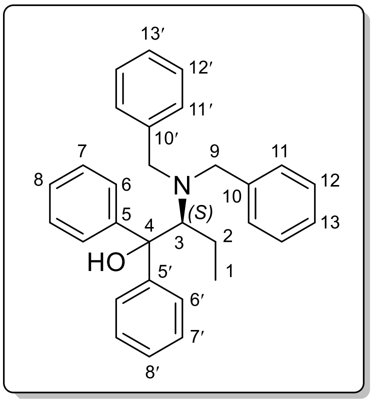

Analytical and spectral data:

m.p. 110-112 °C. $[\alpha]_D^{20}$ = –57.2 (*c* 1.03, $CHCl_3$). MS (CI) *m*/*z* (rel. int.): 238 (M+1, 100). Anal. calcd. for $C_{30}H_{31}NO$ (421.58): C, 85.47; H, 7.41; N, 3.32. Found: C, 85.42; H, 7.50; N, 3.40 %. ^{1}H NMR (600.1 MHz, $CDCl_3$, 293 K): δ = 7.61-7.58* (m, 2H, H-6), 7.34-7.30* (m, 4H, H-11, H-12), 7.29-7.22* (m, 8H, H-7, H-7', H-11', H-12'), 7.21-7.14 (m, 4H, H-8, H-8', H-13), 7.12-7.08* (m, 2H, H-6'), 5.33 (br s, 1H, O<u>H</u>), 3.66 (br s, 1H, H-3), 3.61-3.43 (m, 4H, H-9), 1.97-1.87 (m, 1H, H_a-2), 1.87-1.76 (m, 1H, H_b-2), 1.06 (t, 3H, H-1, *J* = 7.4 Hz). ^{13}C NMR (150.9 MHz, $CDCl_3$, 293 K): δ = 146.22 (1C, C-5), 144.08 (1C, C-5'), 139.25 (2C, C-10, C-10'), 129.46* (4C, C-11, C-12), 128.51 (4C, C-11', C-12'), 128.01 (2C, C-13, C-13'), 127.83* (2C, C-6), 127.51* (2C, C-7), 127.34* (4C, C-6', C-7'), 126.85 (2C, C-8, C-8').

(*S*)-2-amino-1,1-diphenylbutan-1-ol (17)

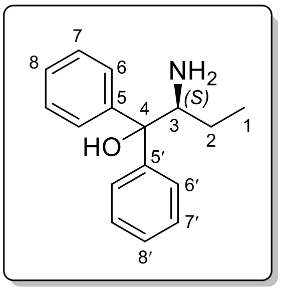

Analytical and spectral data:

m.p. 84-85 °C. $[\alpha]_D^{20}$ = −73.5 (*c* 1.10, $CHCl_3$). MS (CI) *m*/*z* (rel. int.): 224 (M−OH, 60), 58 (M−benzophenone, 100). Anal. calcd. for $C_{16}H_{19}NO$ (241.33): C, 79.63; H, 7.94; N, 5.80. Found: C, 79.75; H, 7.85; N, 5.71 %. ^1H NMR (600.1 MHz, $CDCl_3$, 293 K): δ = 7.62-7.58* (m, 2H, H-6), 7.50-7.45* (m, 2H, H-6'), 7.34 -7.30* (m, 2H, H-7), 7.29-7.25* (m, 2H, H-7'), 7.21-7.18* (m, 1H, H-8), 7.17-7.14* (m, 1H, H-8'), 3.79 (dd, 1H, H-3, *J* = 10.0 Hz, *J* = 2.3 Hz), 1.42-1.34 (m, 1H, H_a-2), 1.28-1.20 (m, 1H, H_b-2), 0.92 (t, 3H, H-1, *J* = 7.5 Hz). ^{13}C NMR (150.9 MHz, $CDCl_3$, 293 K): δ = 147.03* (1C, C-5), 144.57* (1C, C-5'), 128.45* (2C, C-7), 128.04* (2C, C-7'), 126.70* (1C, C-8), 126.38* (1C, C-8'), 125.94* (2C, C-6), 125.62* (2C, C-6'), 79.00 (1C, C-4), 58.54 (1C, C-3), 23.37 (1C, C-2), 11.61 (1C, C-1).

Preparation of squaric monoamide 18

A solution of 3,4-diethoxycyclobut-3-ene-1,2-dione **1** (0.29 mmol, 0.04 ml) and Et_3N (0.32 mmol, 0.04 ml) in 5 ml EtOH was cooled to 0 °C. Then **17** (0.29 mmol, 0.07 g) was added and the resulting mixture was stirred at room temperature for 24 h. The solvent was removed under reduced pressure and the crude product was purified by flash chromatography (5 g silica gel, eluent: DCM/acetone = 50:1) to give 0.10 g (97%) **18** as colorless crystals.

(*S*)-3-ethoxy-4-((1-hydroxy-1,1-diphenylbutan-2-yl)amino)cyclobut-3-ene-1,2-dione (18)

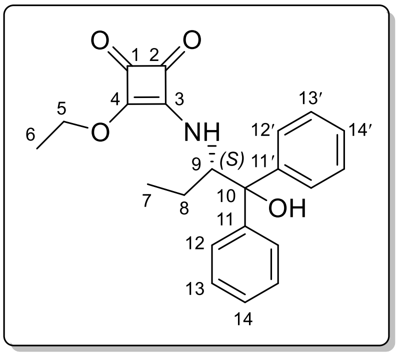

Analytical and spectral data:

m.p. 88-89 °C (with decomp.). $[\alpha]_D^{20}$ = −36.9 (*c* 0.52, $CHCl_3$). MS (CI) *m*/*z* (rel. int.): 366 (M+1, 50), 348 (M−OH, 100), 183 (benzophenone+1, 72). Anal. calcd. for $C_{22}H_{23}NO_4$ (365.43): C, 72.31; H, 6.34; N, 3.83. Found: C, 72.40; H, 6.27; N, 3.80 %. ^1H NMR (600.1 MHz, CD_3CN, 348 K): δ = 7.56-7.52* (m, 2H, H-12), 7.51-7.47* (m, 2H, H-12'), 7.41-7.36* (m,

2H, H-13'), 7.31-7.24* (m, 3H, H-13, H-14), 7.20-7.15* (m, 1H, H-14'), 6.27 (br s, 1H, N<u>H</u>), 5.08 (br s, 1H, O<u>H</u>), 4.67 (br s, 2H, H-5), 3.90 (s, 1H, H-9), 1.67-1.56 (m, 2H, H-8), 1.45 (m, 3H, H-6), 0.98 (t, 3H, H-7, J = 7.4 Hz). ^{13}C NMR (150.9 MHz, $CDCl_3$, 293 K): δ = 189.96* (1C, C-1), 181.56* (1C, C-2), 177.17* (1C, C-3), 171.93* (1C, C-4), 144.33* (1C, C-11), 143.72* (1C, C-11'), 128.74* (2C, C-13), 128.34* (2C, C-13'), 127.46* (1C, C-14), 127.37* (1C, C-14'), 125.44* (1C, C-12), 125.31* (1C, C-12'), 81.24 (1C, C-10), 70.04 (1C, C-5), 64.04 (1C, C-9), 23.88 (1C, C-8), 16.07 (1C, C-6), 11.06 (1C, C-7).

General procedure for the catalytic reduction of prochiral ketones

BH_3•SMe_2 was added to a solution of chiral ligand in dry THF (or toluene) under argon atmosphere at 0 °C. The reaction mixture was stirred for 30 min at that temperature and for another 2 h at room temperature. Finally the mixture was heated to 50 °C and stirred for additional 1 hour. The ketone was added slowly over a period of 30 min and the reaction was monitored by TLC (PE/EtOAc = 10:1). When the reaction was completed the mixture was allowed to cool to 0°C and quenched with 1N HCl. The organic layer was extracted with EtOAc and washed with brine. The combined organic extracts were dried with $MgSO_4$ and concentrated *in vacuo*. The residue was filtered through short silica gel column (eluent: PE/EtOAc = 10:1) before being subjected to GC analysis.

Plausible transition states involving ligand 2c

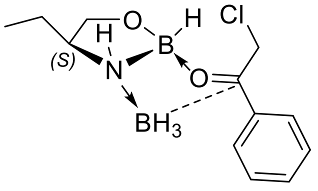

favoured BH_3-adduct with (S)-2-aminobutan-1-ol

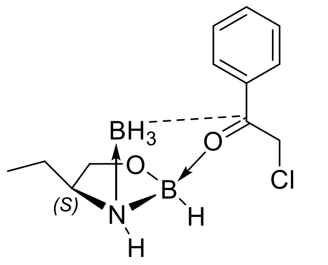

disfavoured BH_3-adduct with (S)-2-aminobutan-1-ol

HRMS analysis of intermediates formation (ESI, positive mode)

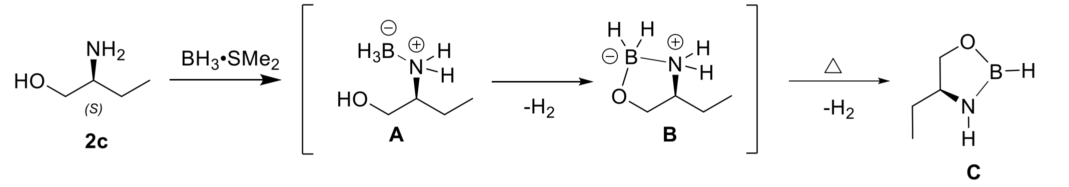

$[M+H]^+$: 90.09134
90.094560

$[M+H]^+$: 101.11210
102.10847
103.11183

$[M+H]^+$: 99.096545
100.09282
101.09652

Calculated MS spectra

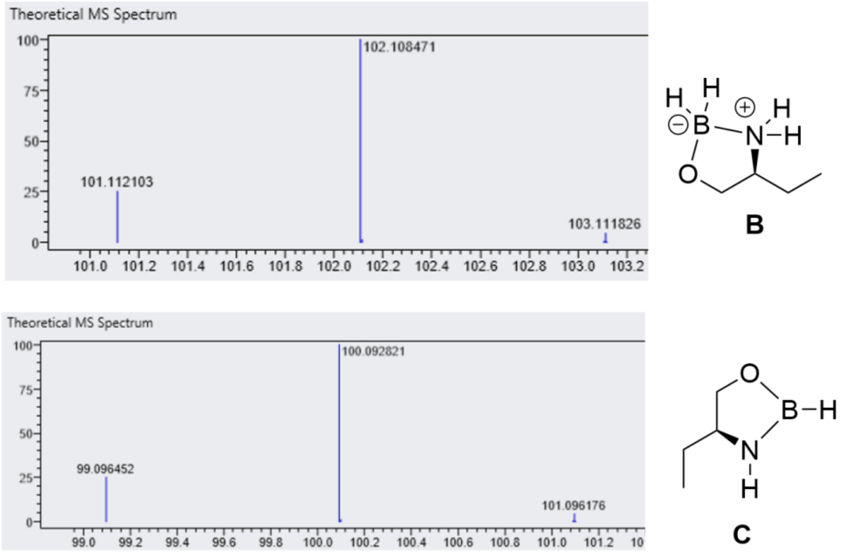

HRMS (ESI) spectrum of 1:1-mixture 2c + BH_3•SMe_2 in toluene at room temperature after 0.5 h reaction time

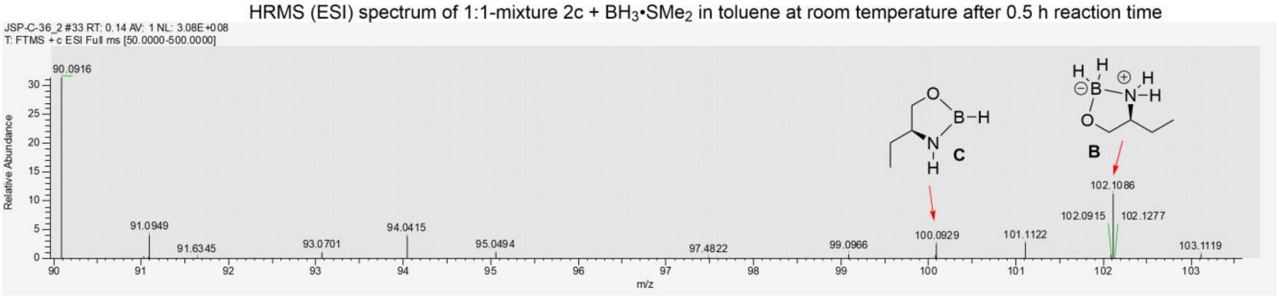

HRMS (ESI) spectrum of 1:1-mixture 2c + BH_3•SMe_2 in toluene at room temperature after 48 h reaction time

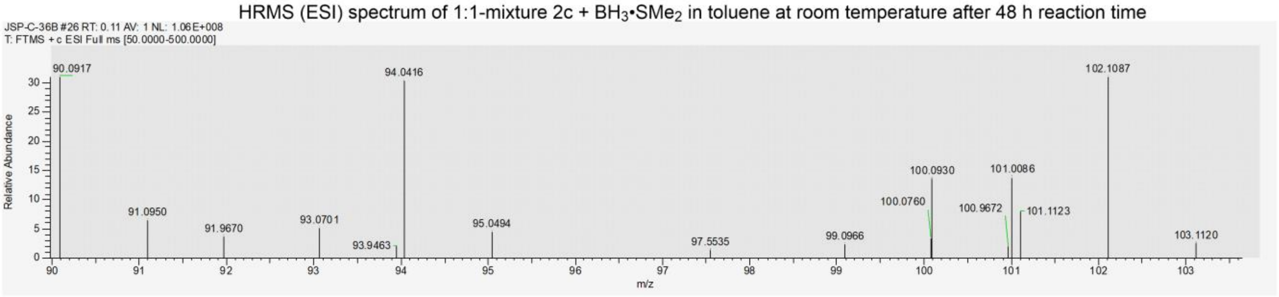

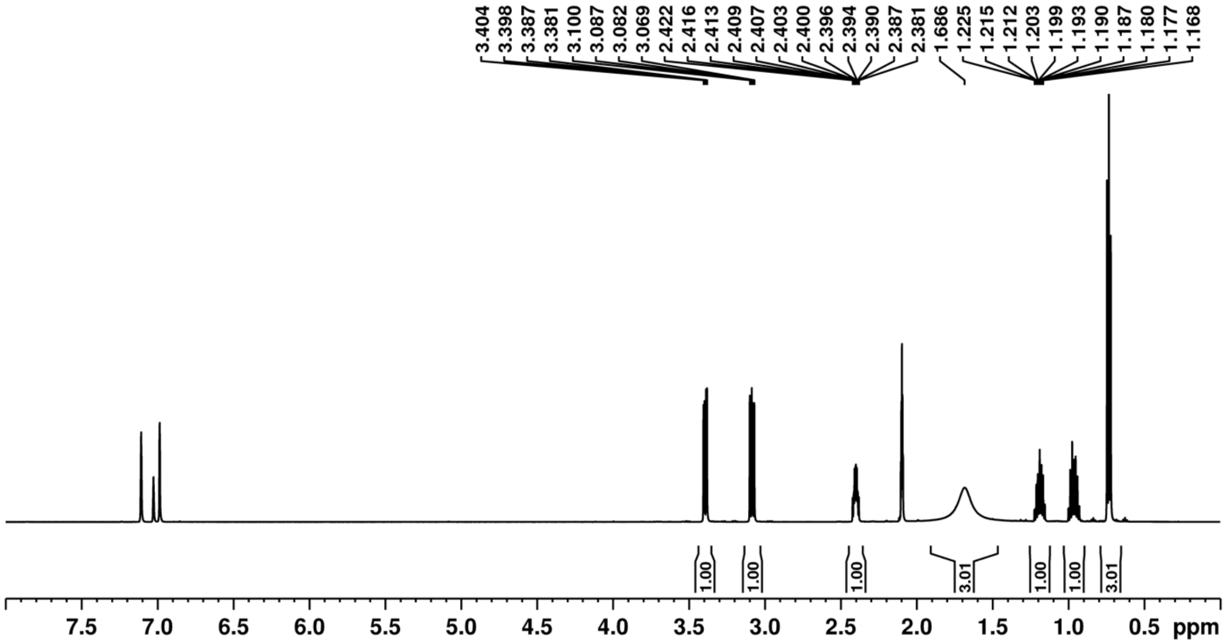

Figure S1. ^{1}H NMR spectrum of ligand **2c** in toluene-d_8.

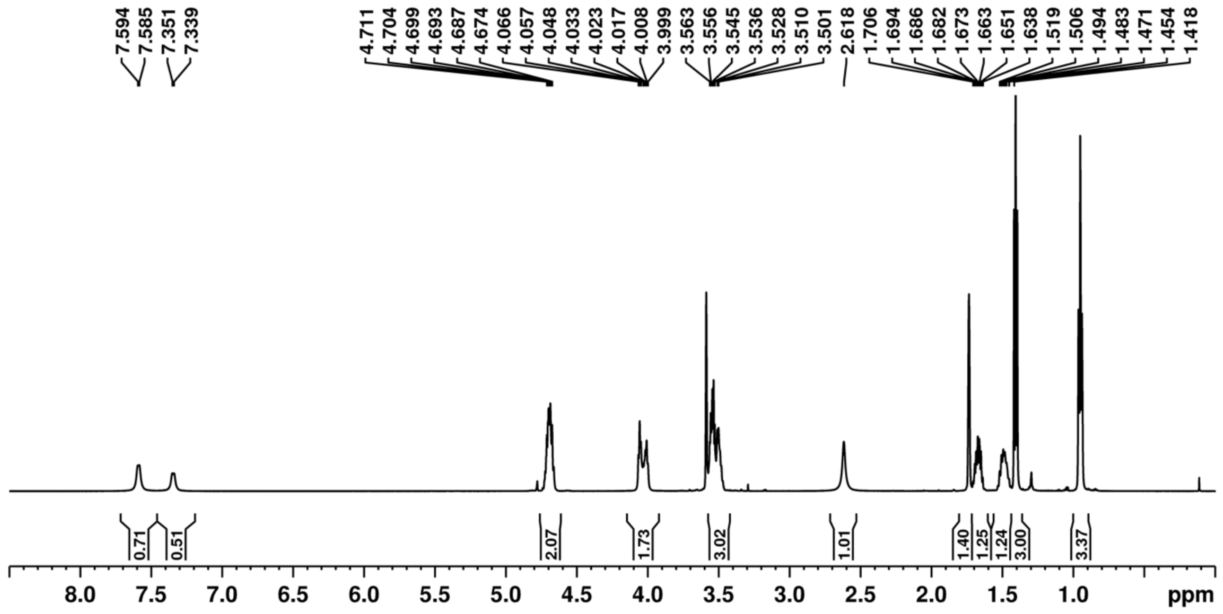

Figure S2. ^{1}H NMR spectrum of ligand **5** in THF-d_8.

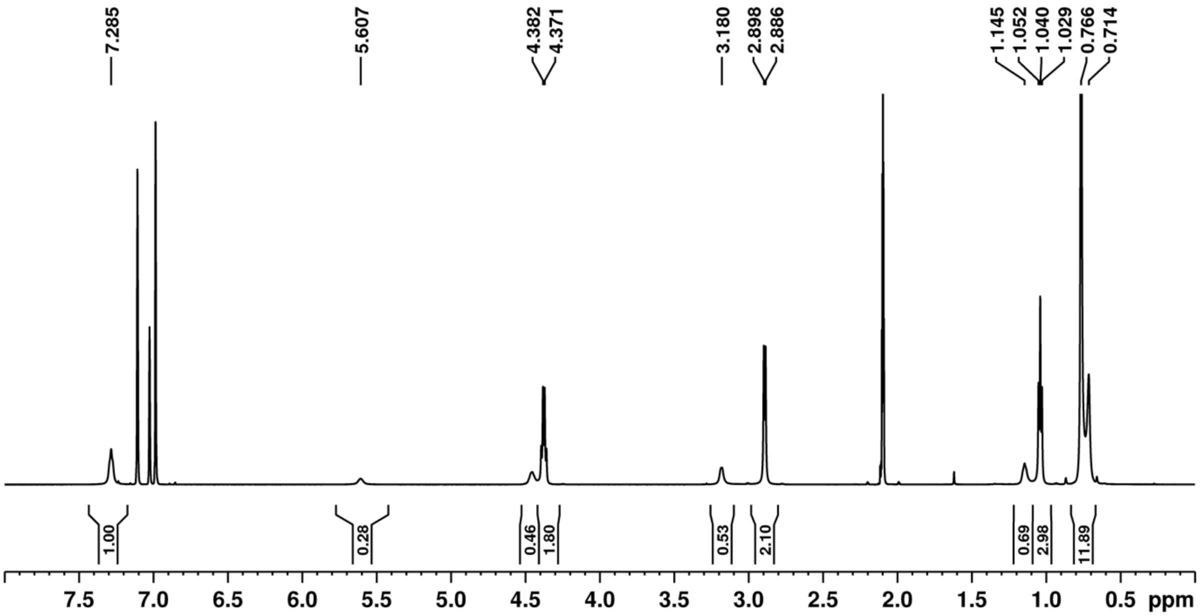

Figure S3. ^{1}H NMR spectrum of ligand **6** in toluene-d_8.

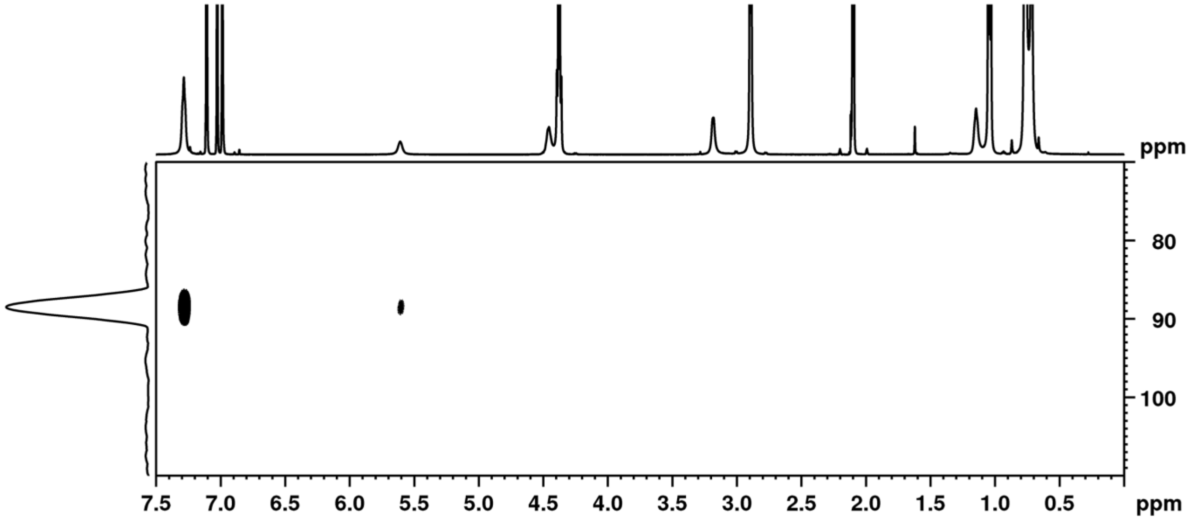

Figure S4. ^{1}H-^{15}N HMBC NMR spectrum of ligand **5** in THF-d_8.

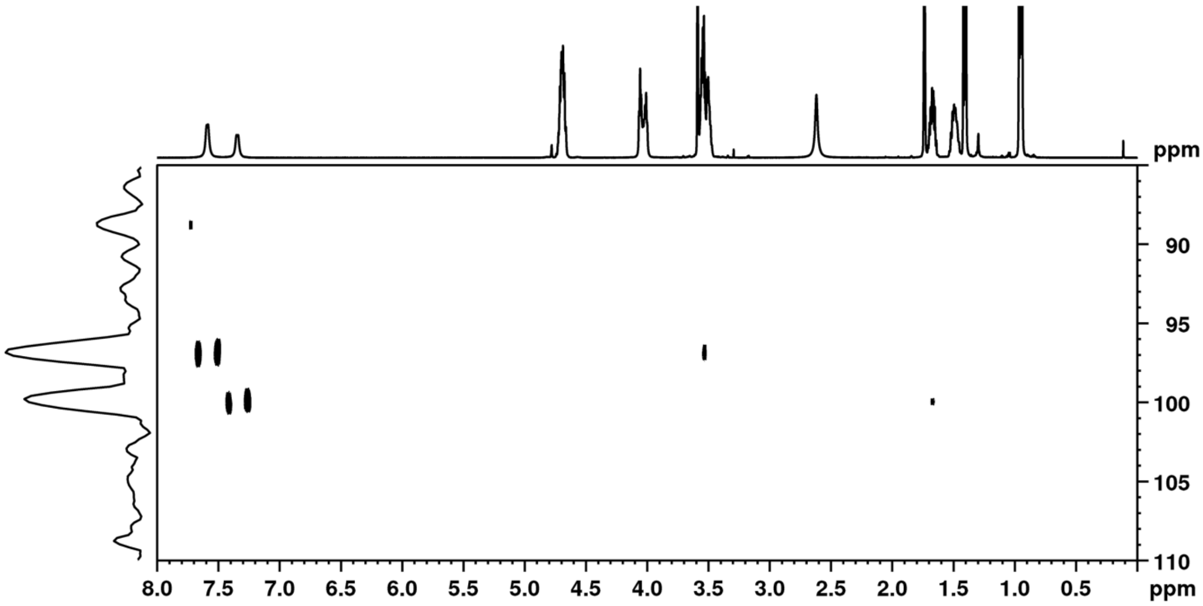

Figure S5. ^{1}H-^{15}N HSQC NMR spectrum of ligand **6** in THF-d_8.

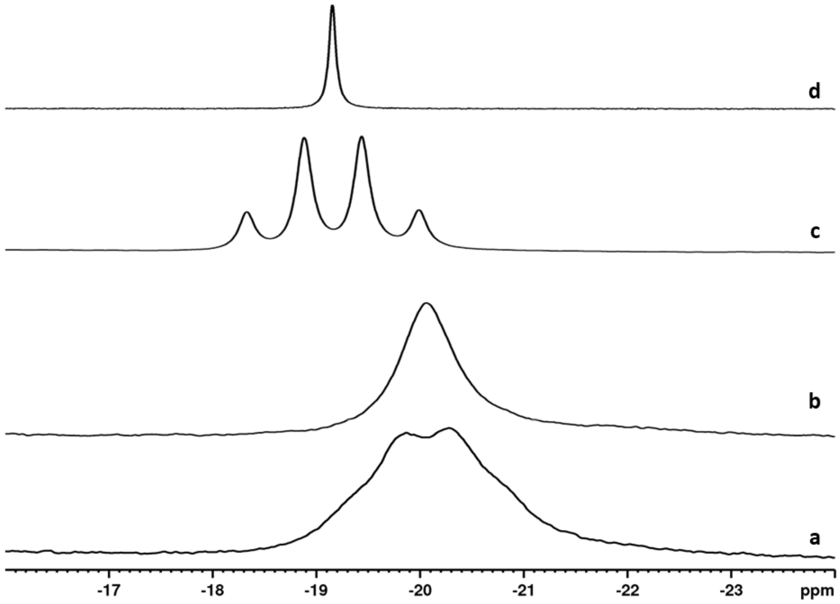

Figure S6. a) ^{11}B and b) $^{11}B\{1H\}$ spectra of 0.1 M mixture of ligand **2c** and BH_3•SMe_2 (1:1 molar ratio) in toluene-d_8 at room temperature; c) ^{11}B and d) $^{11}B\{1H\}$ spectra of pure BH_3•SMe_2 in toluene-d_8.

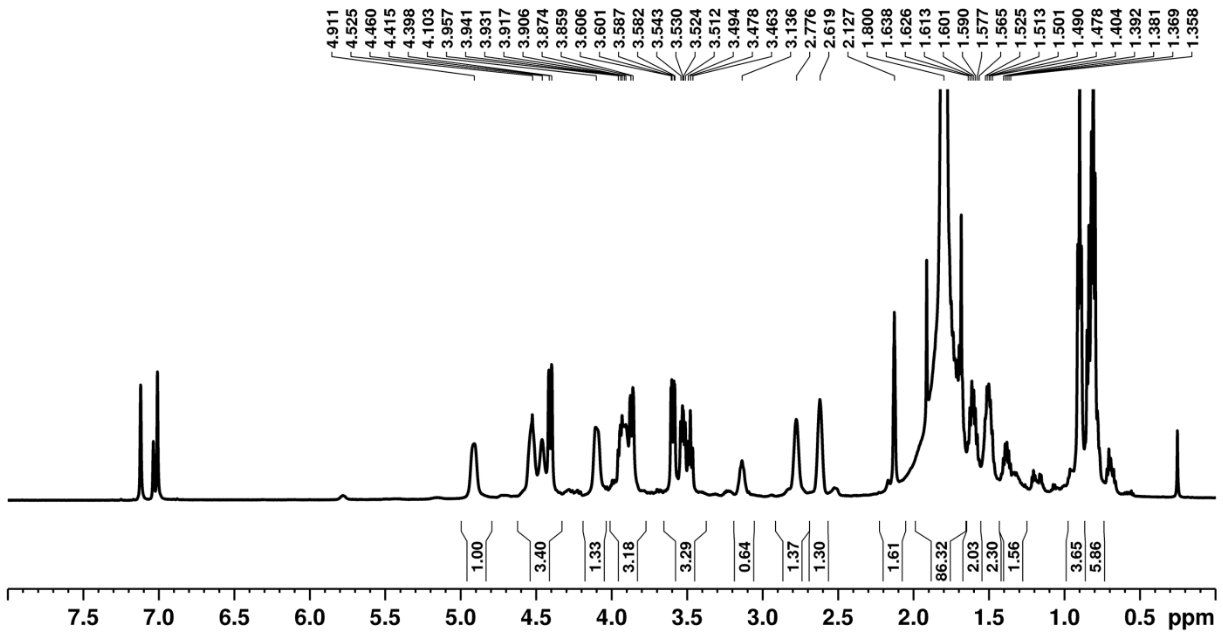

Figure S7a. ^{1}H NMR spectrum of 2 M mixture of ligand **2c** and BH_3•SMe_2 in toluene-d_8.

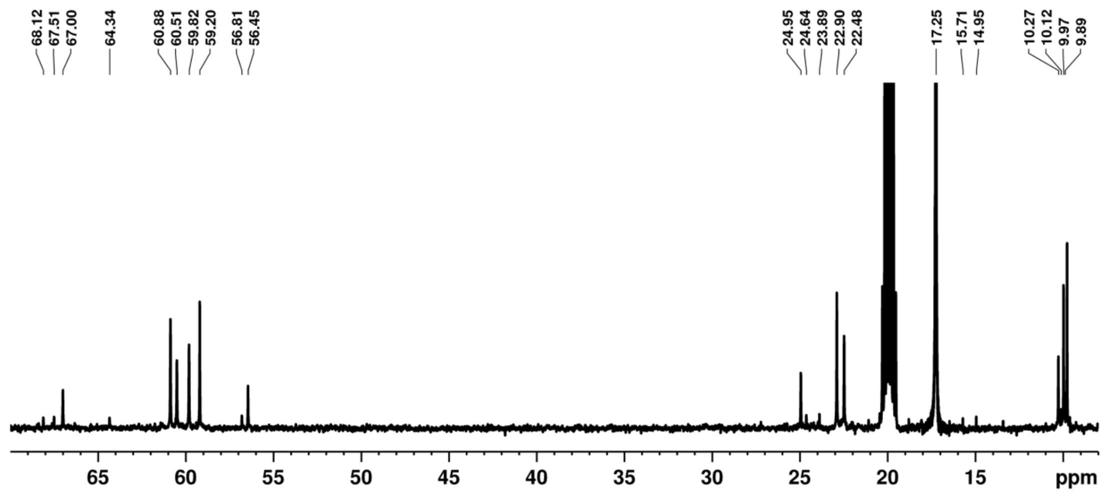

Figure S7b. $^{13}C\{^{1}H\}$ NMR spectrum of 2 M mixture of ligand **2c** and BH_3•SMe_2 in toluene-d_8.

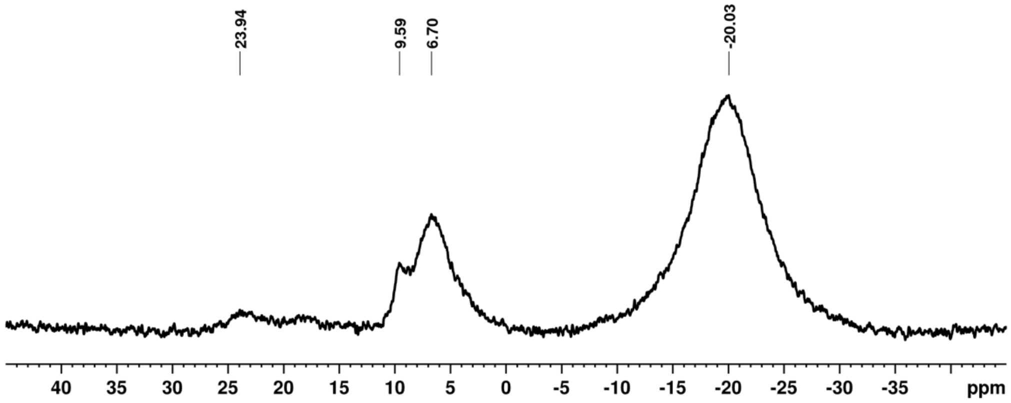

Figure S8. ^{10}B NMR spectrum of 2 M mixture of ligand **2c** and $BH_3•SMe_2$ in toluene-d_8.

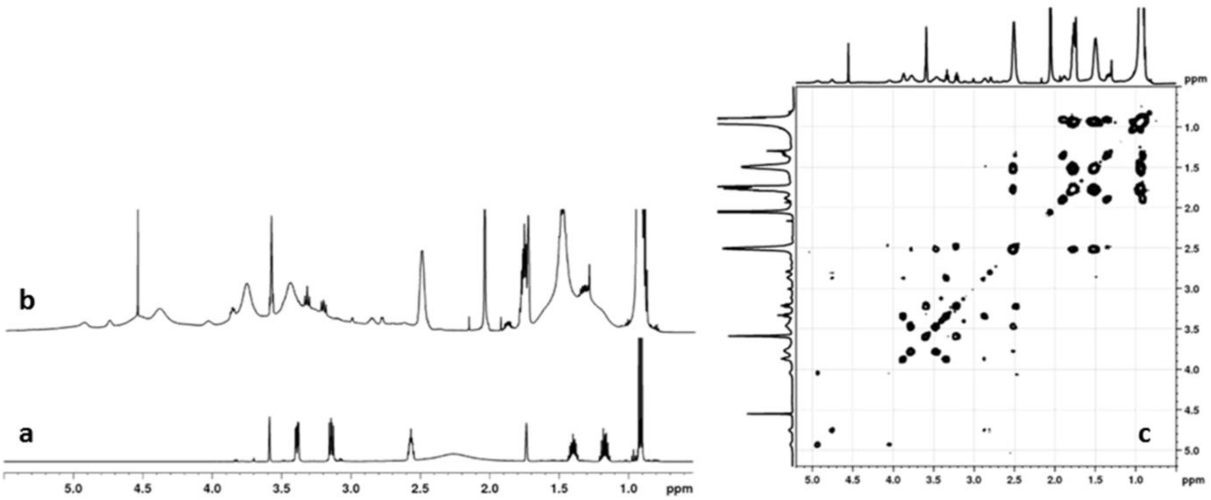

Figure S9. ^1H NMR spectra of: a) pure ligand **2c** in THF-d_8, b) mixture of **2c** and **$BH_3•SMe_2$** in THF-d_8, c) COSY spectrum of mixture of **2c** and **$BH_3•SMe_2$** in THF-d_8.

Images of ^1H and ^{13}C NMR spectra of synthesized compounds:

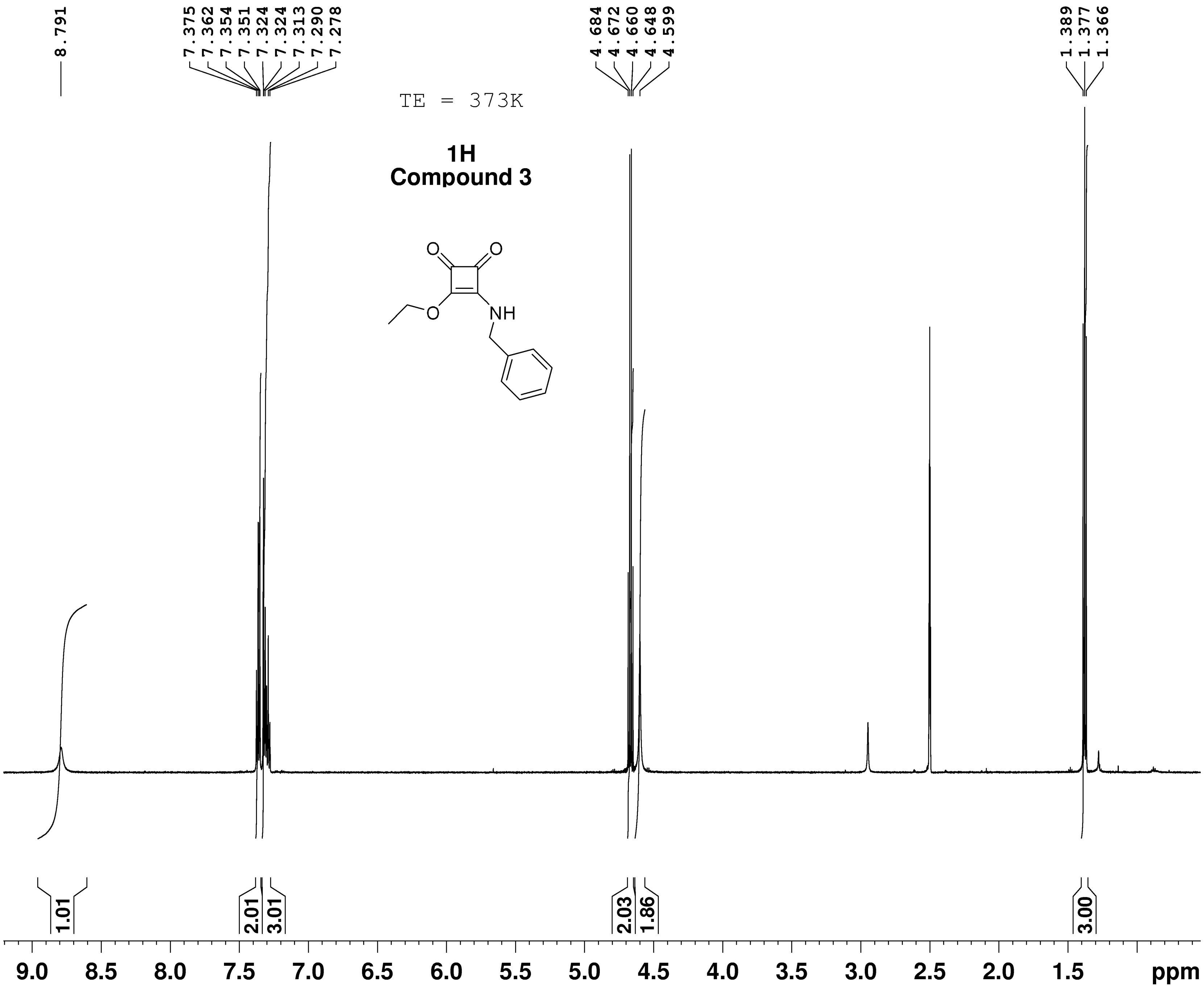

NAME	YNC-031-03A	
EXPNO	30	
PROCNO	1	
Date_	20190419	
Time	16.04	h
INSTRUM	spect	
PROBHD	Z847801_0047 (	
PULPROG	zg30	
TD	32768	
SOLVENT	DMSO	
NS	1	
DS	0	
SWH	9615.385	Hz
FIDRES	0.586877	Hz
AQ	1.7039860	sec
RG	144	
DW	52.000	usec
DE	13.95	usec
TE	373.1	K
D1	1.00000000	sec
TD0	1	
SFO1	600.0145608	MHz
NUC1	1H	
P1	10.85	usec
SI	65536	
SF	600.0100043	MHz
WDW	no	
SSB	0	
LB	0.00	Hz
GB	0	
PC	1.00	

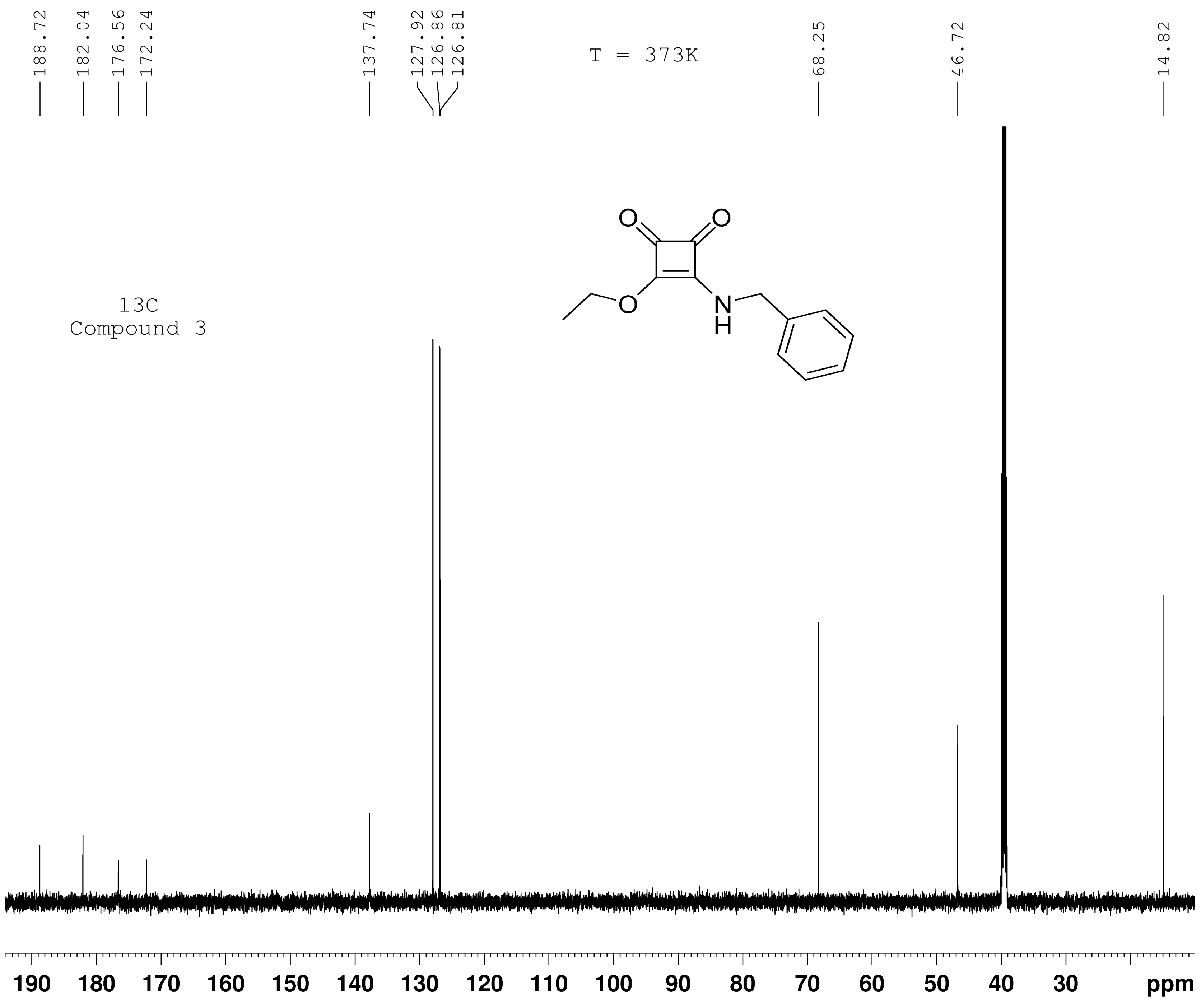

NAME	YNC-031-03A
EXPNO	32
PROCNO	1
Date_	20190419
Time	16.24 h
INSTRUM	spect
PROBHD	Z847801_0047 (
PULPROG	zgdc30
TD	32768
SOLVENT	DMSO
NS	512
DS	0
SWH	36057.691 Hz
FIDRES	2.200787 Hz
AQ	0.4544329 sec
RG	2050
DW	13.867 usec
DE	6.50 usec
TE	373.2 K
D1	1.50000000 sec
D11	0.03000000 sec
TD0	1
SFO1	150.8892338 MHz
NUC1	13C
P1	9.80 usec
SI	65536
SF	150.8727892 MHz
WDW	EM
SSB	0
LB	1.00 Hz
GB	0
PC	1.40

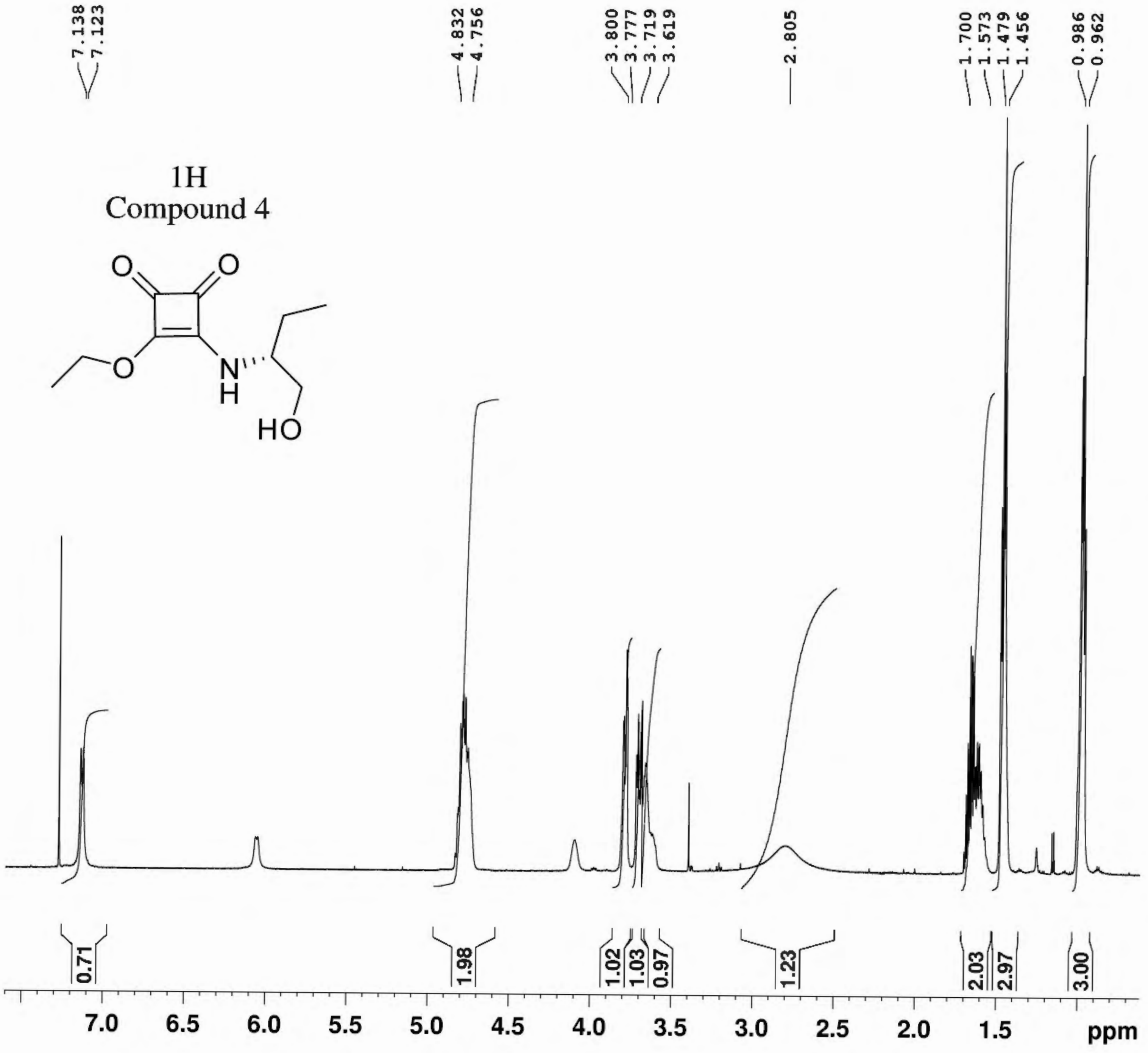

NAME	NB_063_03
EXPNO	11
PROCNO	1
Date_	20140402
Time	18.59
INSTRUM	spect
PROBHD	5 mm PABBO BB-
PULPROG	zg30
TD	32768
SOLVENT	CDCl3
NS	16
DS	0
SWH	9615.385 Hz
FIDRES	0.293438 Hz
AQ	1.7039860 sec
RG	144
DW	52.000 usec
DE	13.95 usec
TE	293.0 K
D1	1.00000000 sec
TD0	1

======== CHANNEL f1 ========

SFO1	600.1345610 MHz
NUC1	1H
P1	10.85 usec
SI	65536
SF	600.1300119 MHz
WDW	EM
SSB	0
LB	0.10 Hz
GB	0
PC	1.00

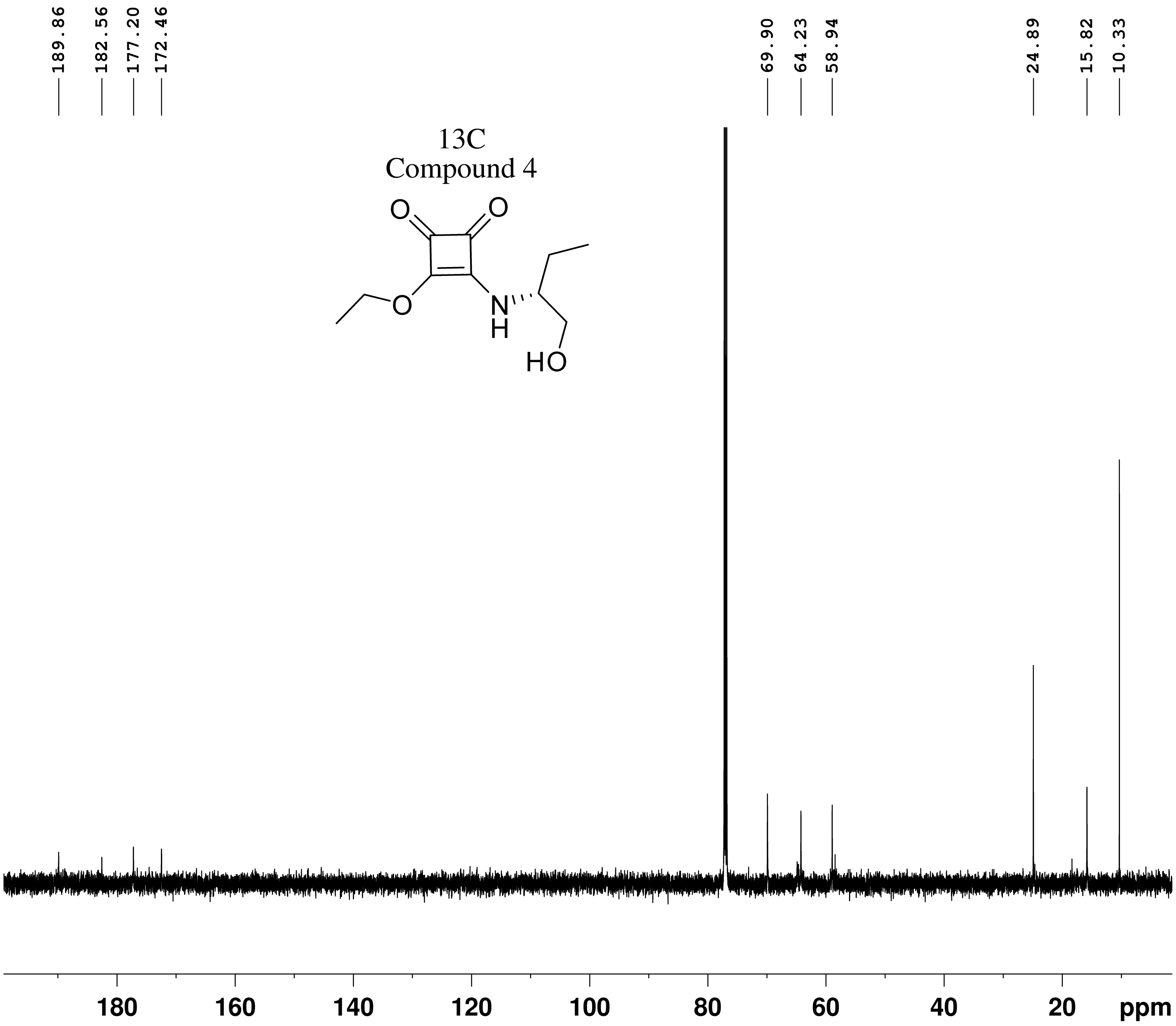

NAME	YNC04004B
EXPNO	12
PROCNO	1
Date_	20150513
Time	23.21
INSTRUM	spect
PROBHD	5 mm PABBO BB-
PULPROG	zgdc30
TD	32768
SOLVENT	CDCl3
NS	512
DS	0
SWH	36057.691 Hz
FIDRES	1.100393 Hz
AQ	0.4544329 sec
RG	2050
DW	13.867 usec
DE	6.50 usec
TE	298.0 K
D1	1.50000000 sec
D11	0.03000000 sec
TD0	1

======== CHANNEL f1 ========

SFO1	150.9143788 MHz
NUC1	13C
P1	9.80 usec
SI	65536
SF	150.8977842 MHz
WDW	EM
SSB	0
LB	1.00 Hz
GB	0
PC	1.40

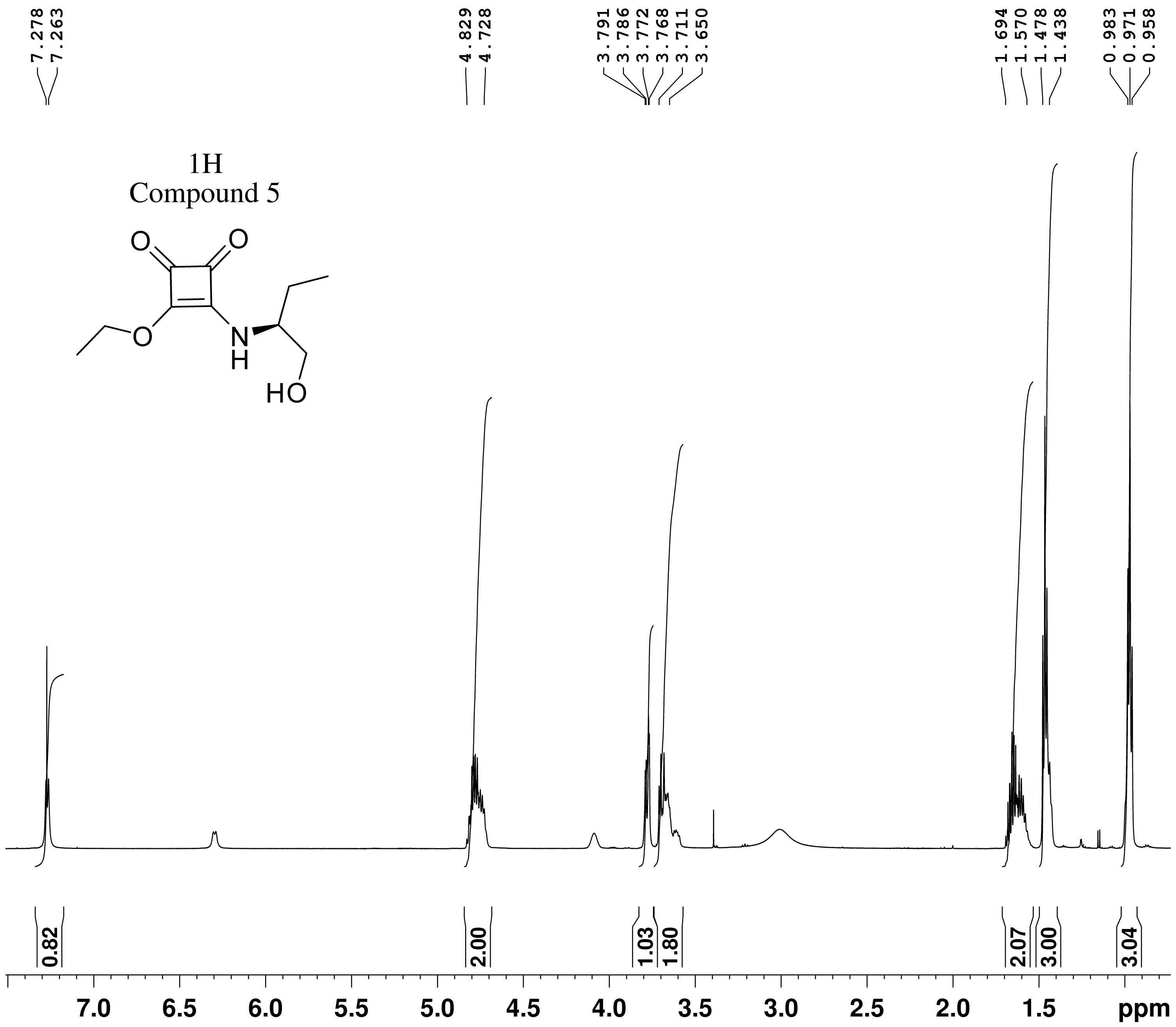

NAME	NB_077_02
EXPNO	11
PROCNO	1
Date_	20140605
Time	18.05
INSTRUM	spect
PROBHD	5 mm PABBO BB-
PULPROG	zg30
TD	32768
SOLVENT	CDCl3
NS	16
DS	0
SWH	9615.385 Hz
FIDRES	0.293438 Hz
AQ	1.7039860 sec
RG	144
DW	52.000 usec
DE	13.95 usec
TE	293.0 K
D1	1.00000000 sec
TD0	1

======== CHANNEL f1 ========

SFO1	600.1345610 MHz
NUC1	1H
P1	10.85 usec
SI	65536
SF	600.1300069 MHz
WDW	EM
SSB	0
LB	0.10 Hz
GB	0
PC	1.00

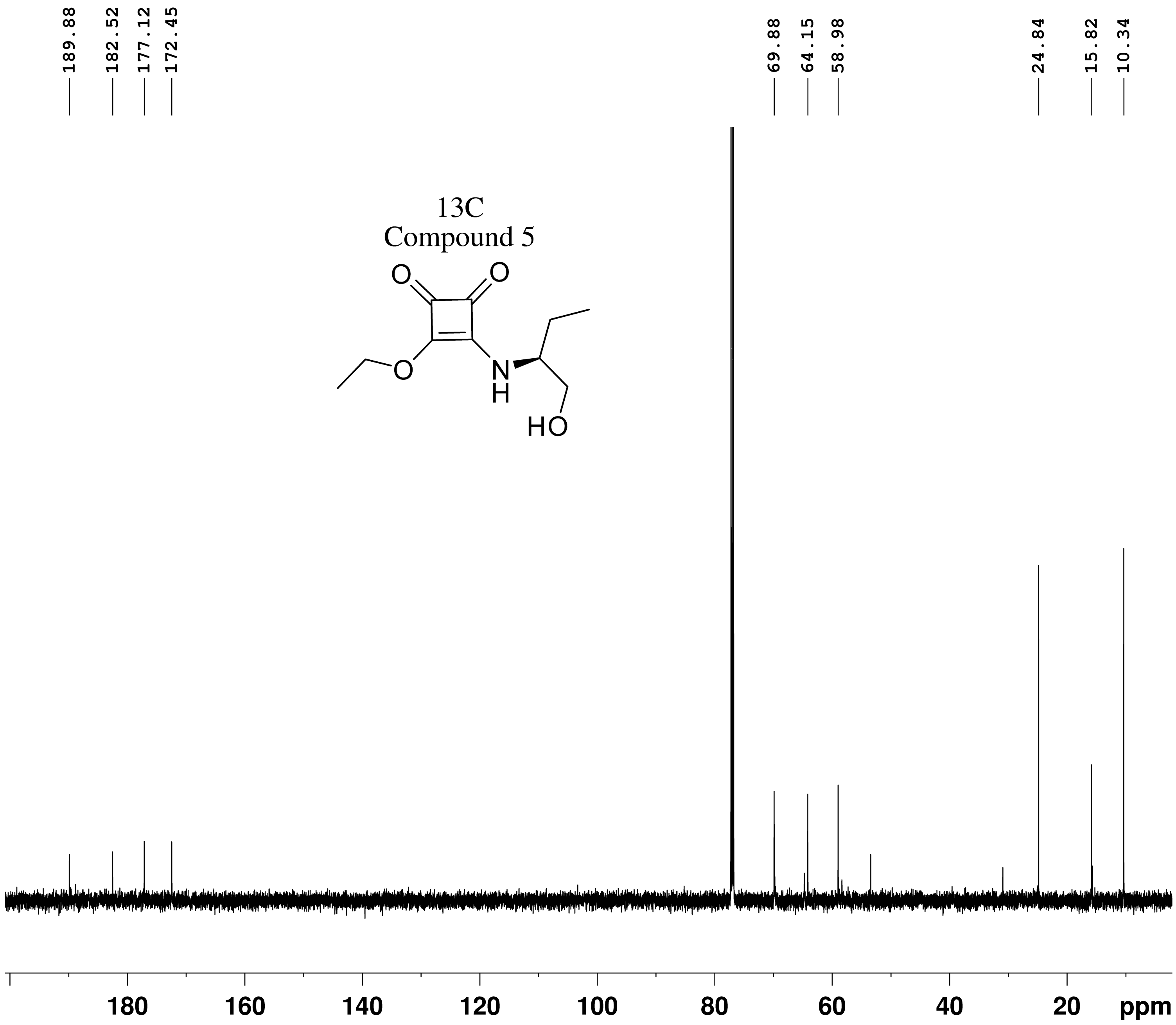

NAME	DL-175A
EXPNO	12
PROCNO	1
Date_	20140207
Time	18.17
INSTRUM	spect
PROBHD	5 mm PABBO BB-
PULPROG	zgdc30
TD	32768
SOLVENT	CDCl3
NS	128
DS	0
SWH	36057.691 Hz
FIDRES	1.100393 Hz
AQ	0.4544329 sec
RG	2050
DW	13.867 usec
DE	7.48 usec
TE	293.1 K
D1	1.50000000 sec
D11	0.03000000 sec
TD0	1

======== CHANNEL f1 ========

SFO1	150.9188042 MHz
NUC1	13C
P1	10.75 usec
SI	65536
SF	150.9028181 MHz
WDW	EM
SSB	0
LB	1.00 Hz
GB	0
PC	1.00

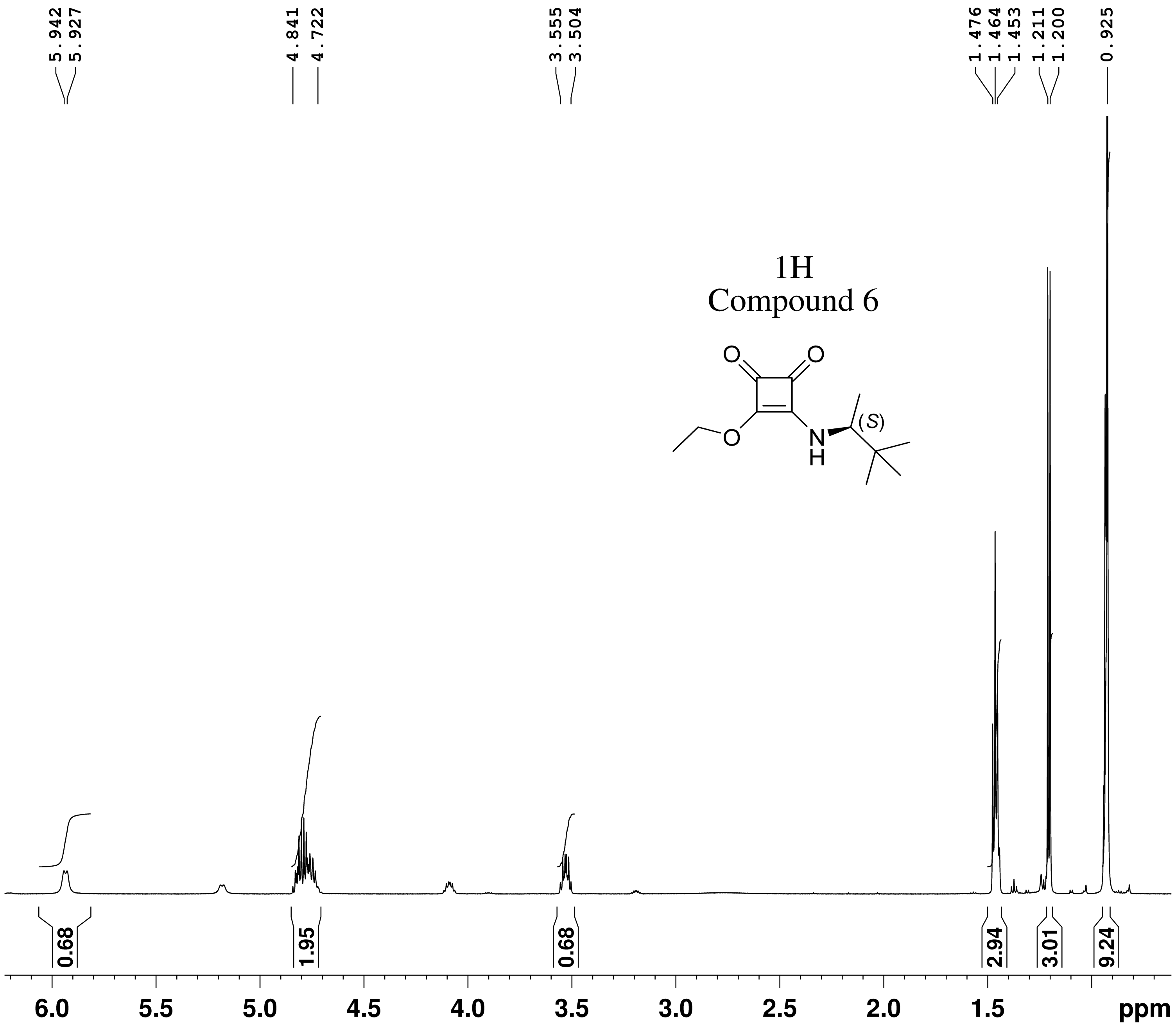

NAME	NB03902AB
EXPNO	21
PROCNO	1
Date_	20160830
Time	16.44
INSTRUM	spect
PROBHD	5 mm PABBO BB-
PULPROG	zg30
TD	32768
SOLVENT	CDCl3
NS	16
DS	0
SWH	9615.385 Hz
FIDRES	0.293438 Hz
AQ	1.7039860 sec
RG	203
DW	52.000 usec
DE	13.95 usec
TE	293.0 K
D1	1.00000000 sec
TD0	1

======== CHANNEL f1 ========

SFO1	600.1145608 MHz
NUC1	1H
P1	10.85 usec
SI	65536
SF	600.1100148 MHz
WDW	EM
SSB	0
LB	0.00 Hz
GB	0
PC	1.00

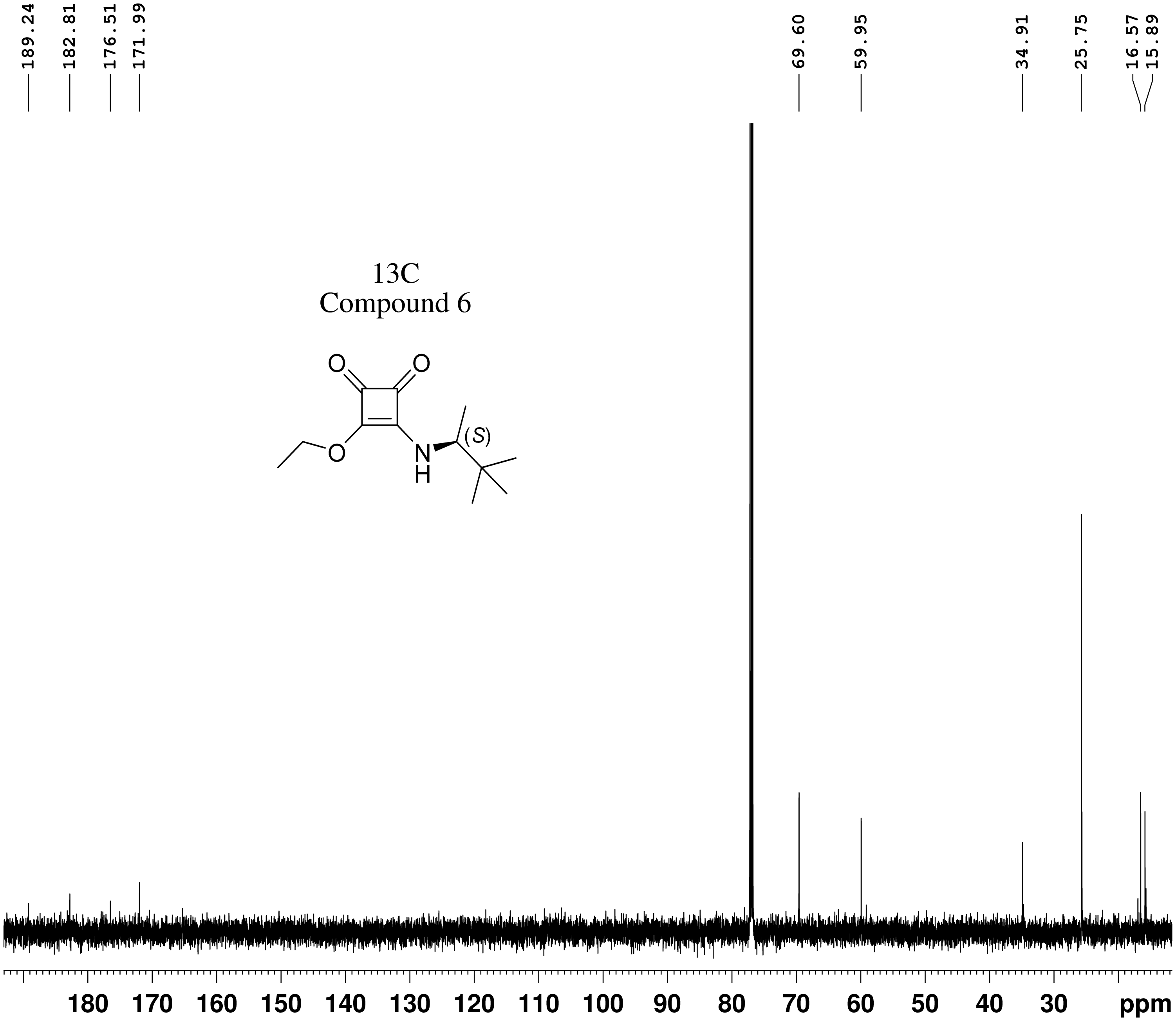

13C
Compound 6

NAME	NB03902AB
EXPNO	22
PROCNO	1
Date_	20160830
Time	16.48
INSTRUM	spect
PROBHD	5 mm PABBO BB-
PULPROG	zgdc30
TD	32768
SOLVENT	CDCl3
NS	128
DS	0
SWH	36057.691 Hz
FIDRES	1.100393 Hz
AQ	0.4544329 sec
RG	2050
DW	13.867 usec
DE	6.50 usec
TE	293.0 K
D1	1.50000000 sec
D11	0.03000000 sec
TD0	1

======== CHANNEL f1 ========

SFO1	150.9143788 MHz
NUC1	13C
P1	9.80 usec
SI	65536
SF	150.8977867 MHz
WDW	EM
SSB	0
LB	1.00 Hz
GB	0
PC	1.40

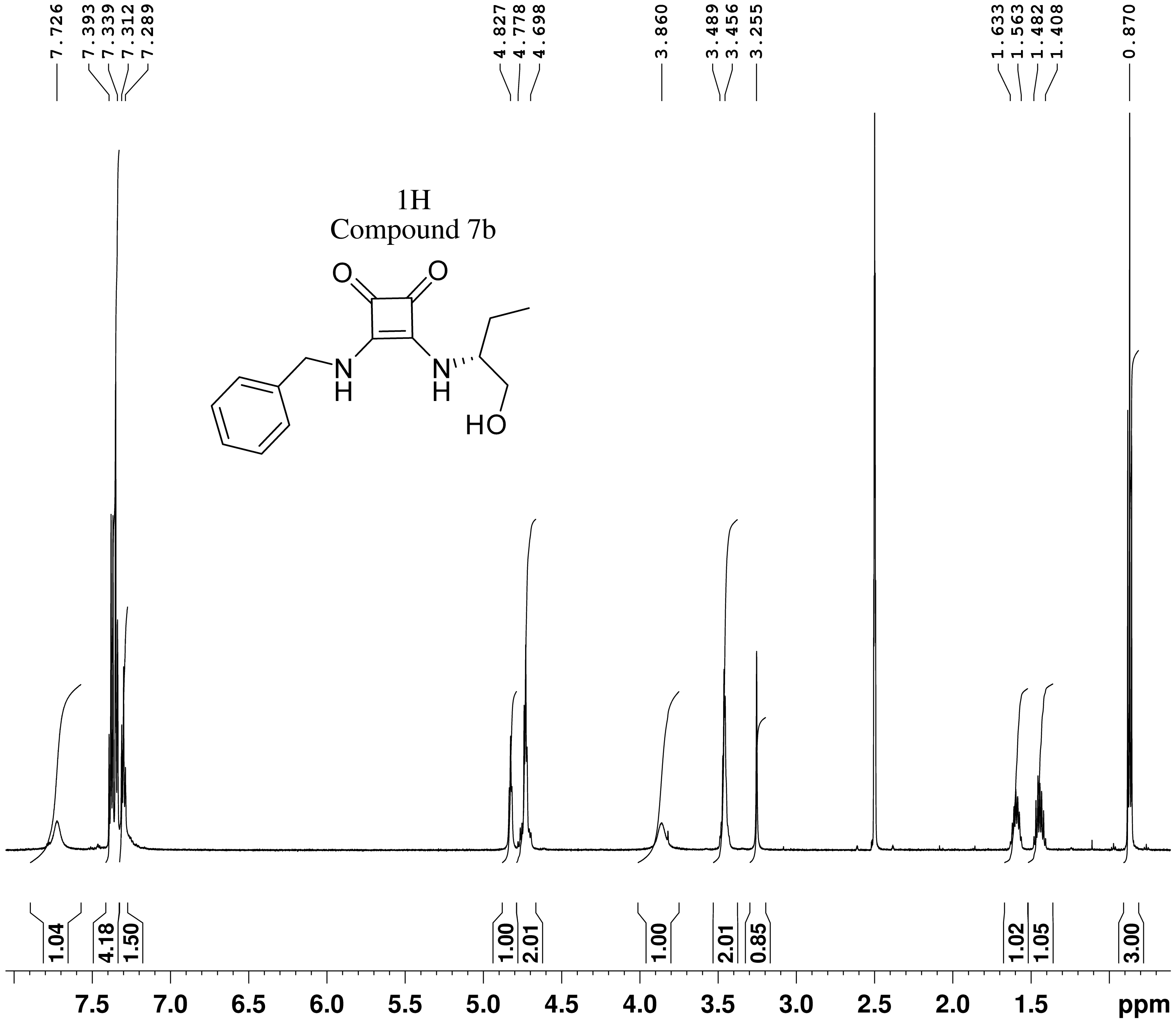

NAME	NB67B	
EXPNO	1	
PROCNO	1	
Date_	20140627	
Time	13.57	
INSTRUM	spect	
PROBHD	5 mm PABBO BB-	
PULPROG	zg30	
TD	32768	
SOLVENT	DMSO	
NS	1	
DS	0	
SWH	9615.385	Hz
FIDRES	0.293438	Hz
AQ	1.7039860	sec
RG	144	
DW	52.000	usec
DE	13.95	usec
TE	313.0	K
D1	1.00000000	sec
TD0	1	

======== CHANNEL f1 ========

SFO1	600.1345610	MHz
NUC1	1H	
P1	10.85	usec
SI	65536	
SF	600.1300049	MHz
WDW	no	
SSB	0	
LB	0.00	Hz
GB	0	
PC	1.00	

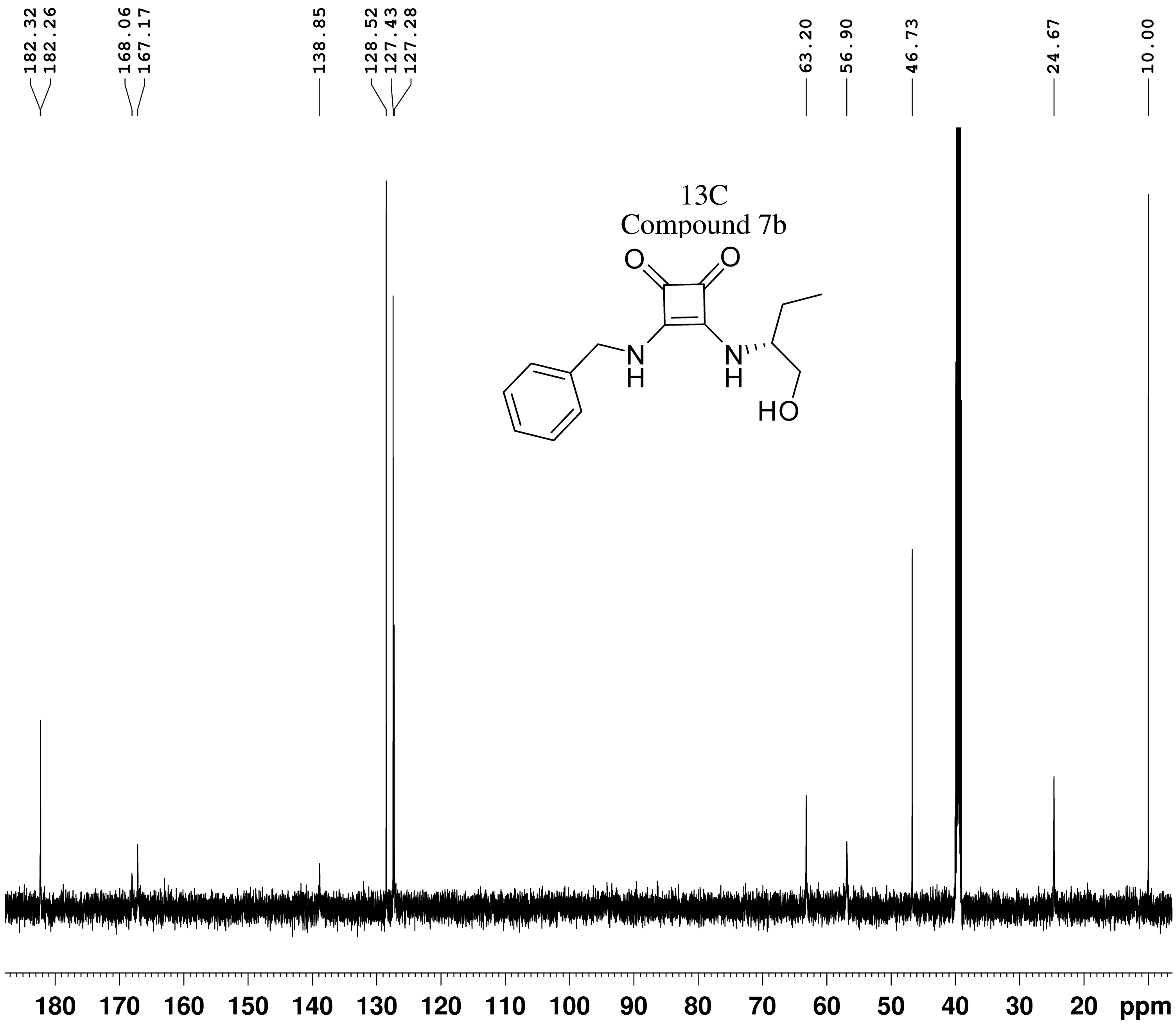

NAME	NB67B
EXPNO	3
PROCNO	2
Date_	20140627
Time	14.33
INSTRUM	spect
PROBHD	5 mm PABBO BB-
PULPROG	zgdc30
TD	32768
SOLVENT	DMSO
NS	512
DS	0
SWH	36057.691 Hz
FIDRES	1.100393 Hz
AQ	0.4544329 sec
RG	2050
DW	13.867 usec
DE	7.48 usec
TE	313.0 K
D1	1.50000000 sec
D11	0.03000000 sec
TD0	1

======== CHANNEL f1 ========

SFO1	150.9188042 MHz
NUC1	13C
P1	10.75 usec
SI	65536
SF	150.9028994 MHz
WDW	EM
SSB	0
LB	1.00 Hz
GB	0
PC	1.00

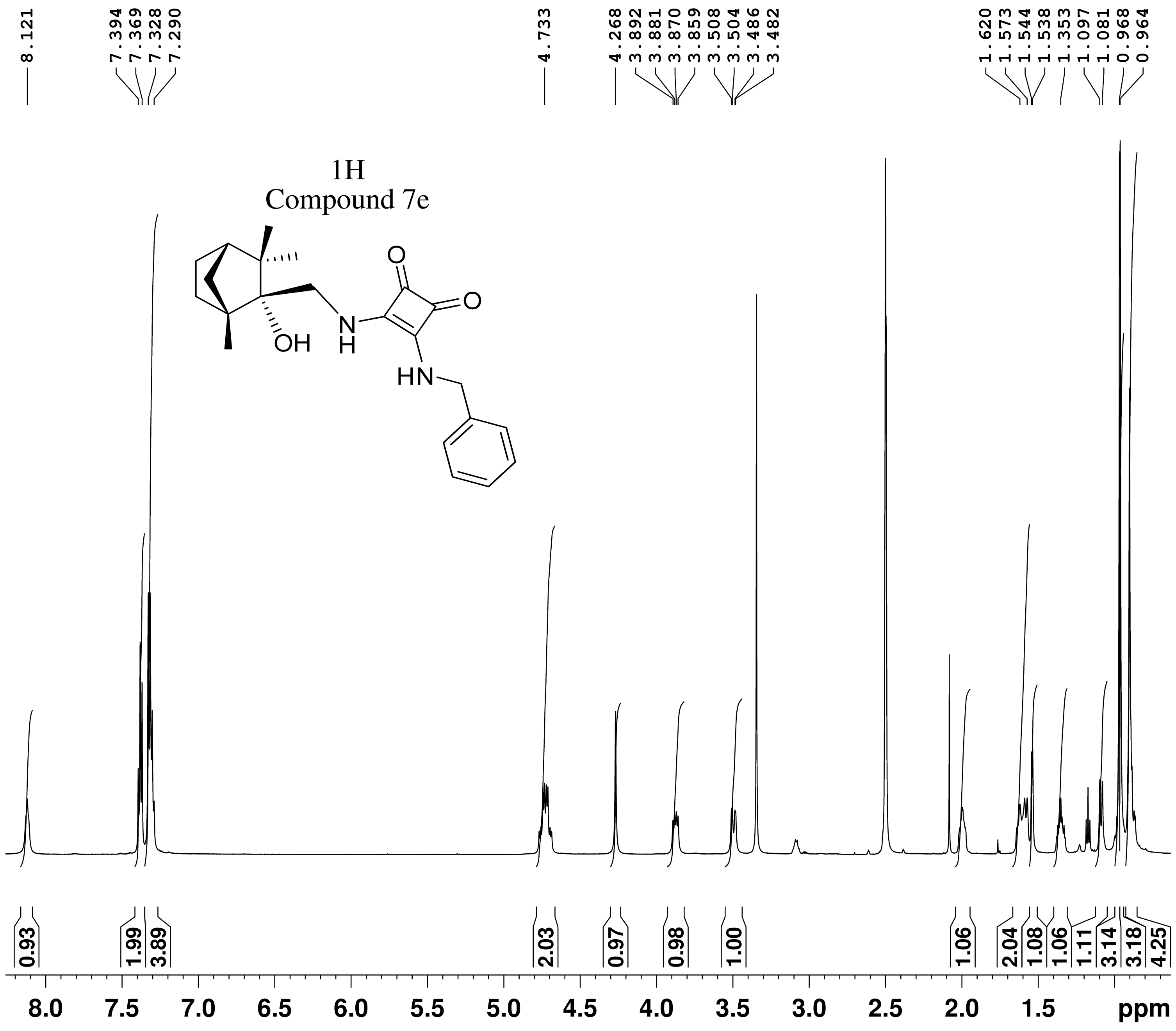

NAME	YNC00703A
EXPNO	11
PROCNO	1
Date_	20141121
Time	22.05
INSTRUM	spect
PROBHD	5 mm PABBO BB-
PULPROG	zg30
TD	32768
SOLVENT	DMSO
NS	32
DS	0
SWH	9615.385 Hz
FIDRES	0.293438 Hz
AQ	1.7039860 sec
RG	144
DW	52.000 usec
DE	13.95 usec
TE	293.0 K
D1	1.00000000 sec
TD0	1

======== CHANNEL f1 ========

SFO1	600.1345610 MHz
NUC1	1H
P1	10.85 usec
SI	65536
SF	600.1300046 MHz
WDW	EM
SSB	0
LB	0.10 Hz
GB	0
PC	1.00

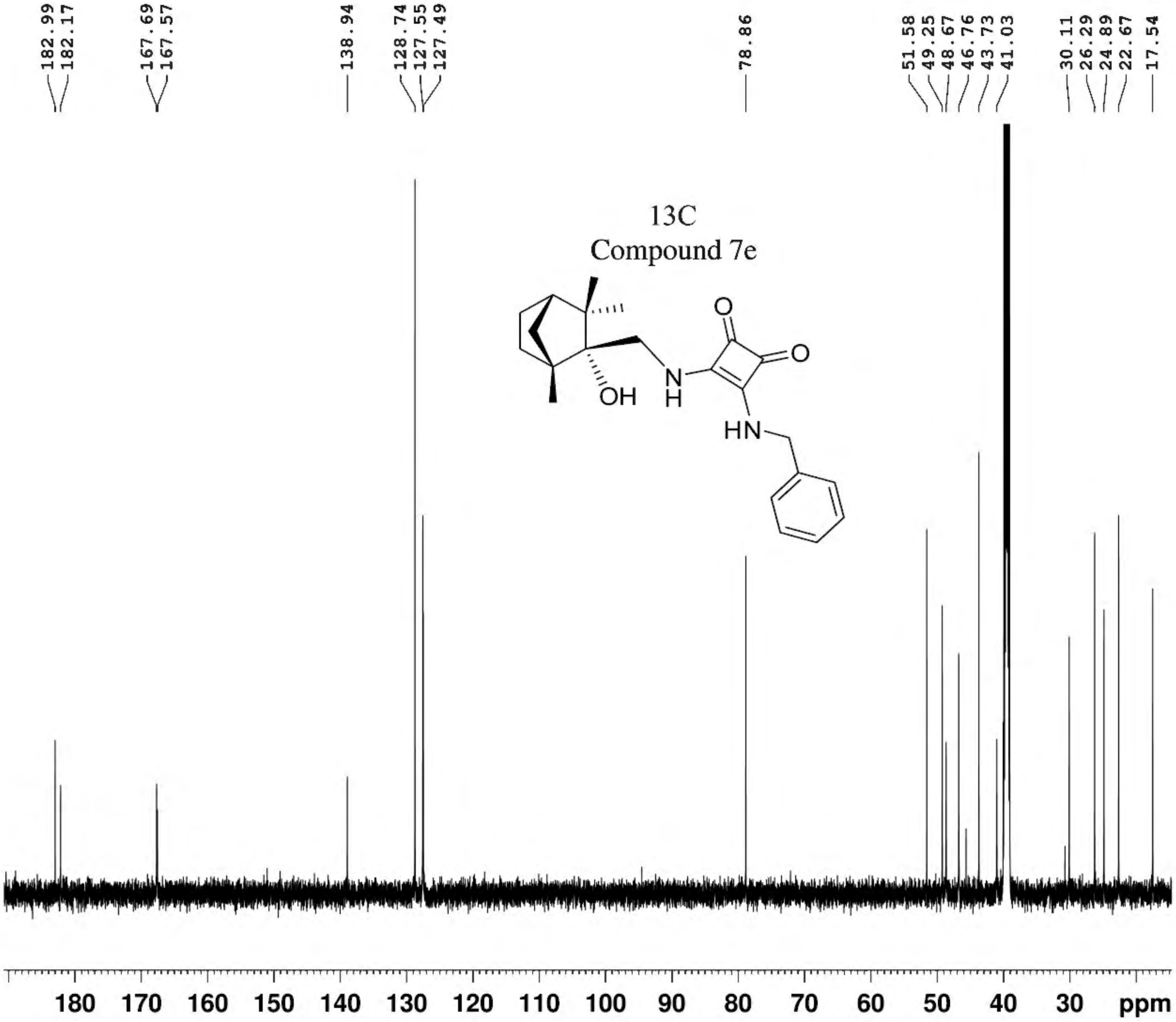

NAME	YNC00703A	
EXPNO	12	
PROCNO	1	
Date_	20141121	
Time	22.39	
INSTRUM	spect	
PROBHD	5 mm PABBO BB-	
PULPROG	zgdc30	
TD	32768	
SOLVENT	DMSO	
NS	1024	
DS	0	
SWH	36057.691	Hz
FIDRES	1.100393	Hz
AQ	0.4544329	sec
RG	2050	
DW	13.867	usec
DE	7.68	usec
TE	293.0	K
D1	1.50000000	sec
D11	0.03000000	sec
TD0	1	
========	CHANNEL f1	========
SFO1	150.9188042	MHz
NUC1	13C	
P1	9.80	usec
SI	65536	
SF	150.9028750	MHz
WDW	EM	
SSB	0	
LB	1.00	Hz
GB	0	
PC	1.00	

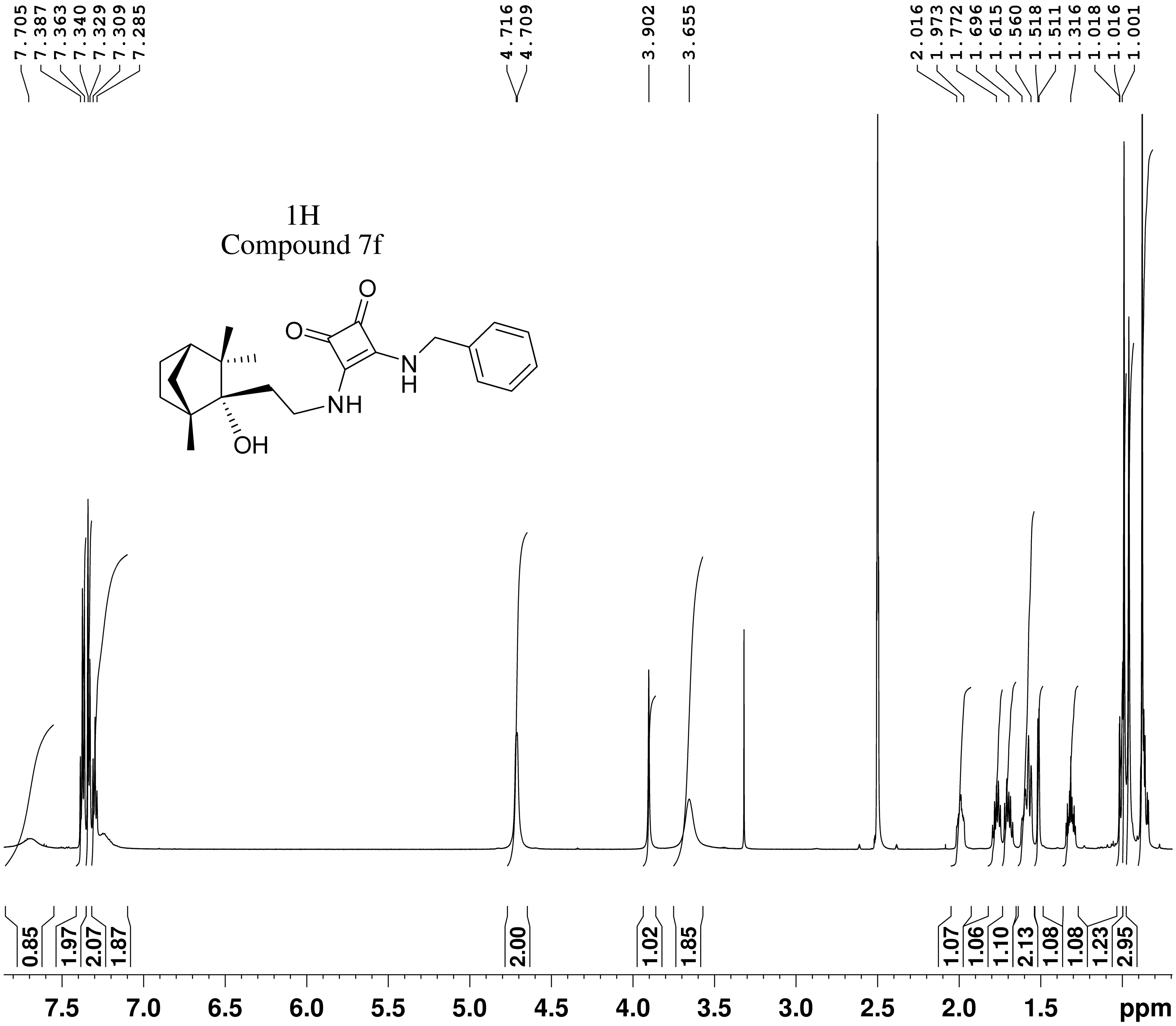

NAME	NB_074_02A	
EXPNO	11	
PROCNO	1	
Date_	20150511	
Time	20.33	
INSTRUM	spect	
PROBHD	5 mm PABBO BB-	
PULPROG	zg30	
TD	32768	
SOLVENT	DMSO	
NS	32	
DS	0	
SWH	9615.385	Hz
FIDRES	0.293438	Hz
AQ	1.7039860	sec
RG	144	
DW	52.000	usec
DE	13.95	usec
TE	298.0	K
D1	1.00000000	sec
TD0	1	

======== CHANNEL f1 ========

SFO1	600.1145608	MHz
NUC1	1H	
P1	10.85	usec
SI	65536	
SF	600.1100055	MHz
WDW	EM	
SSB	0	
LB	0.00	Hz
GB	0	
PC	1.00	

13C
Compound 7f

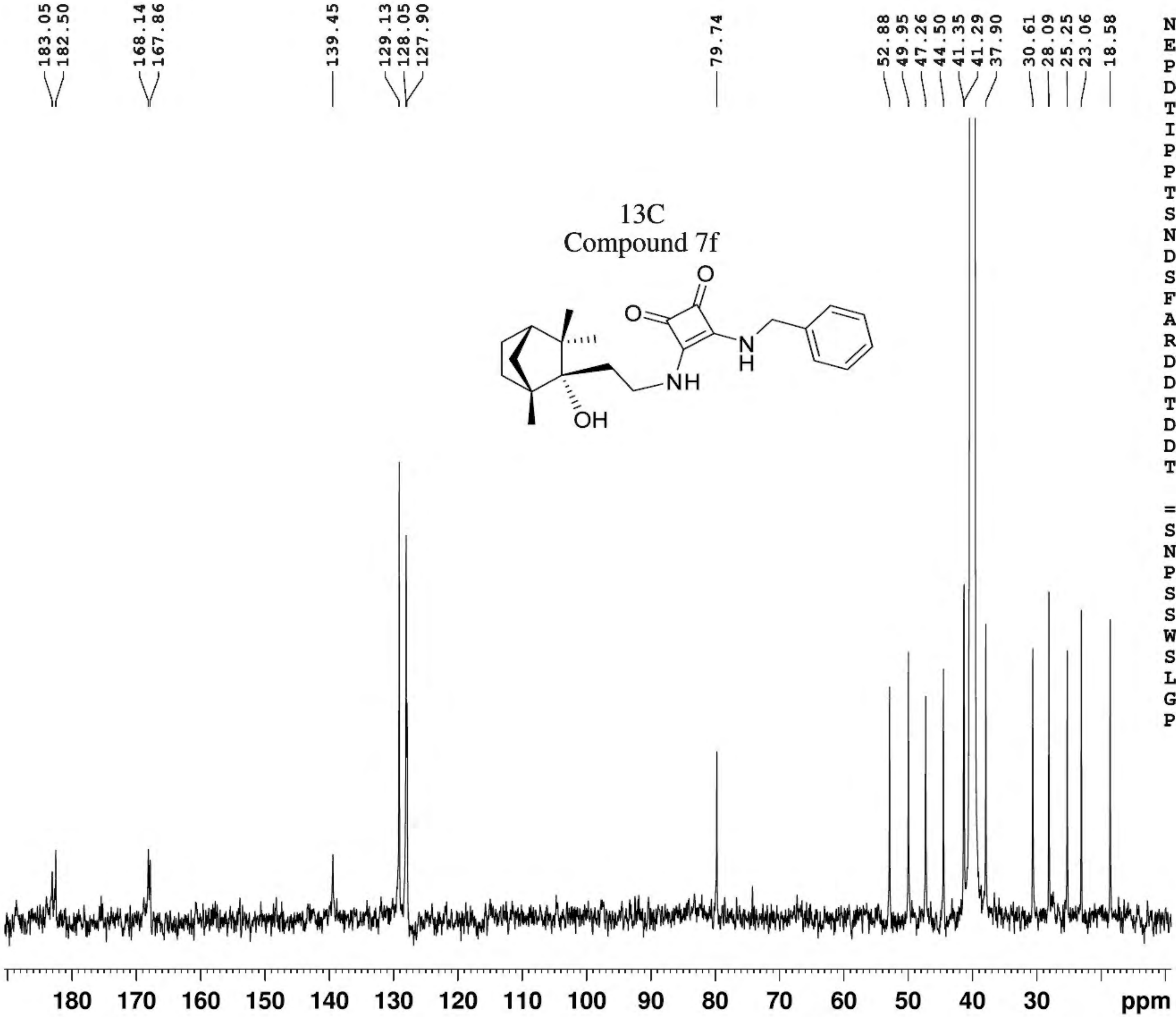

NAME	NB_074_02BB
EXPNO	12
PROCNO	1
Date_	20150513
Time	22.46
INSTRUM	spect
PROBHD	5 mm PABBO BB-
PULPROG	zgdc30
TD	32768
SOLVENT	DMSO
NS	512
DS	0
SWH	36057.691 Hz
FIDRES	1.100393 Hz
AQ	0.4544329 sec
RG	2050
DW	13.867 use
DE	6.50 use
TE	298.0 K
D1	1.50000000 sec
D11	0.03000000 sec
TD0	1

======== CHANNEL f1 ========

SFO1	150.9143788 MHz
NUC1	13C
P1	9.80 use
SI	65536
SF	150.8977804 MHz
WDW	EM
SSB	0
LB	10.00 Hz
GB	0
PC	1.40

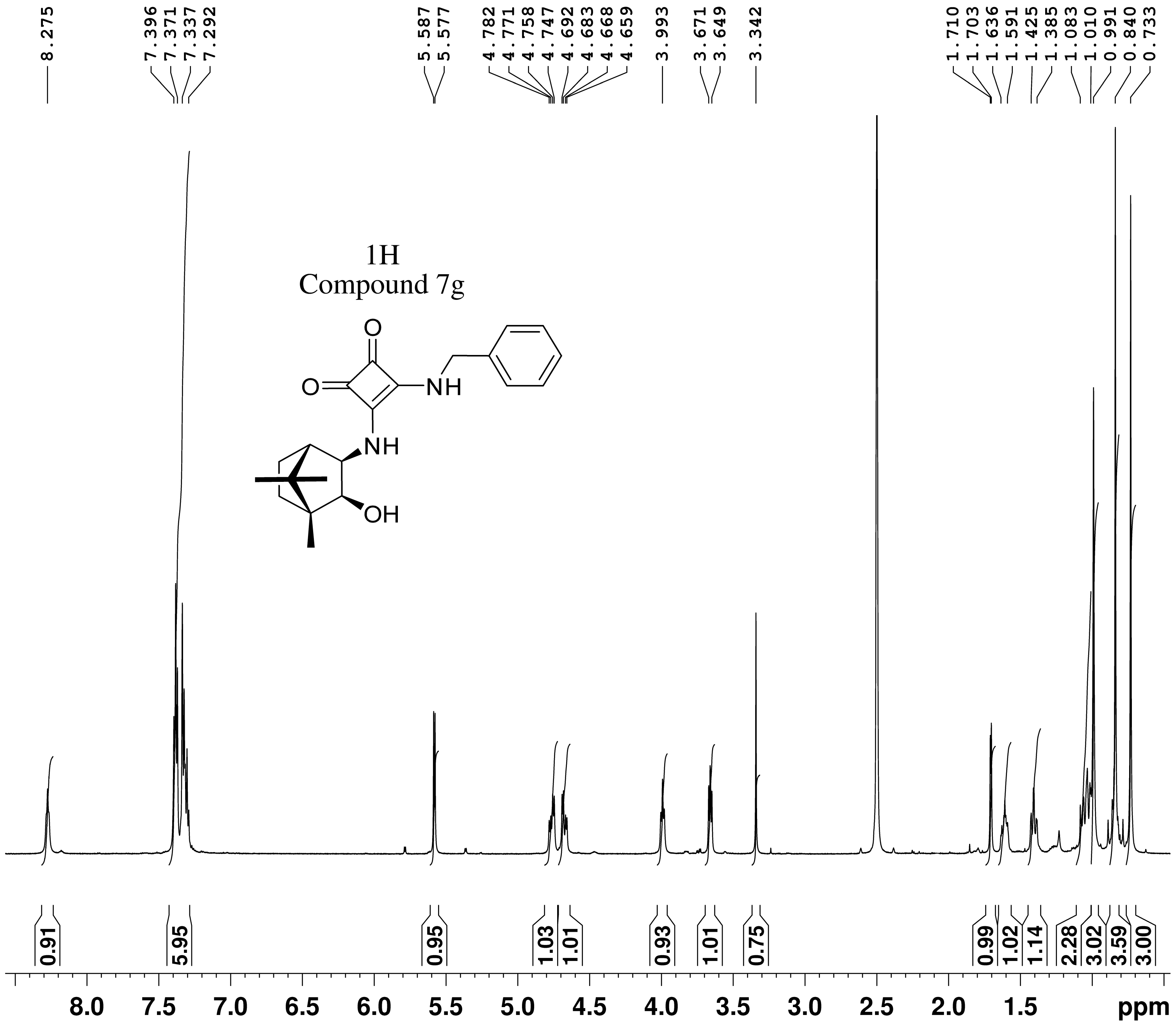

NAME	YNC00105B
EXPNO	11
PROCNO	1
Date_	20150721
Time	22.49
INSTRUM	spect
PROBHD	5 mm PABBO BB-
PULPROG	zg30
TD	32768
SOLVENT	DMSO
NS	16
DS	0
SWH	9615.385 Hz
FIDRES	0.293438 Hz
AQ	1.7039860 sec
RG	203
DW	52.000 usec
DE	13.95 usec
TE	293.0 K
D1	1.00000000 sec
TD0	1

======== CHANNEL f1 ========

SFO1	600.1145608 MHz
NUC1	1H
P1	10.85 usec
SI	65536
SF	600.1100048 MHz
WDW	EM
SSB	0
LB	0.00 Hz
GB	0
PC	1.00

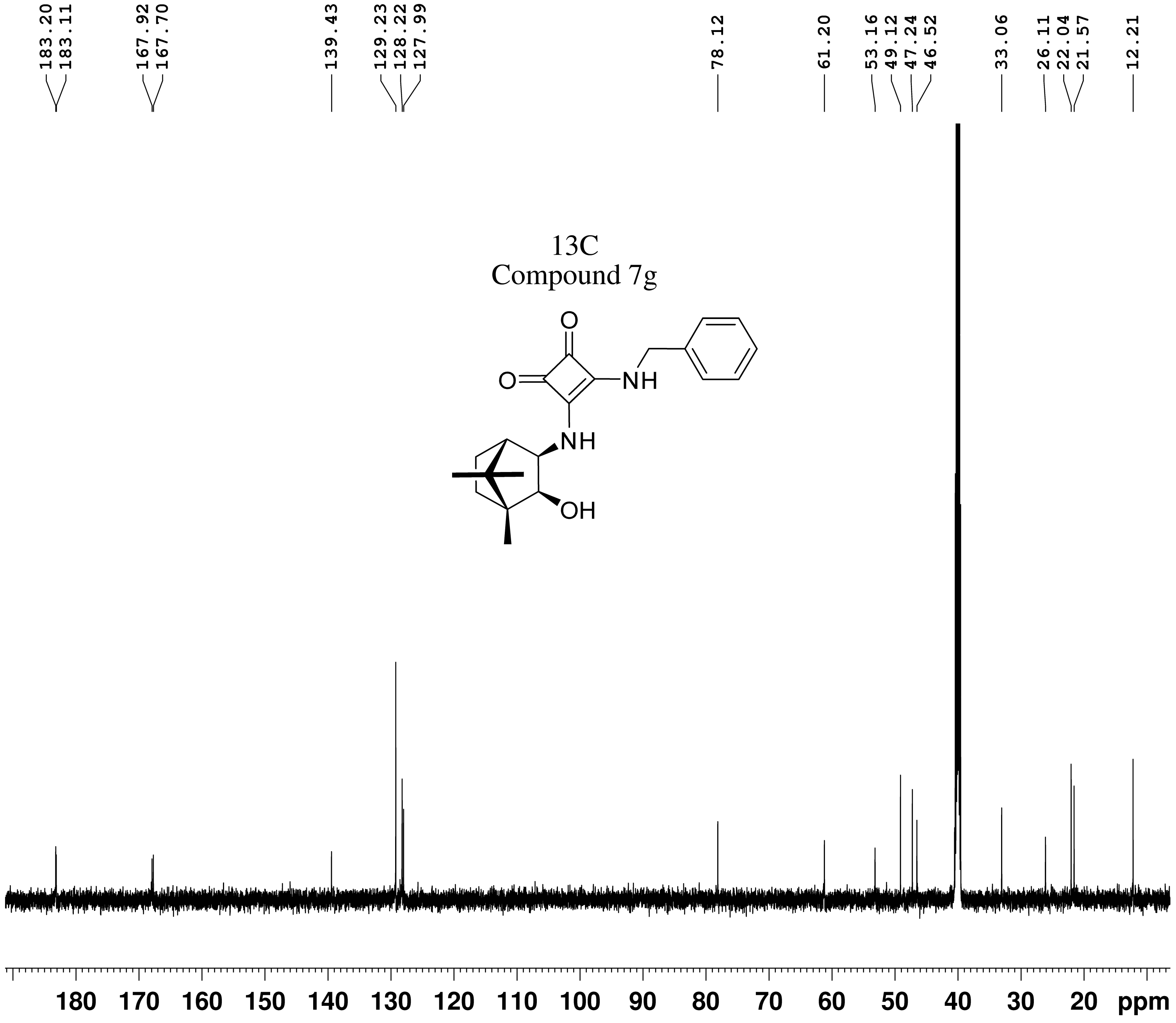

NAME	YNC00105B
EXPNO	12
PROCNO	1
Date_	20150721
Time	22.58
INSTRUM	spect
PROBHD	5 mm PABBO BB-
PULPROG	zgdc30
TD	32768
SOLVENT	DMSO
NS	256
DS	0
SWH	36057.691 Hz
FIDRES	1.100393 Hz
AQ	0.4544329 sec
RG	2050
DW	13.867 usec
DE	6.50 usec
TE	293.0 K
D1	1.50000000 sec
D11	0.03000000 sec
TD0	1

======== CHANNEL f1 ========

SFO1	150.9143788 MHz
NUC1	13C
P1	9.80 usec
SI	65536
SF	150.8977738 MHz
WDW	EM
SSB	0
LB	1.00 Hz
GB	0
PC	1.40

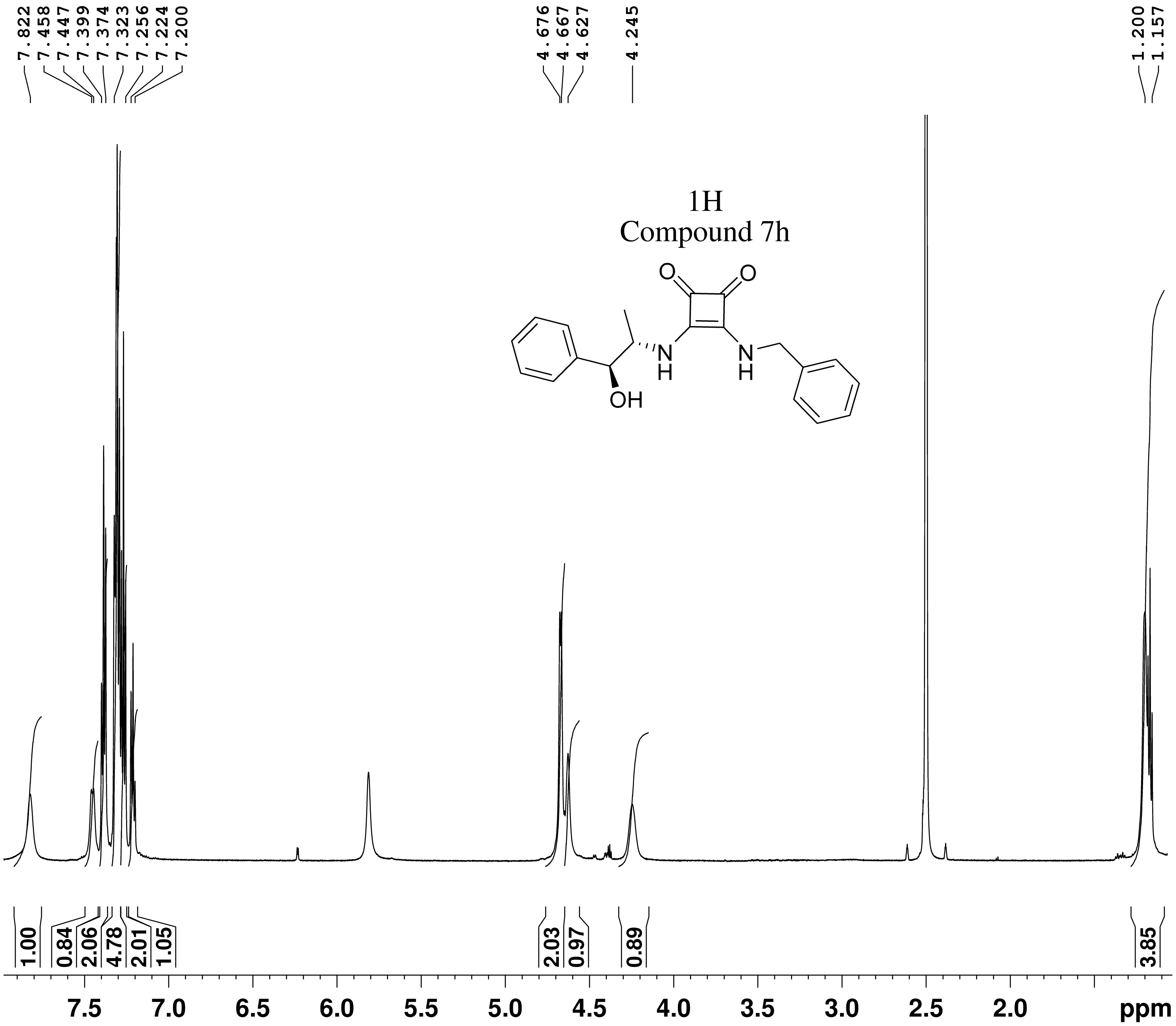

NAME	YNC01002
EXPNO	11
PROCNO	1
Date_	20140805
Time	23.06
INSTRUM	spect
PROBHD	5 mm PABBO BB-
PULPROG	zg30
TD	32768
SOLVENT	DMSO
NS	32
DS	0
SWH	9615.385 Hz
FIDRES	0.293438 Hz
AQ	1.7039860 sec
RG	144
DW	52.000 usec
DE	13.95 usec
TE	293.0 K
D1	1.00000000 sec
TD0	1

======== CHANNEL f1 ========

SFO1	600.1345610 MHz
NUC1	1H
P1	10.85 usec
SI	65536
SF	600.1300046 MHz
WDW	EM
SSB	0
LB	0.10 Hz
GB	0
PC	1.00

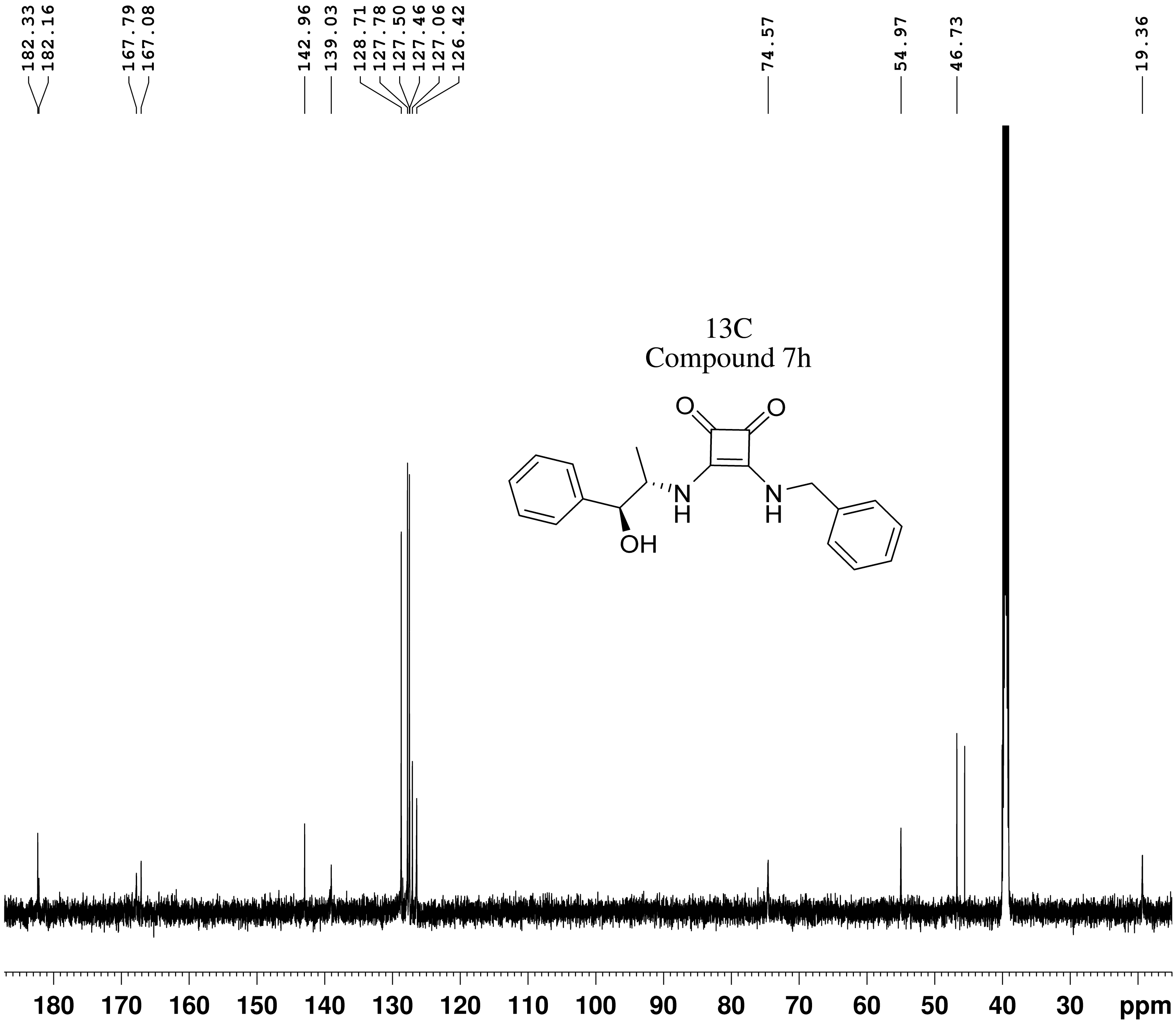

NAME	YNC01002
EXPNO	22
PROCNO	1
Date_	20150617
Time	21.04
INSTRUM	spect
PROBHD	5 mm PABBO BB-
PULPROG	zgdc30
TD	32768
SOLVENT	DMSO
NS	1024
DS	0
SWH	36057.691 Hz
FIDRES	1.100393 Hz
AQ	0.4544329 sec
RG	2050
DW	13.867 usec
DE	6.50 usec
TE	293.0 K
D1	1.50000000 sec
D11	0.03000000 sec
TD0	1
======== CHANNEL f1 ========	
SFO1	150.9143788 MHz
NUC1	13C
P1	9.80 usec
SI	65536
SF	150.8978451 MHz
WDW	EM
SSB	0
LB	1.00 Hz
GB	0
PC	1.00

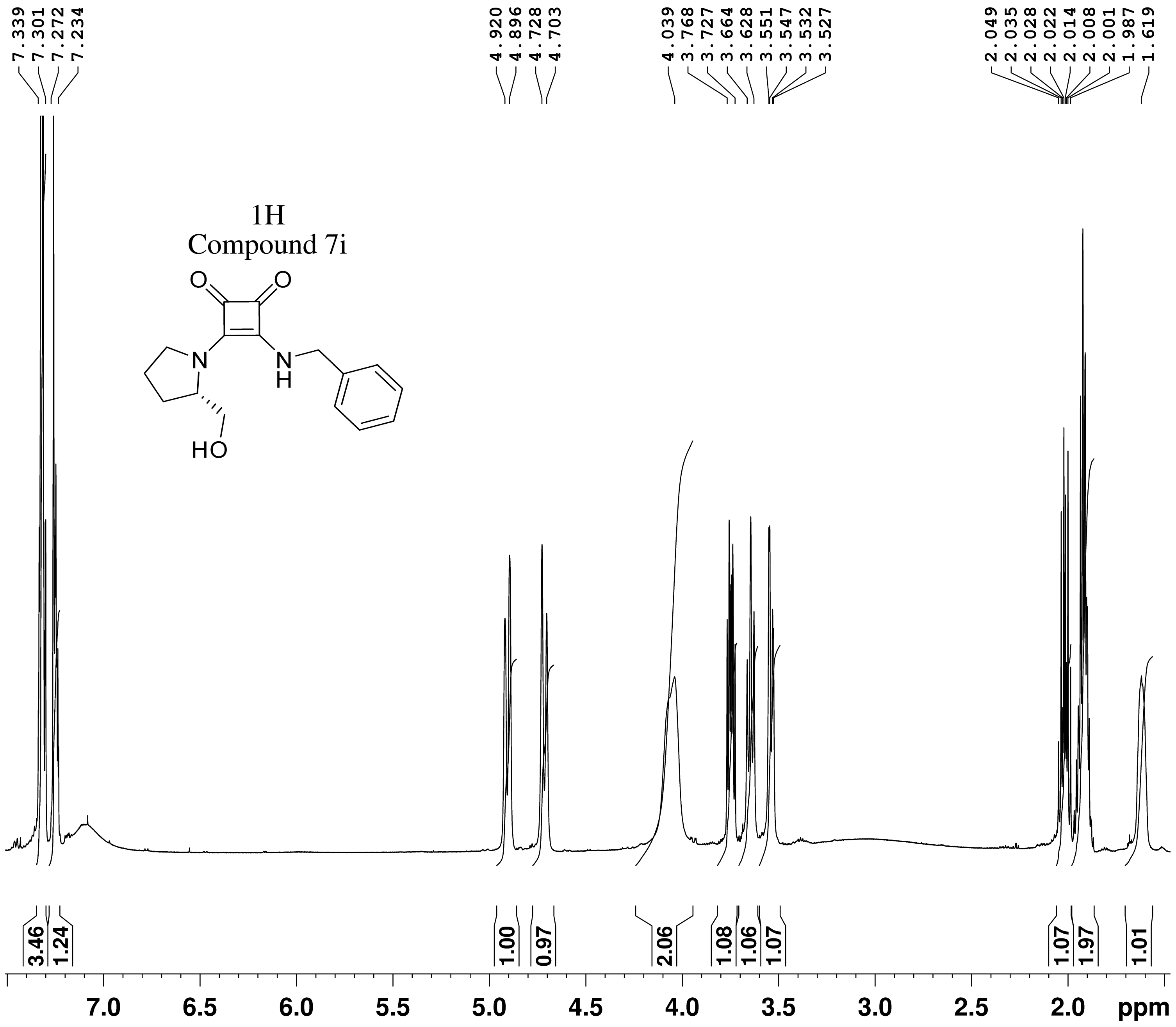

NAME	YNC03303B	
EXPNO	11	
PROCNO	1	
Date_	20150621	
Time	21.22	
INSTRUM	spect	
PROBHD	5 mm PABBO BB-	
PULPROG	zg30	
TD	32768	
SOLVENT	CDCl3	
NS	512	
DS	0	
SWH	9615.385	Hz
FIDRES	0.293438	Hz
AQ	1.7039860	sec
RG	228	
DW	52.000	usec
DE	13.95	usec
TE	323.0	K
D1	1.00000000	sec
TD0	1	

======== CHANNEL f1 ========

SFO1	600.1145608	MHz
NUC1	1H	
P1	10.85	usec
SI	65536	
SF	600.1100136	MHz
WDW	EM	
SSB	0	
LB	0.00	Hz
GB	0	
PC	1.00	

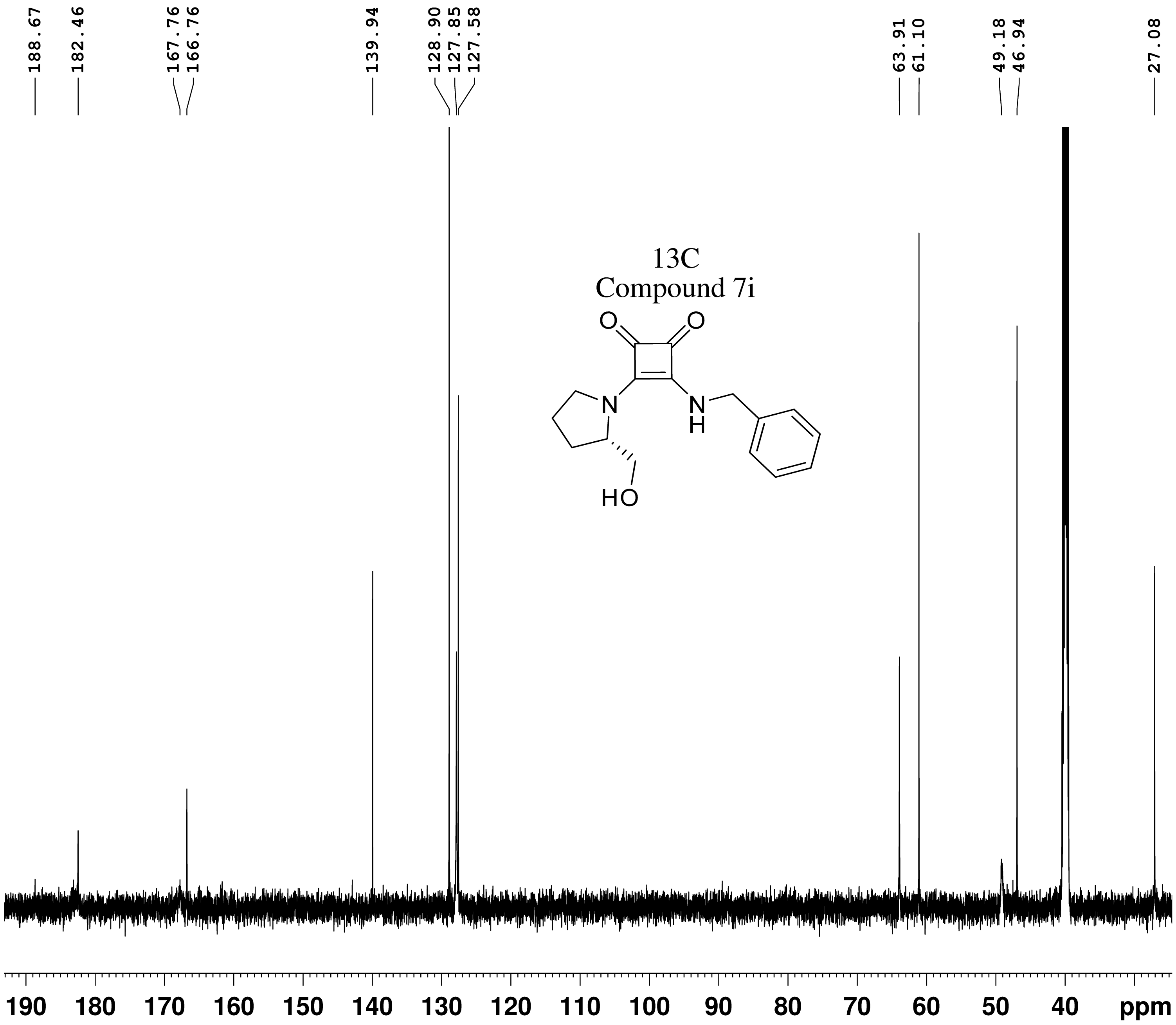

NAME	YNC03303A
EXPNO	12
PROCNO	1
Date_	20141121
Time	20.57
INSTRUM	spect
PROBHD	5 mm PABBO BB-
PULPROG	zgdc30
TD	32768
SOLVENT	DMSO
NS	1024
DS	0
SWH	36057.691 Hz
FIDRES	1.100393 Hz
AQ	0.4544329 sec
RG	2050
DW	13.867 usec
DE	7.68 usec
TE	293.0 K
D1	1.50000000 sec
D11	0.03000000 sec
TD0	1

======== CHANNEL f1 ========

SFO1	150.9188042 MHz
NUC1	13C
P1	9.80 usec
SI	65536
SF	150.9028150 MHz
WDW	EM
SSB	0
LB	1.00 Hz
GB	0
PC	1.00

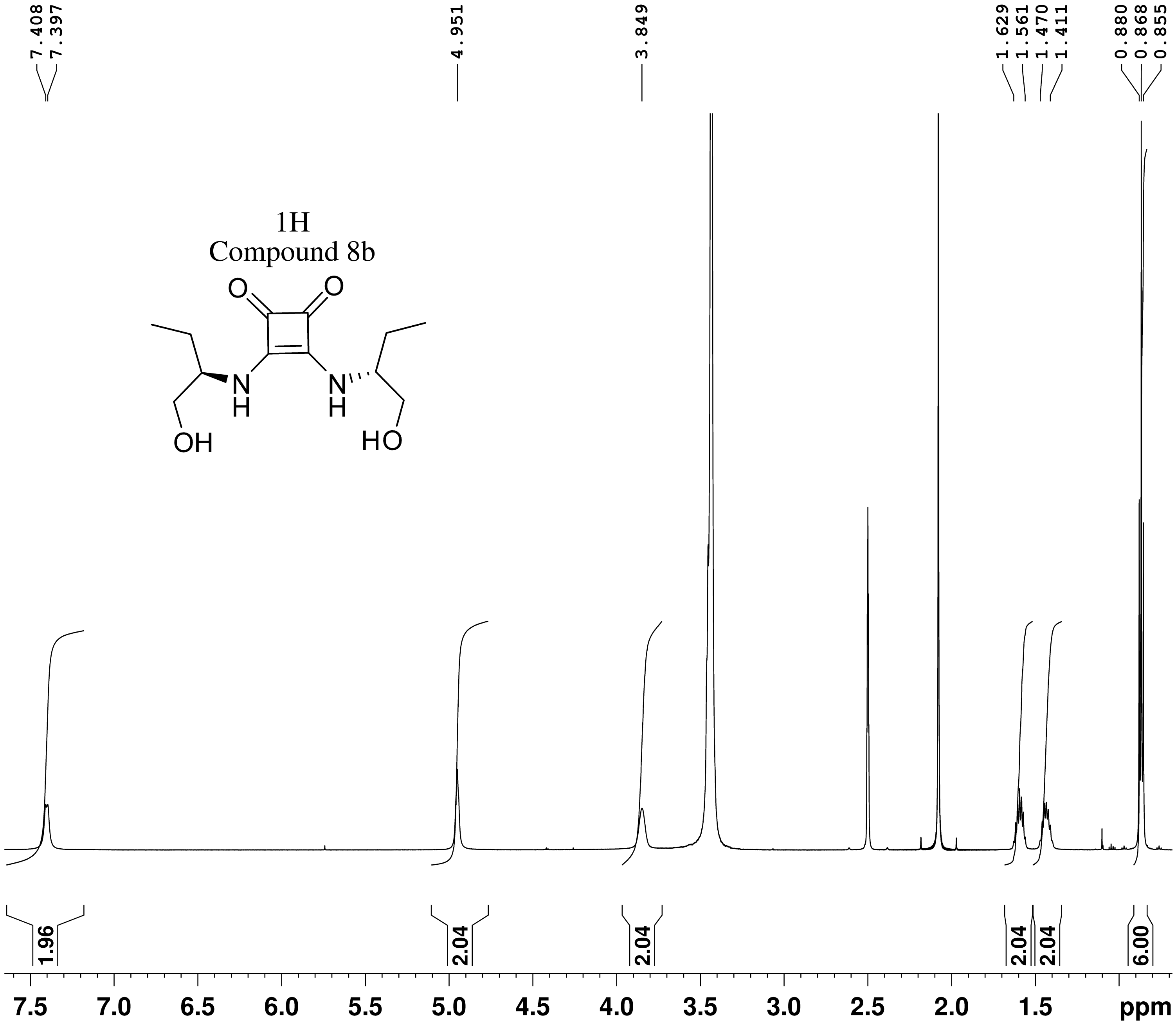

NAME	DL-172
EXPNO	11
PROCNO	1
Date_	20140129
Time	20.32
INSTRUM	spect
PROBHD	5 mm PABBO BB-
PULPROG	zg30
TD	32768
SOLVENT	DMSO
NS	32
DS	0
SWH	9615.385 Hz
FIDRES	0.293438 Hz
AQ	1.7039860 sec
RG	40.3
DW	52.000 usec
DE	13.95 usec
TE	293.0 K
D1	1.00000000 sec
TD0	1

======== CHANNEL f1 ========

SFO1	600.1345610 MHz
NUC1	1H
P1	10.85 usec
SI	65536
SF	600.1300075 MHz
WDW	EM
SSB	0
LB	0.10 Hz
GB	0
PC	1.00

182.12
167.87
63.29
56.88
25.04
10.32

13C
Compound 8b

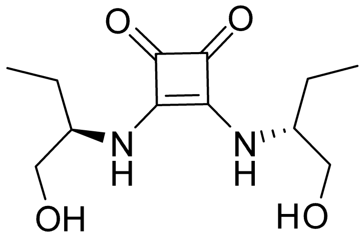

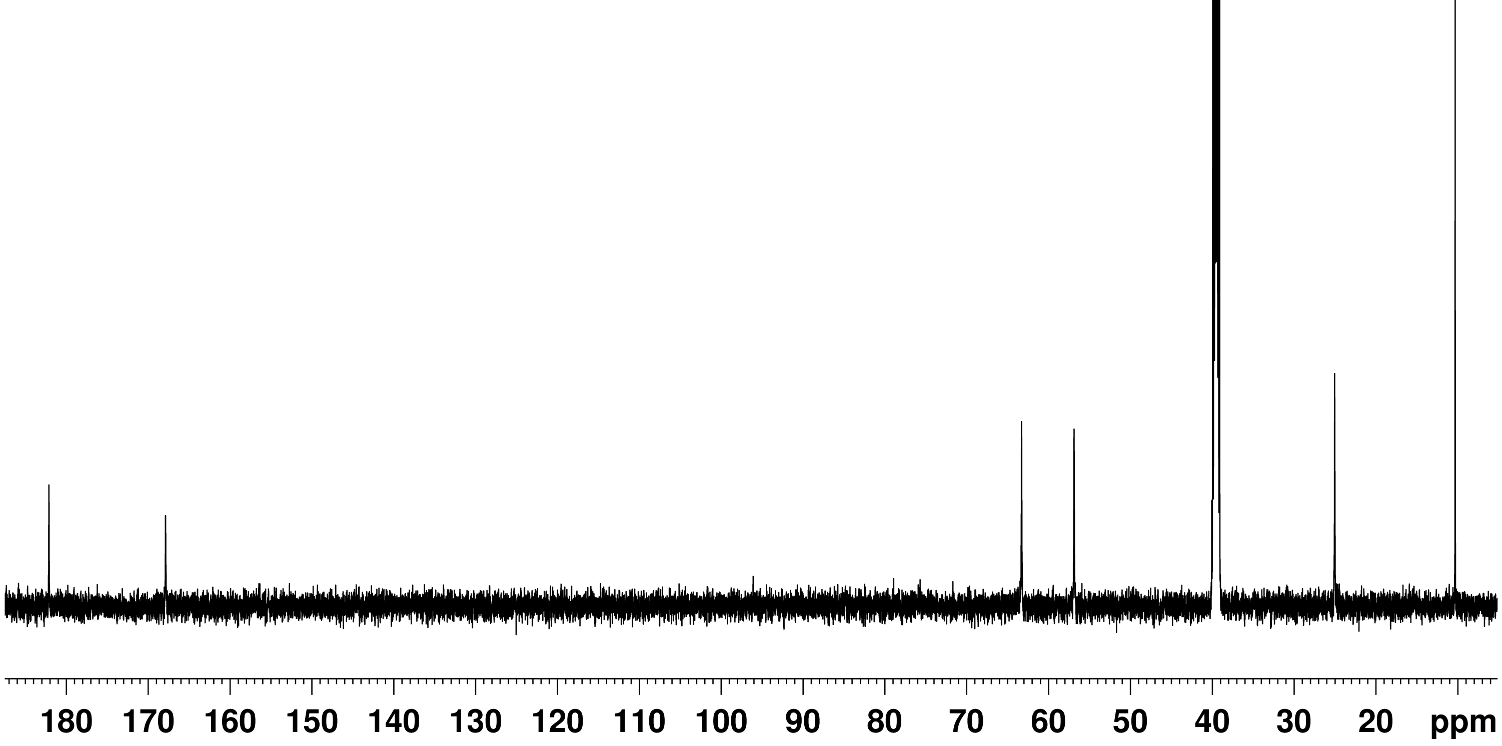

NAME	DL-172
EXPNO	12
PROCNO	1
Date_	20140129
Time	21.06
INSTRUM	spect
PROBHD	5 mm PABBO BB-
PULPROG	zgdc30
TD	32768
SOLVENT	DMSO
NS	1024
DS	0
SWH	36057.691 Hz
FIDRES	1.100393 Hz
AQ	0.4544329 sec
RG	2050
DW	13.867 usec
DE	7.48 usec
TE	293.0 K
D1	1.50000000 sec
D11	0.03000000 sec
TD0	1

======== CHANNEL f1 ========

SFO1	150.9188042 MHz
NUC1	13C
P1	10.75 usec
SI	65536
SF	150.9028611 MHz
WDW	EM
SSB	0
LB	1.00 Hz
GB	0
PC	1.00

1H
Compound 8e

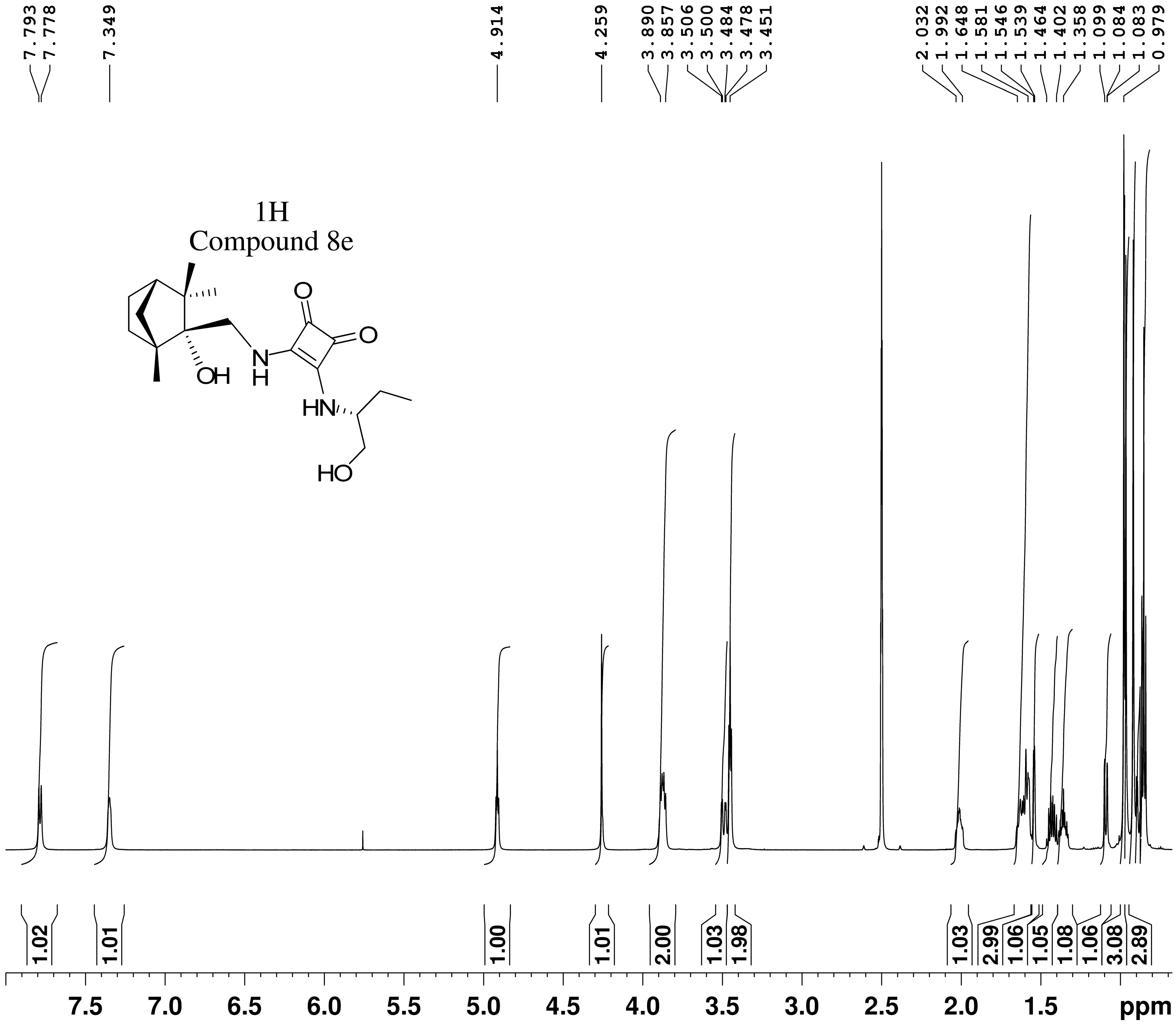

NAME	YNC04603A
EXPNO	11
PROCNO	1
Date_	20141209
Time	3.51
INSTRUM	spect
PROBHD	5 mm PABBO BB-
PULPROG	zg30
TD	32768
SOLVENT	DMSO
NS	32
DS	0
SWH	9615.385 Hz
FIDRES	0.293438 Hz
AQ	1.7039860 sec
RG	144
DW	52.000 usec
DE	13.95 usec
TE	293.0 K
D1	1.00000000 sec
TD0	1

======== CHANNEL f1 ========

SFO1	600.1345610 MHz
NUC1	1H
P1	10.85 usec
SI	65536
SF	600.1300059 MHz
WDW	EM
SSB	0
LB	0.10 Hz
GB	0
PC	1.00

13C
Compound 8e

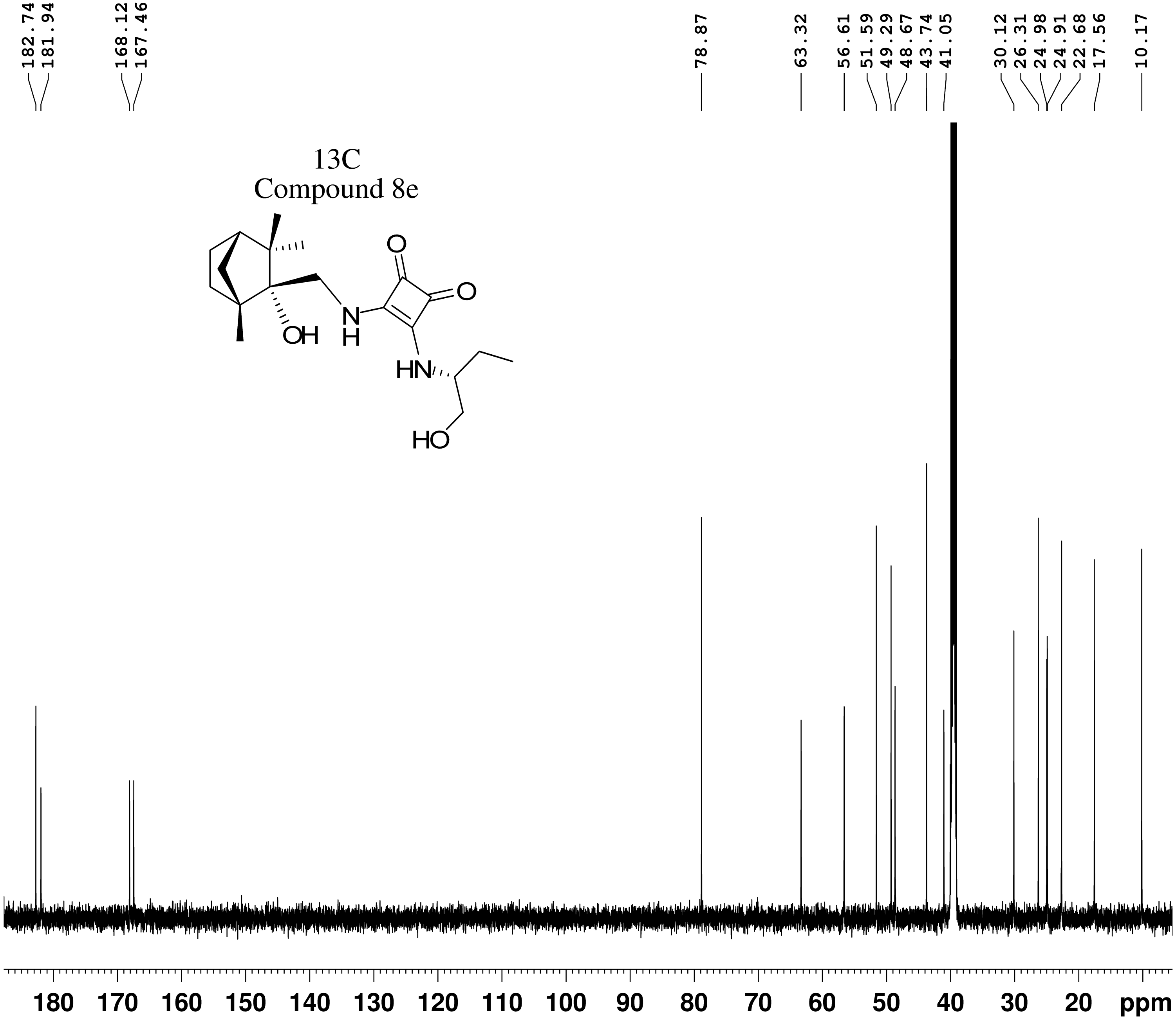

NAME	YNC04603A
EXPNO	12
PROCNO	1
Date_	20141209
Time	4.25
INSTRUM	spect
PROBHD	5 mm PABBO BB-
PULPROG	zgdc30
TD	32768
SOLVENT	DMSO
NS	1024
DS	0
SWH	36057.691 Hz
FIDRES	1.100393 Hz
AQ	0.4544329 sec
RG	2050
DW	13.867 usec
DE	7.68 usec
TE	293.0 K
D1	1.50000000 sec
D11	0.03000000 sec
TD0	1

======== CHANNEL f1 ========

SFO1	150.9188042 MHz
NUC1	13C
P1	9.80 usec
SI	65536
SF	150.9028749 MHz
WDW	EM
SSB	0
LB	1.00 Hz
GB	0
PC	1.00

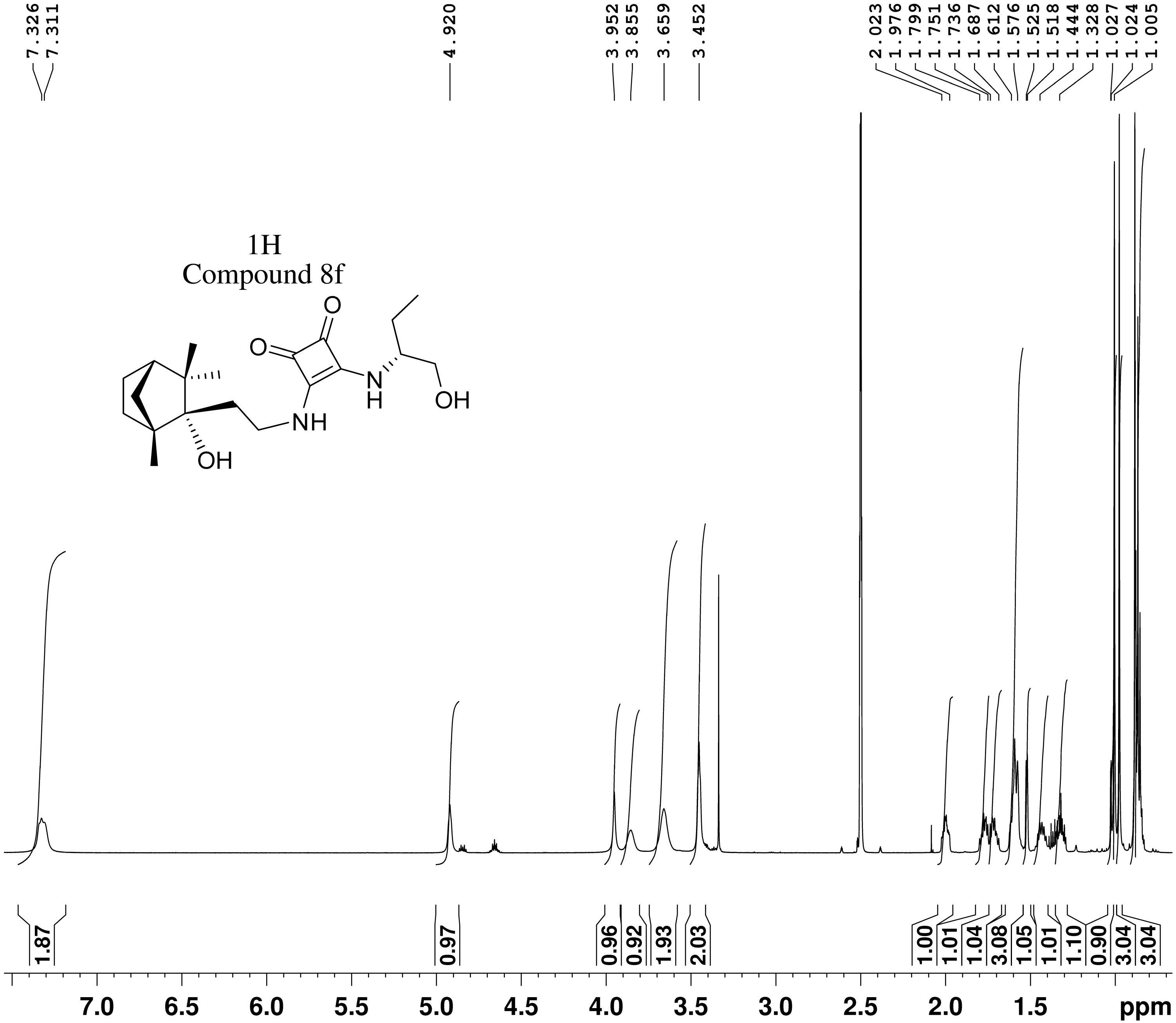

NAME	NB-82A
EXPNO	11
PROCNO	1
Date_	20140703
Time	17.57
INSTRUM	spect
PROBHD	5 mm PABBO BB-
PULPROG	zg30
TD	32768
SOLVENT	DMSO
NS	32
DS	0
SWH	9615.385 Hz
FIDRES	0.293438 Hz
AQ	1.7039860 sec
RG	144
DW	52.000 usec
DE	13.95 usec
TE	293.0 K
D1	1.00000000 sec
TD0	1

======== CHANNEL f1 ========

SFO1	600.1345610 MHz
NUC1	1H
P1	10.85 usec
SI	65536
SF	600.1300047 MHz
WDW	EM
SSB	0
LB	0.10 Hz
GB	0
PC	1.00

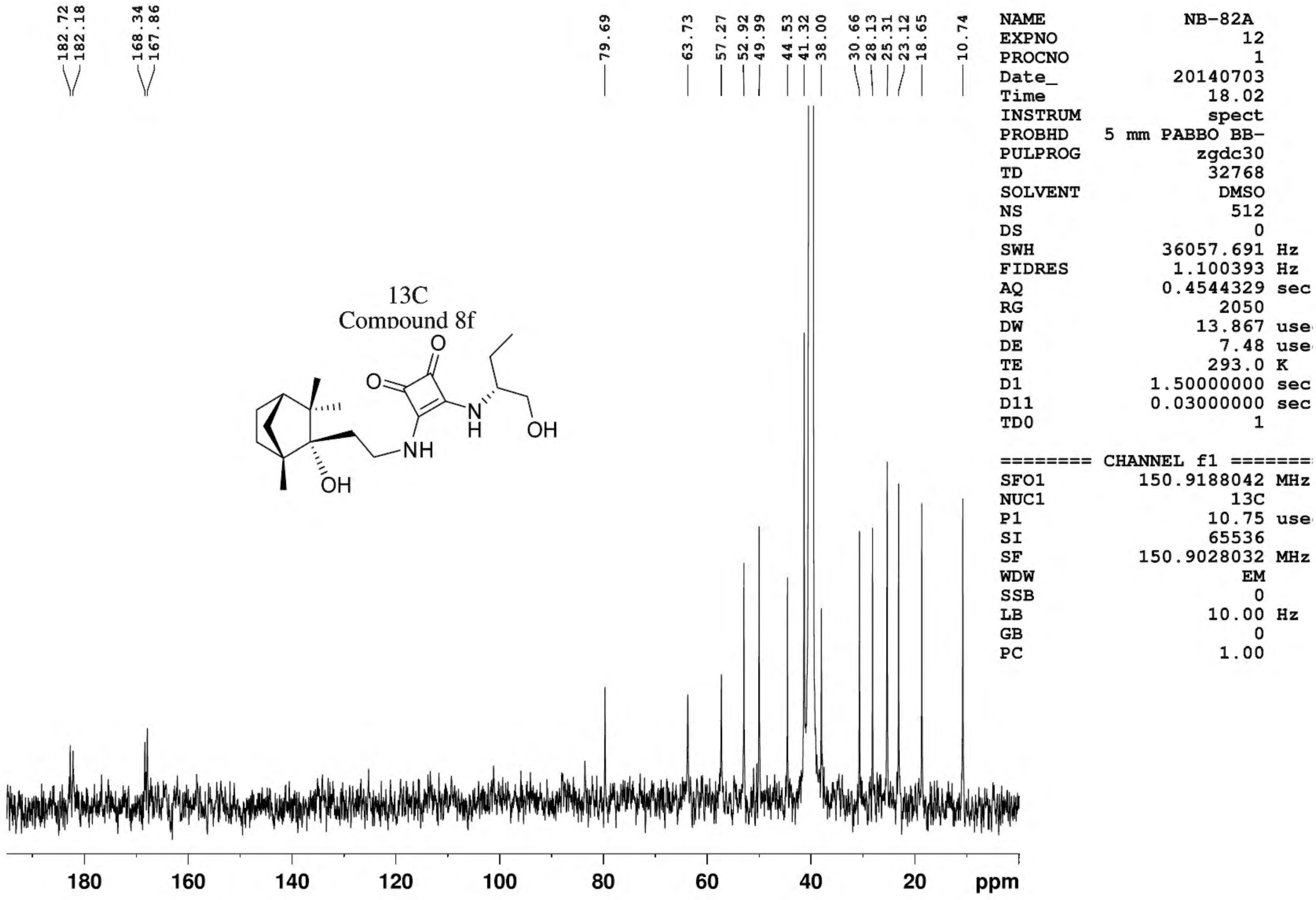
182.72
182.18
168.34
167.86
79.69
63.73
57.27
52.92
49.99
44.53
41.32
38.00
30.66
28.13
25.31
23.12
18.65
10.74
13C
Compound 8f
O
O
N
H
OH
NH
OH
NAME NB-82A
EXPNO 12
PROCNO 1
Date_ 20140703
Time 18.02
INSTRUM spect
PROBHD 5 mm PABBO BB-
PULPROG zgdc30
TD 32768
SOLVENT DMSO
NS 512
DS 0
SWH 36057.691 Hz
FIDRES 1.100393 Hz
AQ 0.4544329 sec
RG 2050
DW 13.867 use
DE 7.48 use
TE 293.0 K
D1 1.50000000 sec
D11 0.03000000 sec
TD0 1
======== CHANNEL f1 =======
SFO1 150.9188042 MHz
NUC1 13C
P1 10.75 use
SI 65536
SF 150.9028032 MHz
WDW EM
SSB 0
LB 10.00 Hz
GB 0
PC 1.00
180
160
140
120
100
80
60
40
20
ppm

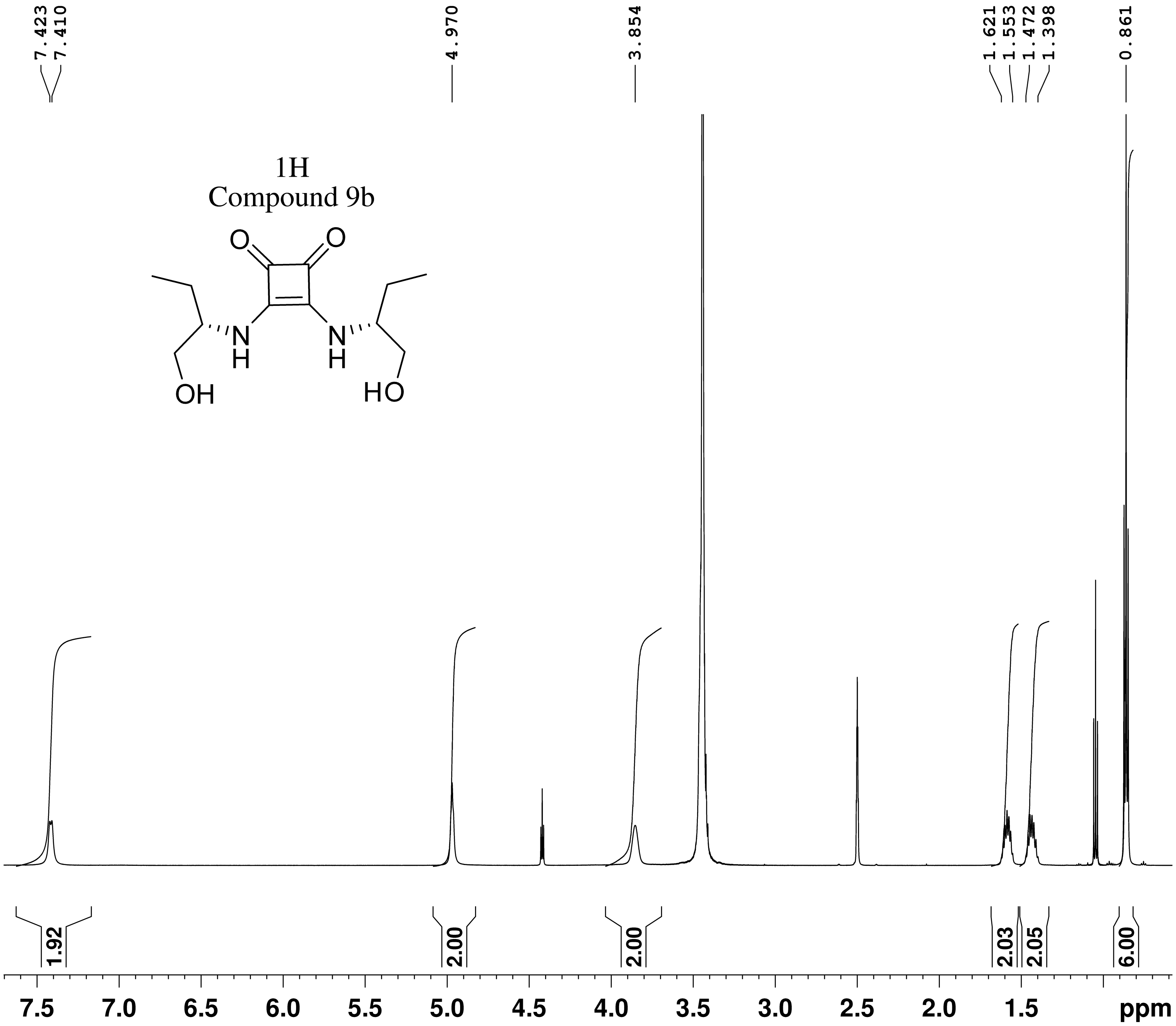

NAME	DL-176	
EXPNO	11	
PROCNO	1	
Date_	20140212	
Time	17.14	
INSTRUM	spect	
PROBHD	5 mm PABBO BB-	
PULPROG	zg30	
TD	32768	
SOLVENT	DMSO	
NS	16	
DS	0	
SWH	9615.385	Hz
FIDRES	0.293438	Hz
AQ	1.7039860	sec
RG	36	
DW	52.000	usec
DE	13.95	usec
TE	293.0	K
D1	1.00000000	sec
TD0	1	

======== CHANNEL f1 ========

SFO1	600.1345610	MHz
NUC1	1H	
P1	10.85	usec
SI	65536	
SF	600.1300077	MHz
WDW	EM	
SSB	0	
LB	0.10	Hz
GB	0	
PC	1.00	

13C
Compound 9b

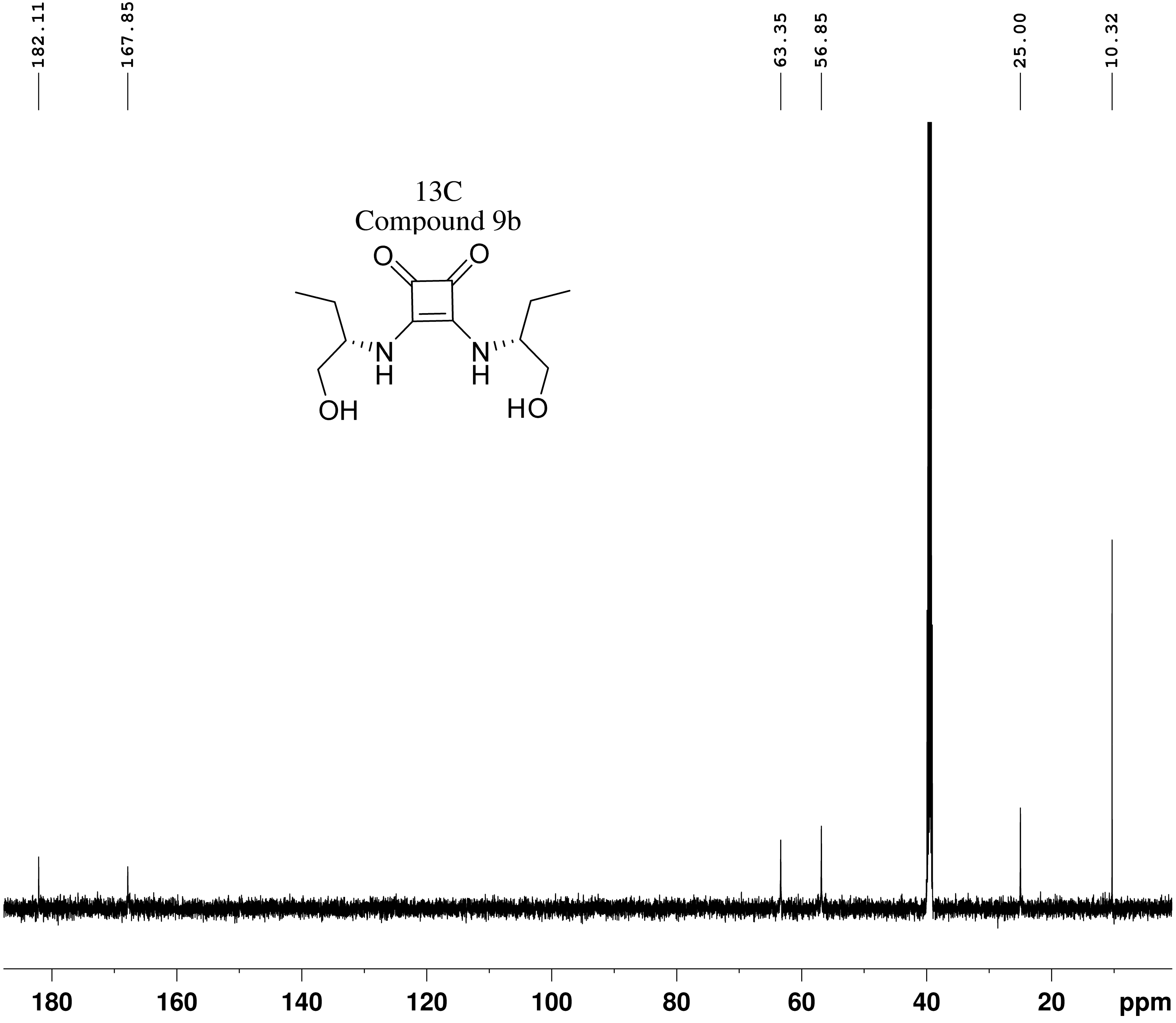

NAME	DL-176	
EXPNO	12	
PROCNO	1	
Date_	20140212	
Time	17.19	
INSTRUM	spect	
PROBHD	5 mm PABBO BB-	
PULPROG	zgdc30	
TD	32768	
SOLVENT	DMSO	
NS	128	
DS	0	
SWH	36057.691	Hz
FIDRES	1.100393	Hz
AQ	0.4544329	sec
RG	2050	
DW	13.867	usec
DE	7.48	usec
TE	293.1	K
D1	1.50000000	sec
D11	0.03000000	sec
TD0	1	
======== CHANNEL f1 ========		
SFO1	150.9188042	MHz
NUC1	13C	
P1	10.75	usec
SI	65536	
SF	150.9028600	MHz
WDW	EM	
SSB	0	
LB	1.00	Hz
GB	0	
PC	1.00	

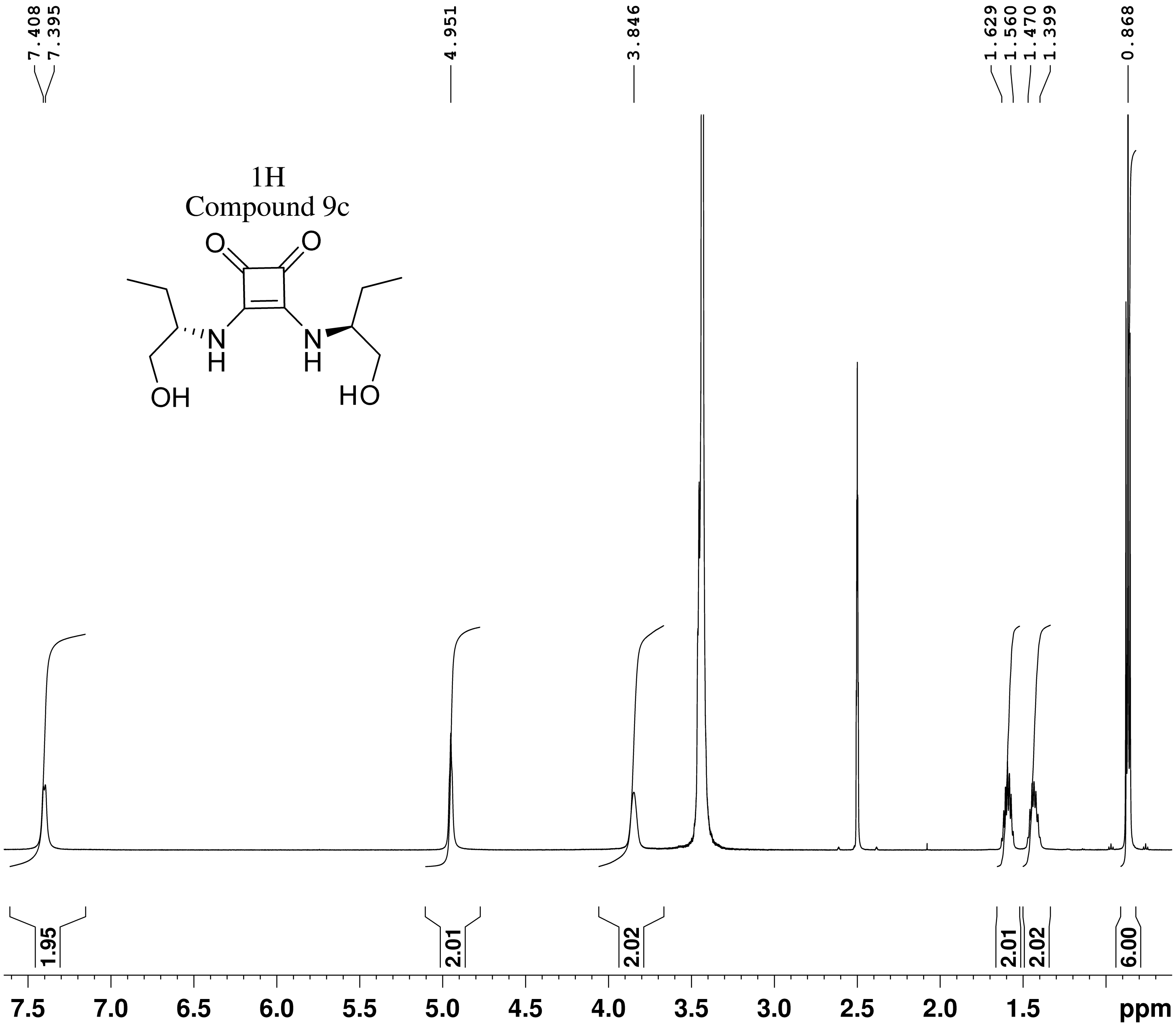

NAME	DL-173	
EXPNO	11	
PROCNO	1	
Date_	20140131	
Time	17.48	
INSTRUM	spect	
PROBHD	5 mm PABBO BB-	
PULPROG	zg30	
TD	32768	
SOLVENT	DMSO	
NS	16	
DS	0	
SWH	9615.385	Hz
FIDRES	0.293438	Hz
AQ	1.7039860	sec
RG	36	
DW	52.000	usec
DE	13.95	usec
TE	293.0	K
D1	1.00000000	sec
TD0	1	

======== CHANNEL f1 ========

SFO1	600.1345610	MHz
NUC1	1H	
P1	10.85	usec
SI	65536	
SF	600.1300074	MHz
WDW	EM	
SSB	0	
LB	0.10	Hz
GB	0	
PC	1.00	

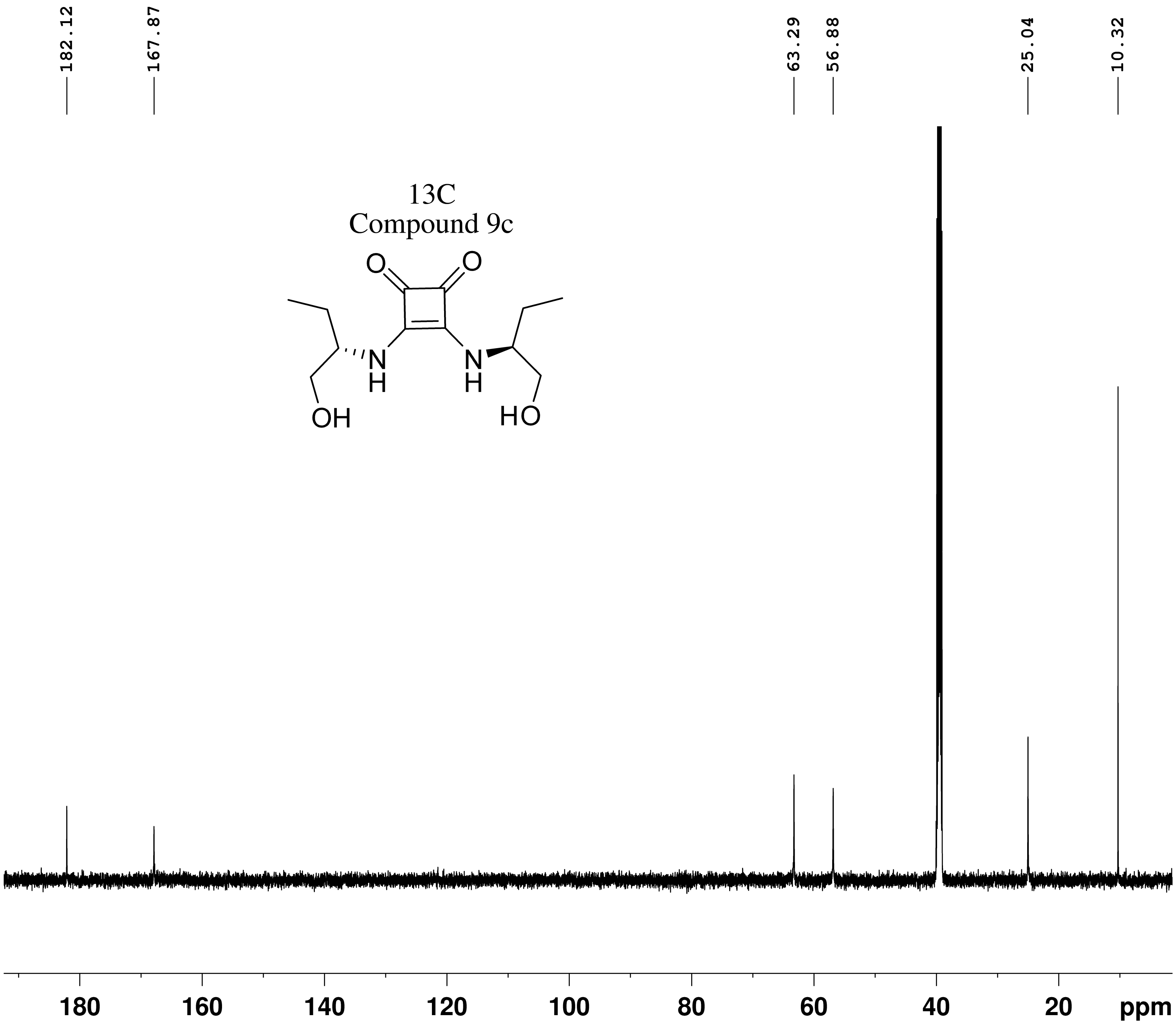

NAME	DL-173
EXPNO	12
PROCNO	1
Date_	20140131
Time	18.23
INSTRUM	spect
PROBHD	5 mm PABBO BB-
PULPROG	zgdc30
TD	32768
SOLVENT	DMSO
NS	1024
DS	0
SWH	36057.691 Hz
FIDRES	1.100393 Hz
AQ	0.4544329 sec
RG	2050
DW	13.867 usec
DE	7.48 usec
TE	293.0 K
D1	1.50000000 sec
D11	0.03000000 sec
TD0	1

======== CHANNEL f1 ========

SFO1	150.9188042 MHz
NUC1	13C
P1	10.75 usec
SI	65536
SF	150.9028613 MHz
WDW	EM
SSB	0
LB	1.00 Hz
GB	0
PC	1.00

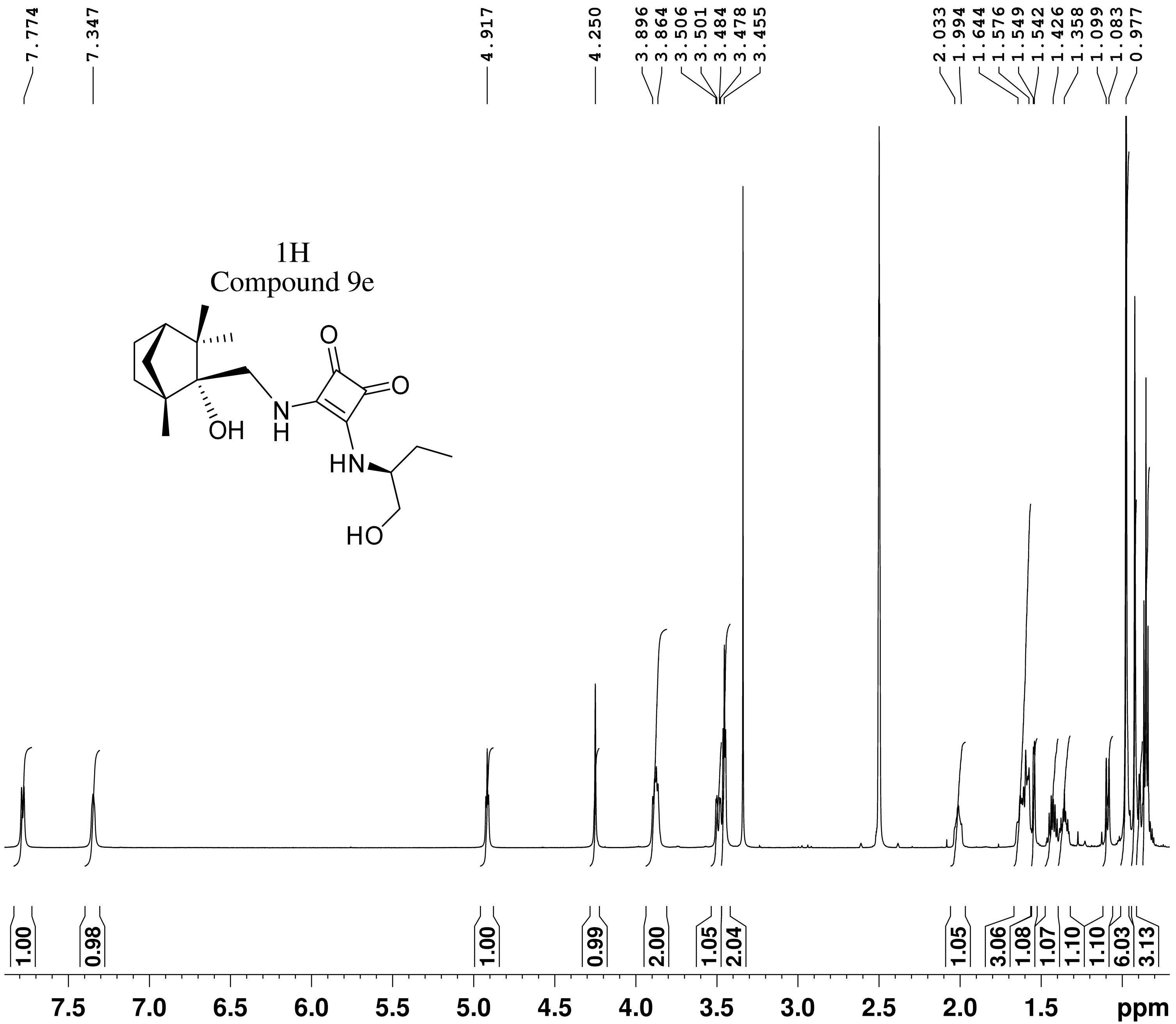

NAME	YNC04103A
EXPNO	11
PROCNO	1
Date_	20141113
Time	19.15
INSTRUM	spect
PROBHD	5 mm PABBO BB-
PULPROG	zg30
TD	32768
SOLVENT	DMSO
NS	32
DS	0
SWH	9615.385 Hz
FIDRES	0.293438 Hz
AQ	1.7039860 sec
RG	144
DW	52.000 usec
DE	13.95 usec
TE	293.0 K
D1	1.00000000 sec
TD0	1

======== CHANNEL f1 ========

SFO1	600.1345610 MHz
NUC1	1H
P1	10.85 usec
SI	65536
SF	600.1300046 MHz
WDW	EM
SSB	0
LB	0.10 Hz
GB	0
PC	1.00

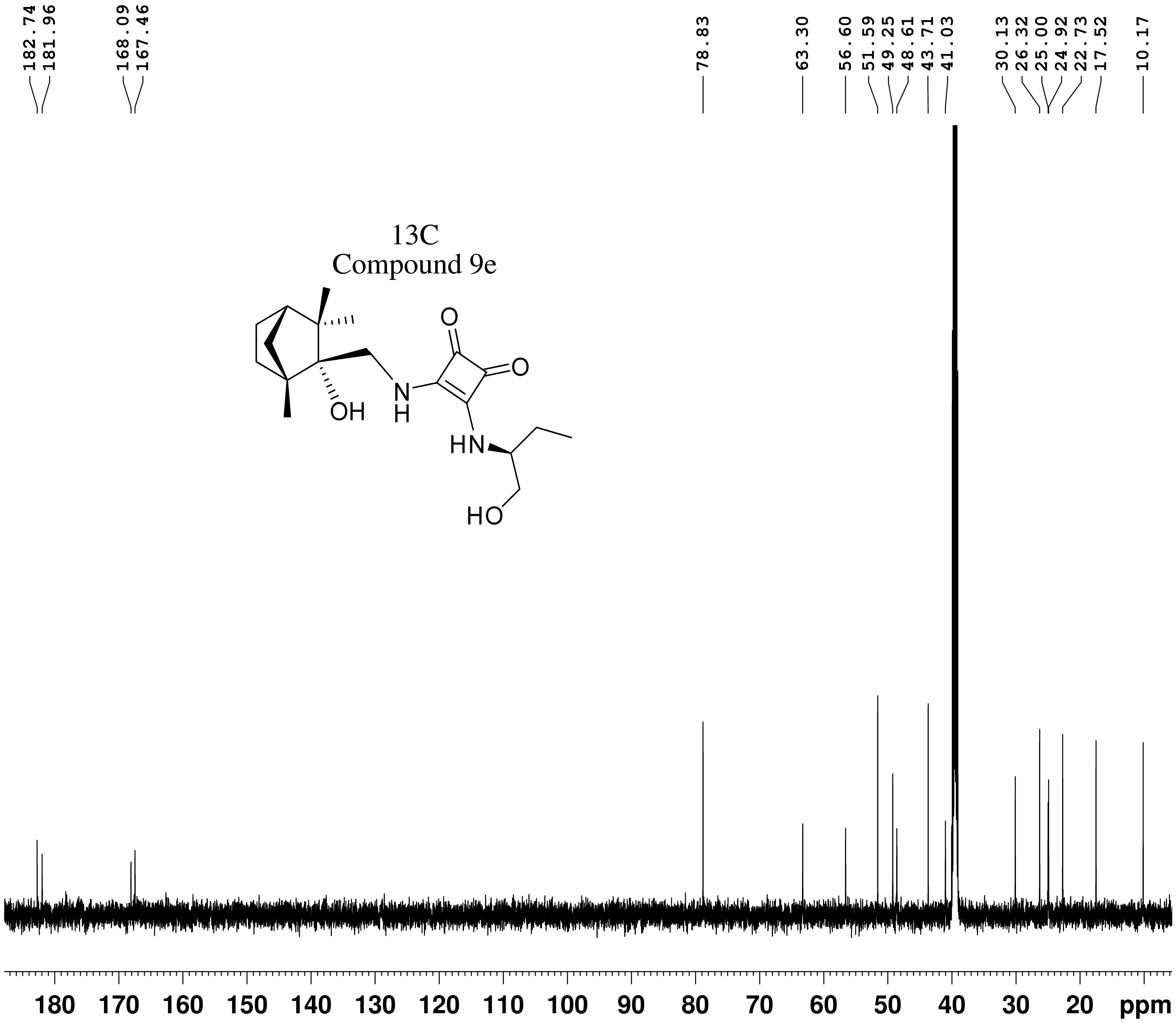

13C
Compound 9e

NAME	YNC04103A	
EXPNO	12	
PROCNO	1	
Date_	20141113	
Time	19.24	
INSTRUM	spect	
PROBHD	5 mm PABBO BB-	
PULPROG	zgdc30	
TD	32768	
SOLVENT	DMSO	
NS	256	
DS	0	
SWH	36057.691	Hz
FIDRES	1.100393	Hz
AQ	0.4544329	sec
RG	2050	
DW	13.867	usec
DE	7.68	usec
TE	293.0	K
D1	1.50000000	sec
D11	0.03000000	sec
TD0	1	

======== CHANNEL f1 ========

SFO1	150.9188042	MHz
NUC1	13C	
P1	9.80	usec
SI	65536	
SF	150.9028751	MHz
WDW	EM	
SSB	0	
LB	1.00	Hz
GB	0	
PC	1.00	

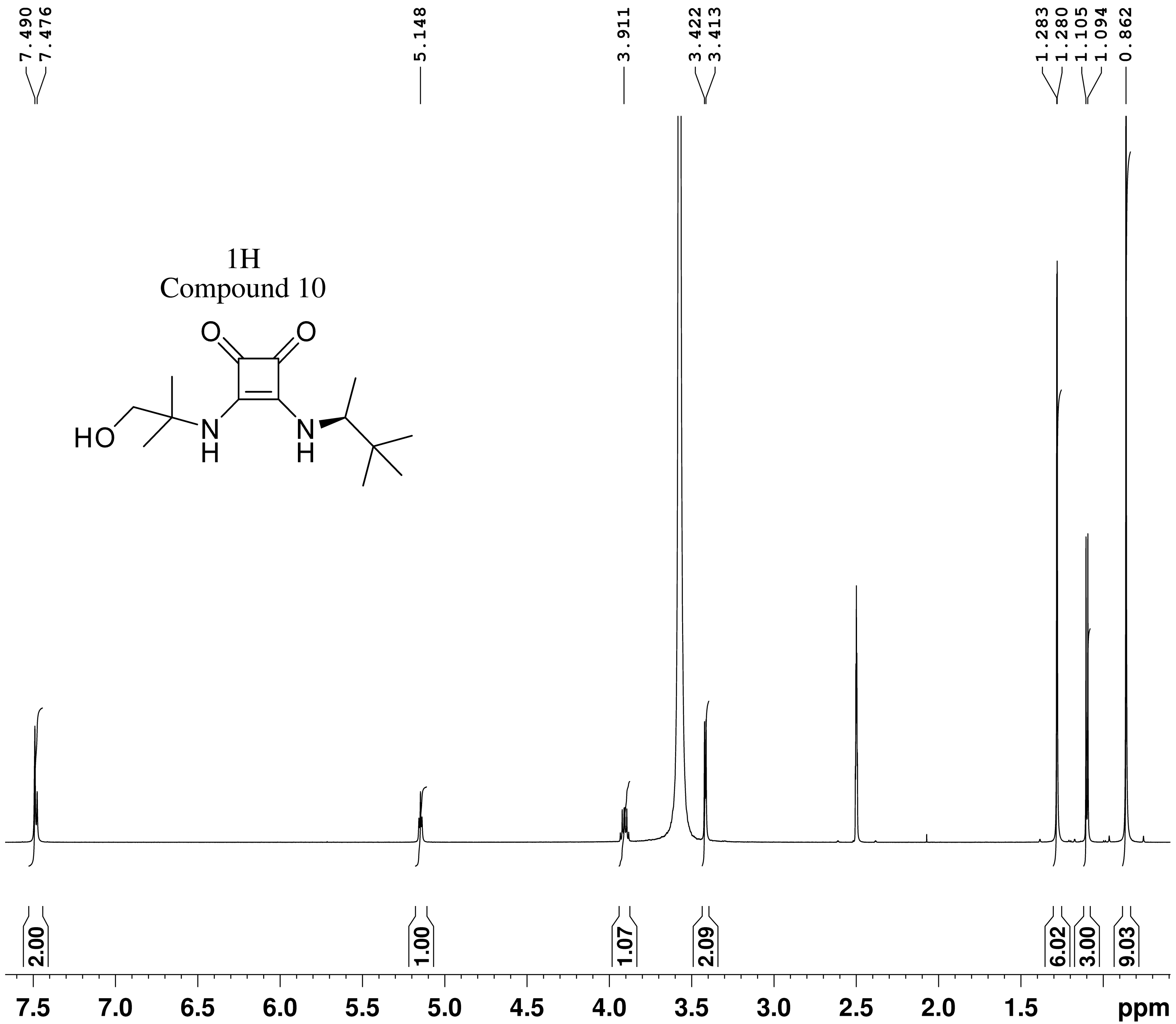

NAME	NB_081_03A
EXPNO	11
PROCNO	1
Date_	20140627
Time	21.18
INSTRUM	spect
PROBHD	5 mm PABBO BB-
PULPROG	zg30
TD	32768
SOLVENT	DMSO
NS	32
DS	0
SWH	9615.385 Hz
FIDRES	0.293438 Hz
AQ	1.7039860 sec
RG	45.2
DW	52.000 usec
DE	13.95 usec
TE	293.0 K
D1	1.00000000 sec
TD0	1

======== CHANNEL f1 ========

SFO1	600.1345610 MHz
NUC1	1H
P1	10.85 usec
SI	65536
SF	600.1300050 MHz
WDW	EM
SSB	0
LB	0.10 Hz
GB	0
PC	1.00

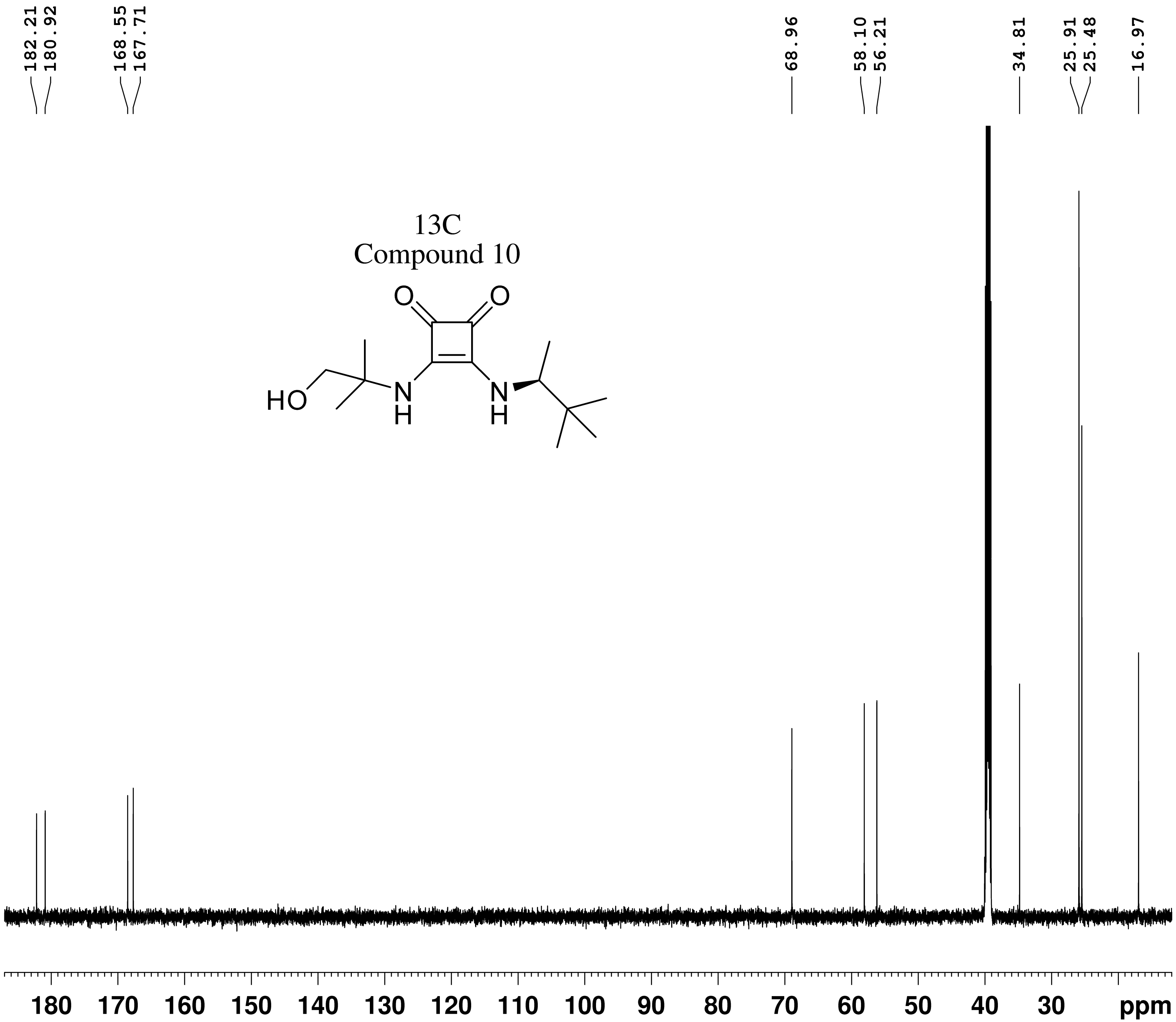

NAME	NB_081_03A	
EXPNO	12	
PROCNO	1	
Date_	20140627	
Time	21.52	
INSTRUM	spect	
PROBHD	5 mm PABBO BB-	
PULPROG	zgdc30	
TD	32768	
SOLVENT	DMSO	
NS	1024	
DS	0	
SWH	36057.691	Hz
FIDRES	1.100393	Hz
AQ	0.4544329	sec
RG	2050	
DW	13.867	usec
DE	7.48	usec
TE	293.0	K
D1	1.50000000	sec
D11	0.03000000	sec
TD0	1	
========	CHANNEL f1 ========	
SFO1	150.9188042	MHz
NUC1	13C	
P1	10.75	usec
SI	65536	
SF	150.9028386	MHz
WDW	EM	
SSB	0	
LB	1.00	Hz
GB	0	
PC	1.00	

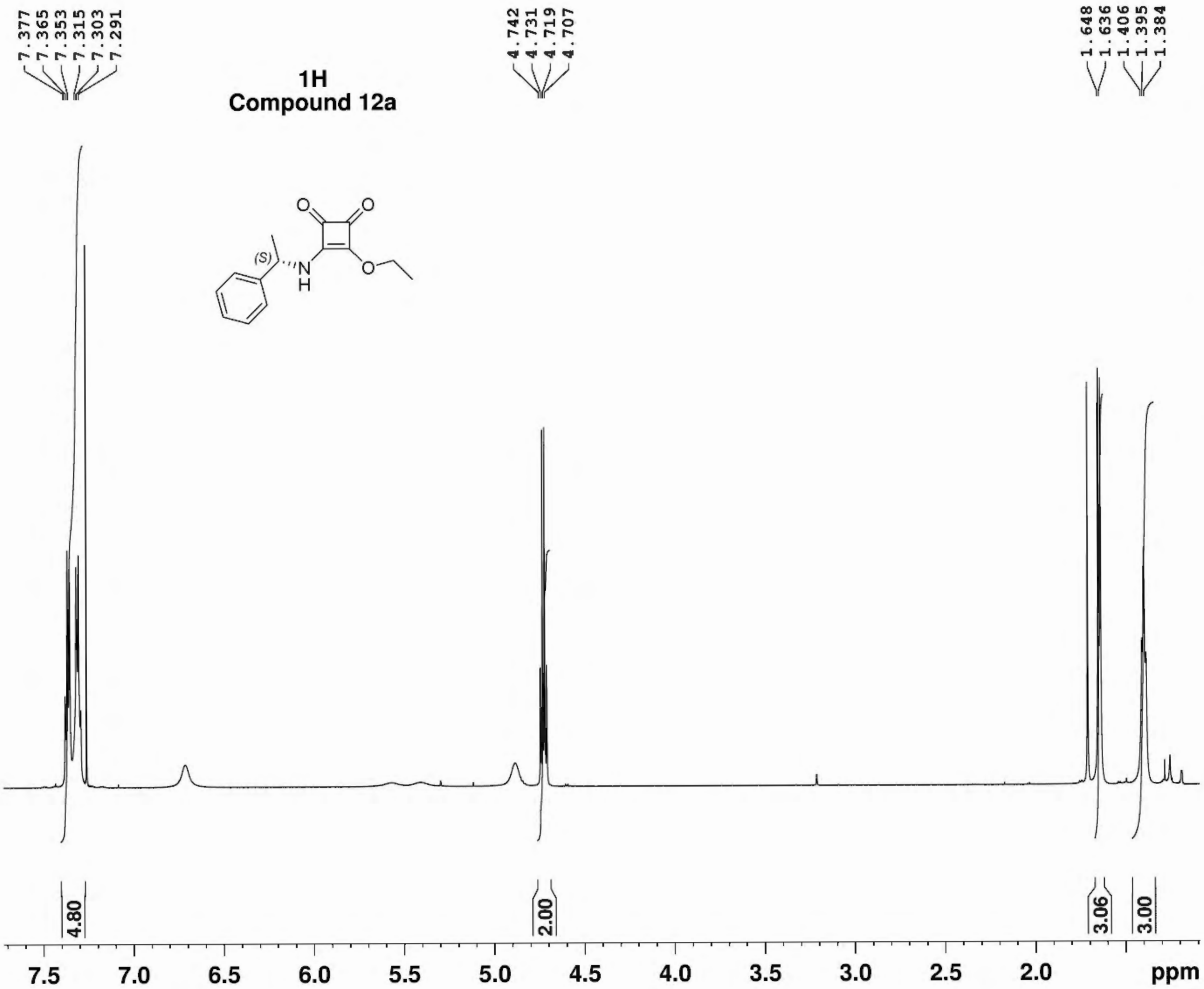

NAME	YND01404A
EXPNO	11
PROCNO	1
Date_	20160912
Time	18.47
INSTRUM	spect
PROBHD	5 mm PABBO BB-
PULPROG	zg30
TD	32768
SOLVENT	CDCl3
NS	32
DS	0
SWH	9615.385 Hz
FIDRES	0.293438 Hz
AQ	1.7039860 sec
RG	256
DW	52.000 usec
DE	13.95 usec
TE	298.0 K
D1	1.00000000 sec
TD0	1

======== CHANNEL f1 ========

SFO1	600.1145608 MHz
NUC1	1H
P1	10.85 usec
SI	65536
SF	600.1100150 MHz
WDW	EM
SSB	0
LB	0.00 Hz
GB	0
PC	1.00

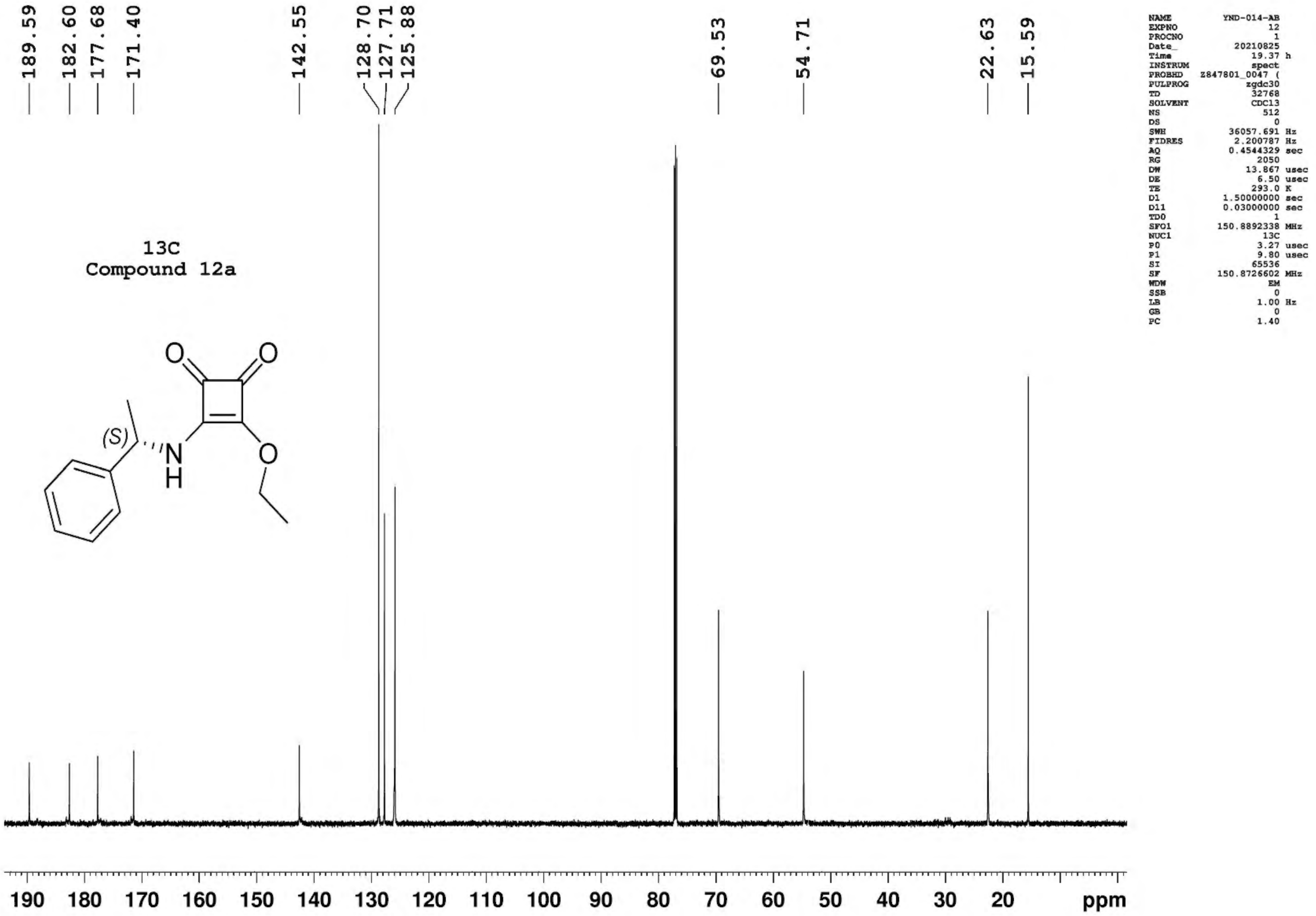
189.59
182.60
177.68
171.40
142.55
128.70
127.71
125.88
69.53
54.71
22.63
15.59
13C
Compound 12a
(S)
O
O
N
H
O
NAME YND-014-AB
EXPNO 12
PROCNO 1
Date_ 20210825
Time 19.37 h
INSTRUM spect
PROBHD Z847801_0047 (
PULPROG zgdc30
TD 32768
SOLVENT CDCl3
NS 512
DS 0
SWH 36057.691 Hz
FIDRES 2.200787 Hz
AQ 0.4544329 sec
RG 2050
DW 13.867 usec
DE 6.50 usec
TE 293.0 K
D1 1.50000000 sec
D11 0.03000000 sec
TD0 1
SFO1 150.8892338 MHz
NUC1 13C
P0 3.27 usec
P1 9.80 usec
SI 65536
SF 150.8726602 MHz
WDW EM
SSB 0
LB 1.00 Hz
GB 0
PC 1.40
190 180 170 160 150 140 130 120 110 100 90 80 70 60 50 40 30 20 ppm

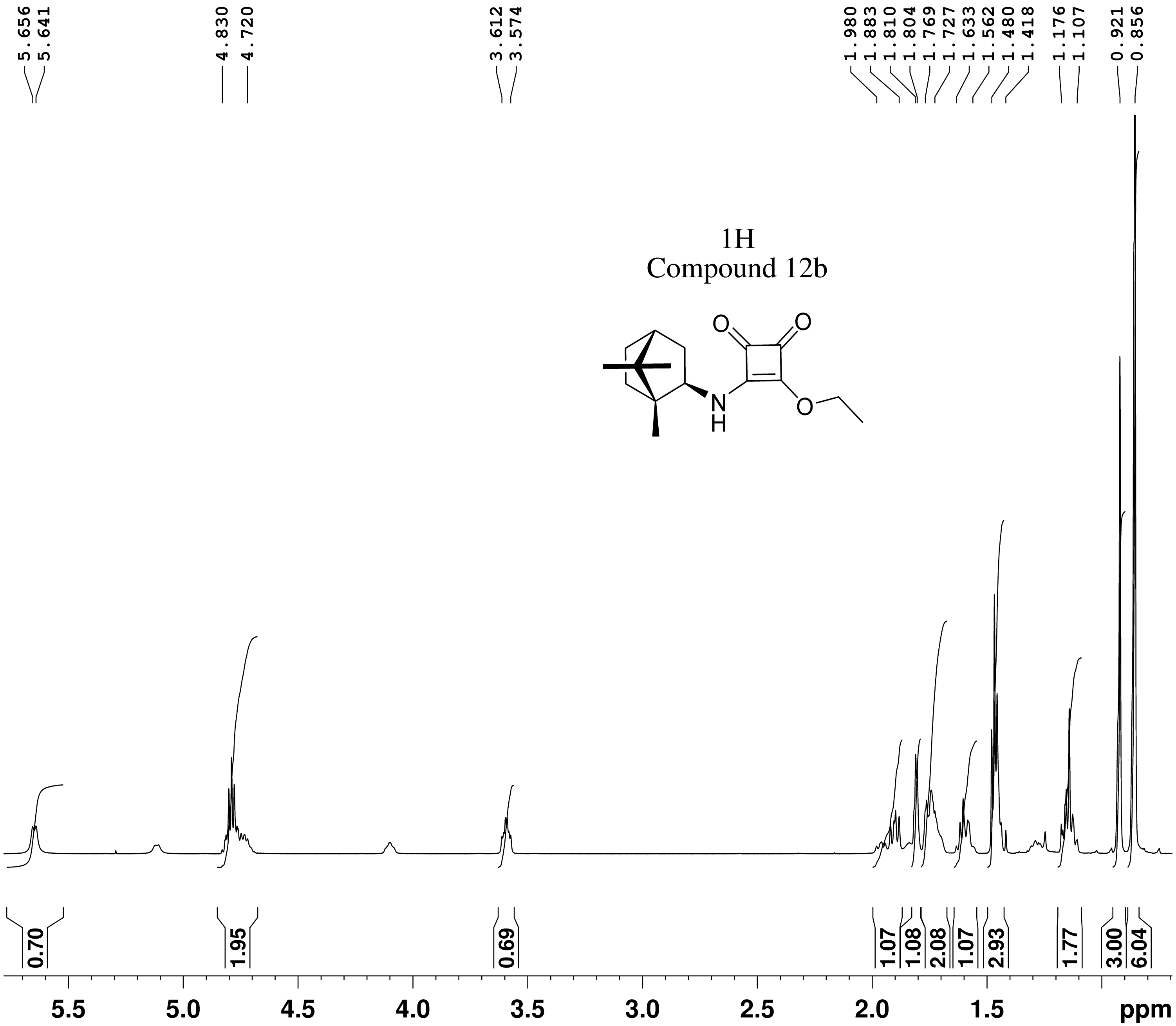

NAME	YNC06308
EXPNO	11
PROCNO	1
Date_	20150505
Time	7.46
INSTRUM	spect
PROBHD	5 mm PABBO BB-
PULPROG	zg30
TD	32768
SOLVENT	CDCl3
NS	32
DS	0
SWH	9615.385 Hz
FIDRES	0.293438 Hz
AQ	1.7039860 sec
RG	161
DW	52.000 usec
DE	13.95 usec
TE	298.0 K
D1	1.00000000 sec
TD0	1

======== CHANNEL f1 ========

SFO1	600.1145608 MHz
NUC1	1H
P1	10.85 usec
SI	65536
SF	600.1100148 MHz
WDW	EM
SSB	0
LB	0.00 Hz
GB	0
PC	1.00

13C
Compound 12b

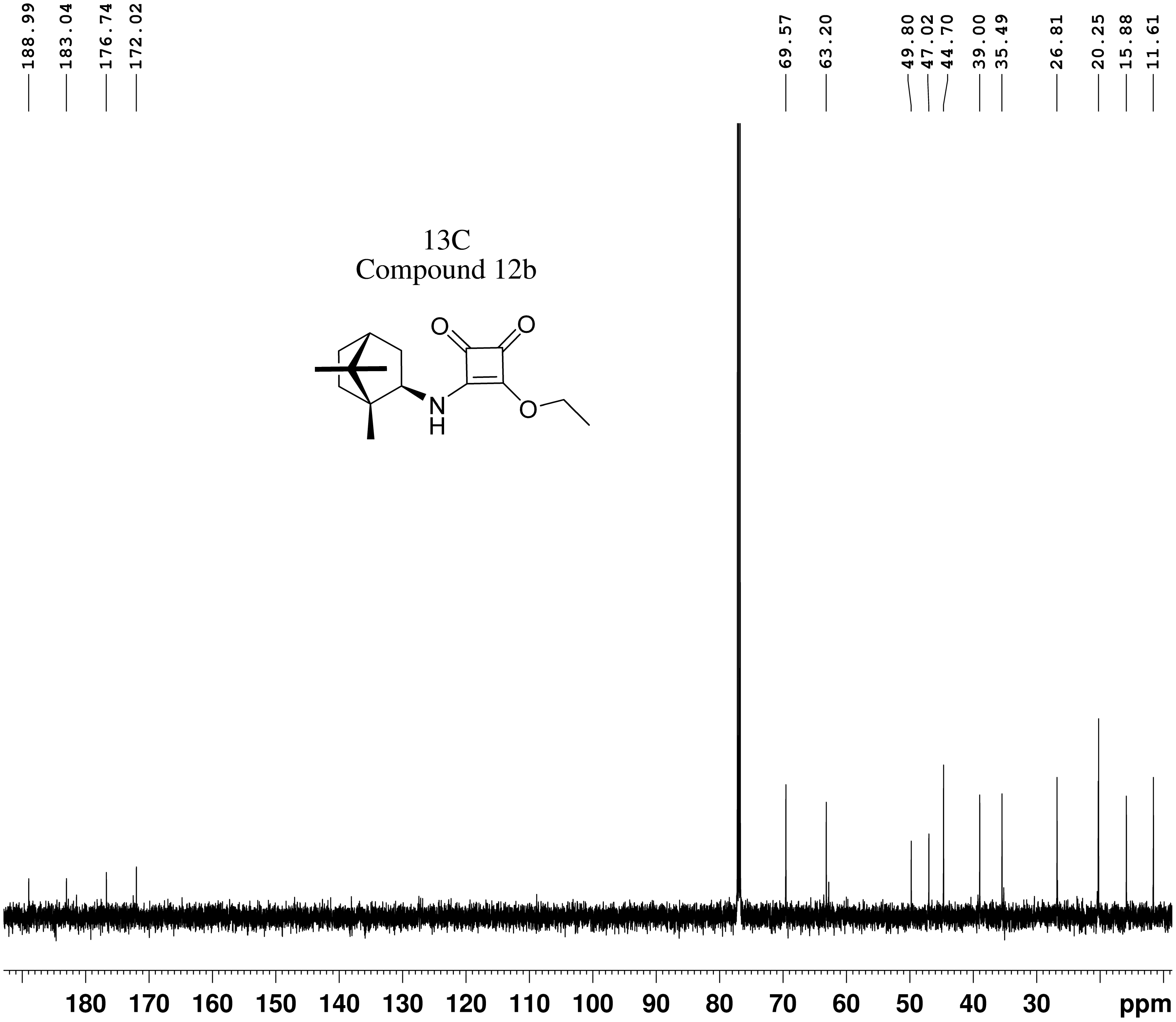

NAME	YNC06308	
EXPNO	12	
PROCNO	1	
Date_	20150505	
Time	7.51	
INSTRUM	spect	
PROBHD	5 mm PABBO BB-	
PULPROG	zgdc30	
TD	32768	
SOLVENT	CDCl3	
NS	128	
DS	0	
SWH	36057.691	Hz
FIDRES	1.100393	Hz
AQ	0.4544329	sec
RG	2050	
DW	13.867	usec
DE	6.50	usec
TE	298.0	K
D1	1.50000000	sec
D11	0.03000000	sec
TD0	1	

======== CHANNEL f1 ========

SFO1	150.9143788	MHz
NUC1	13C	
P1	9.80	usec
SI	65536	
SF	150.8977847	MHz
WDW	EM	
SSB	0	
LB	1.00	Hz
GB	0	
PC	1.40	

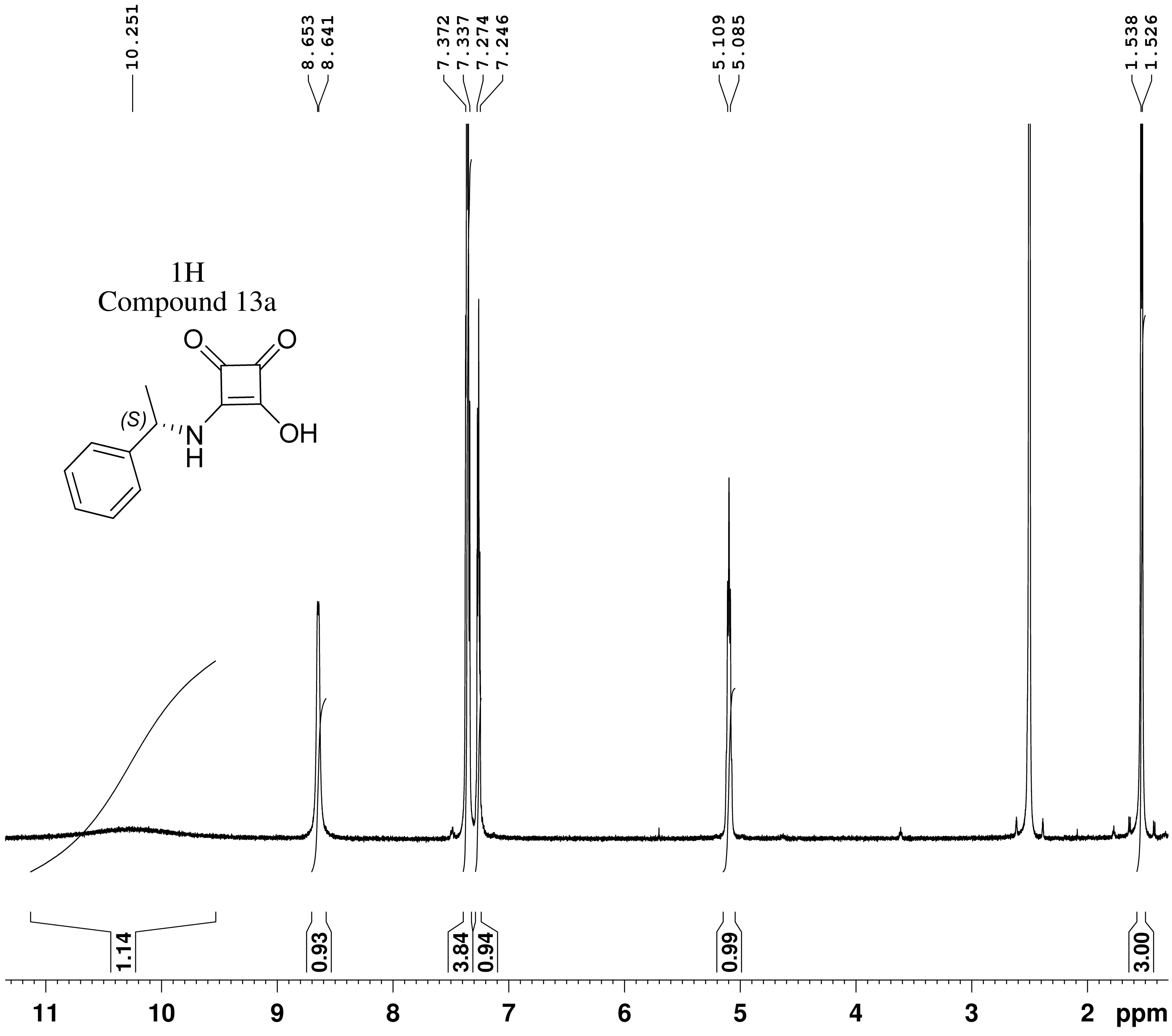

NAME	YNC-07202B	
EXPNO	11	
PROCNO	1	
Date_	20150404	
Time	12.07	
INSTRUM	spect	
PROBHD	5 mm PABBO BB-	
PULPROG	zg30	
TD	32768	
SOLVENT	DMSO	
NS	16	
DS	0	
SWH	9615.385	Hz
FIDRES	0.293438	Hz
AQ	1.7039860	sec
RG	228	
DW	52.000	usec
DE	13.95	usec
TE	338.0	K
D1	1.00000000	sec
TD0	1	

======== CHANNEL f1 ========

SFO1	600.1145608	MHz
NUC1	1H	
P1	10.85	usec
SI	65536	
SF	600.1100037	MHz
WDW	no	
SSB	0	
LB	0.00	Hz
GB	0	
PC	1.00	

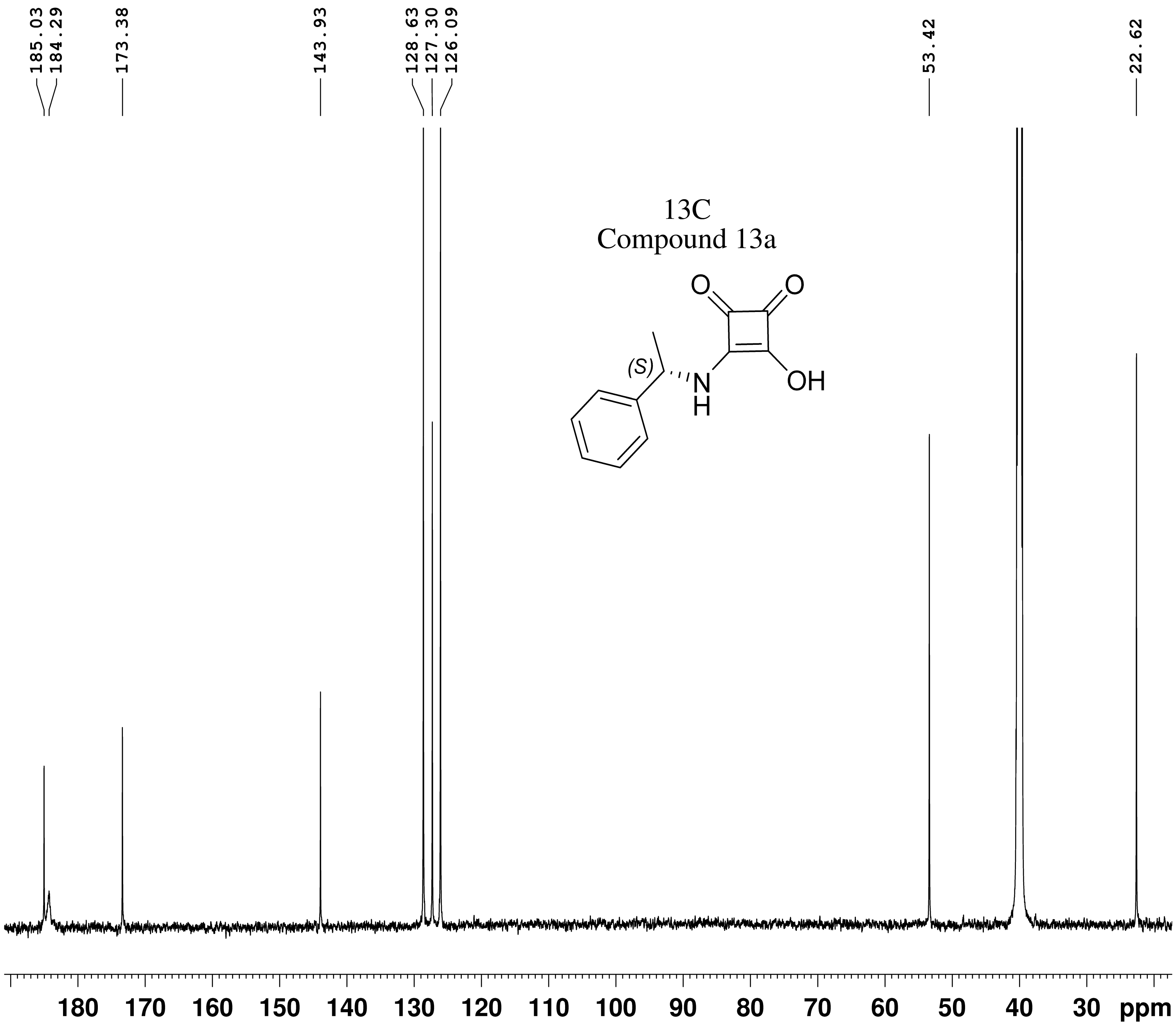

NAME	YNC-07202B
EXPNO	14
PROCNO	1
Date_	20150404
Time	12.59
INSTRUM	spect
PROBHD	5 mm PABBO BB-
PULPROG	zgdc30
TD	32768
SOLVENT	DMSO
NS	8192
DS	0
SWH	36057.691 Hz
FIDRES	1.100393 Hz
AQ	0.4544329 sec
RG	2050
DW	13.867 usec
DE	6.50 usec
TE	338.0 K
D1	1.50000000 sec
D11	0.03000000 sec
TD0	1

======== CHANNEL f1 ========

SFO1	150.9143788 MHz
NUC1	13C
P1	9.80 usec
SI	65536
SF	150.8978255 MHz
WDW	EM
SSB	0
LB	5.00 Hz
GB	0
PC	1.40

1H
Compound 13b

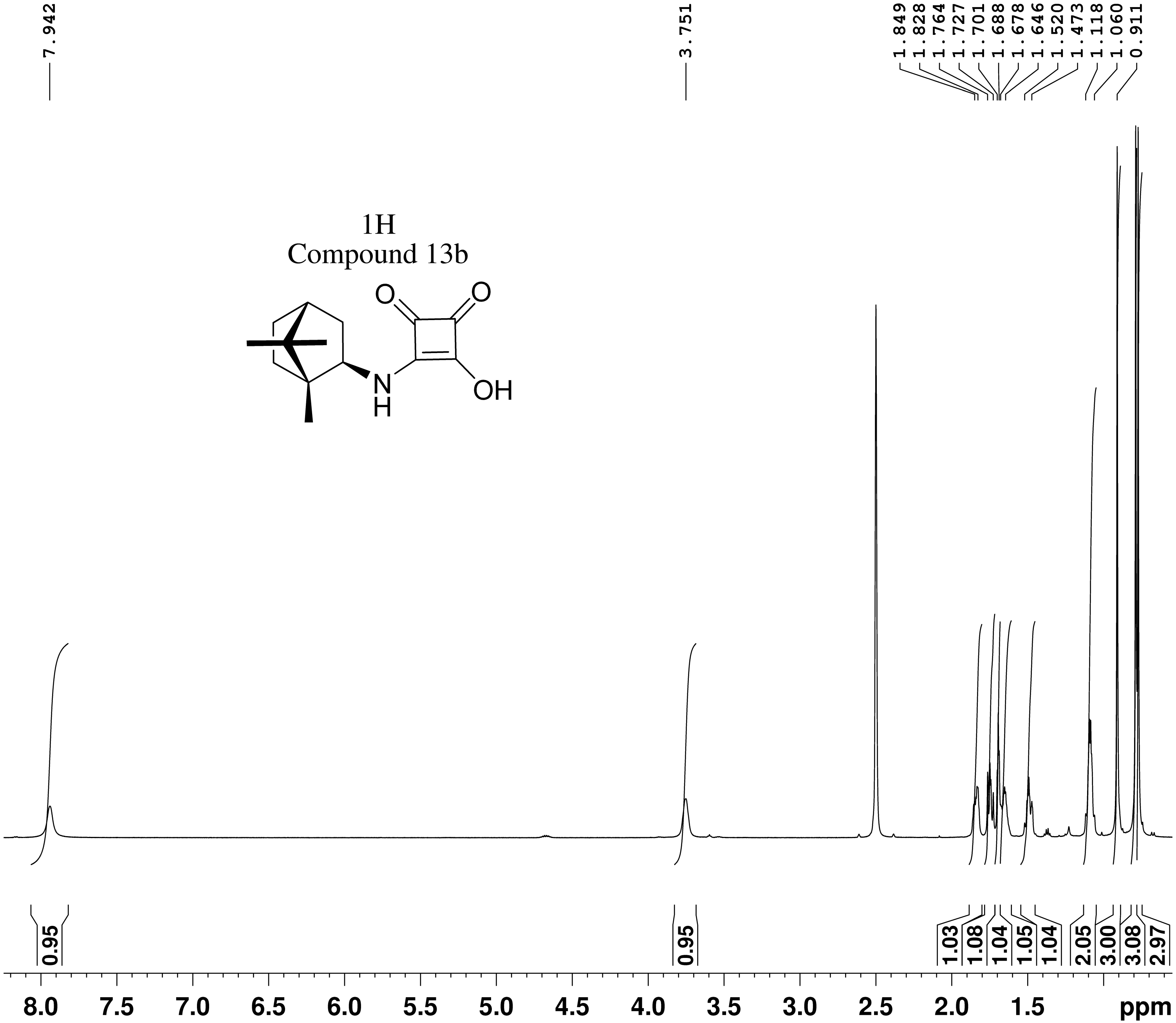

NAME	YNC08602
EXPNO	11
PROCNO	1
Date_	20150722
Time	18.47
INSTRUM	spect
PROBHD	5 mm PABBO BB-
PULPROG	zg30
TD	32768
SOLVENT	CDCl3
NS	32
DS	0
SWH	9615.385 Hz
FIDRES	0.293438 Hz
AQ	1.7039860 sec
RG	203
DW	52.000 usec
DE	13.95 usec
TE	293.0 K
D1	1.00000000 sec
TD0	1

======== CHANNEL f1 ========

SFO1	600.1145608 MHz
NUC1	1H
P1	10.85 usec
SI	65536
SF	600.1128558 MHz
WDW	EM
SSB	0
LB	0.00 Hz
GB	0
PC	1.00

13C
Compound 13b

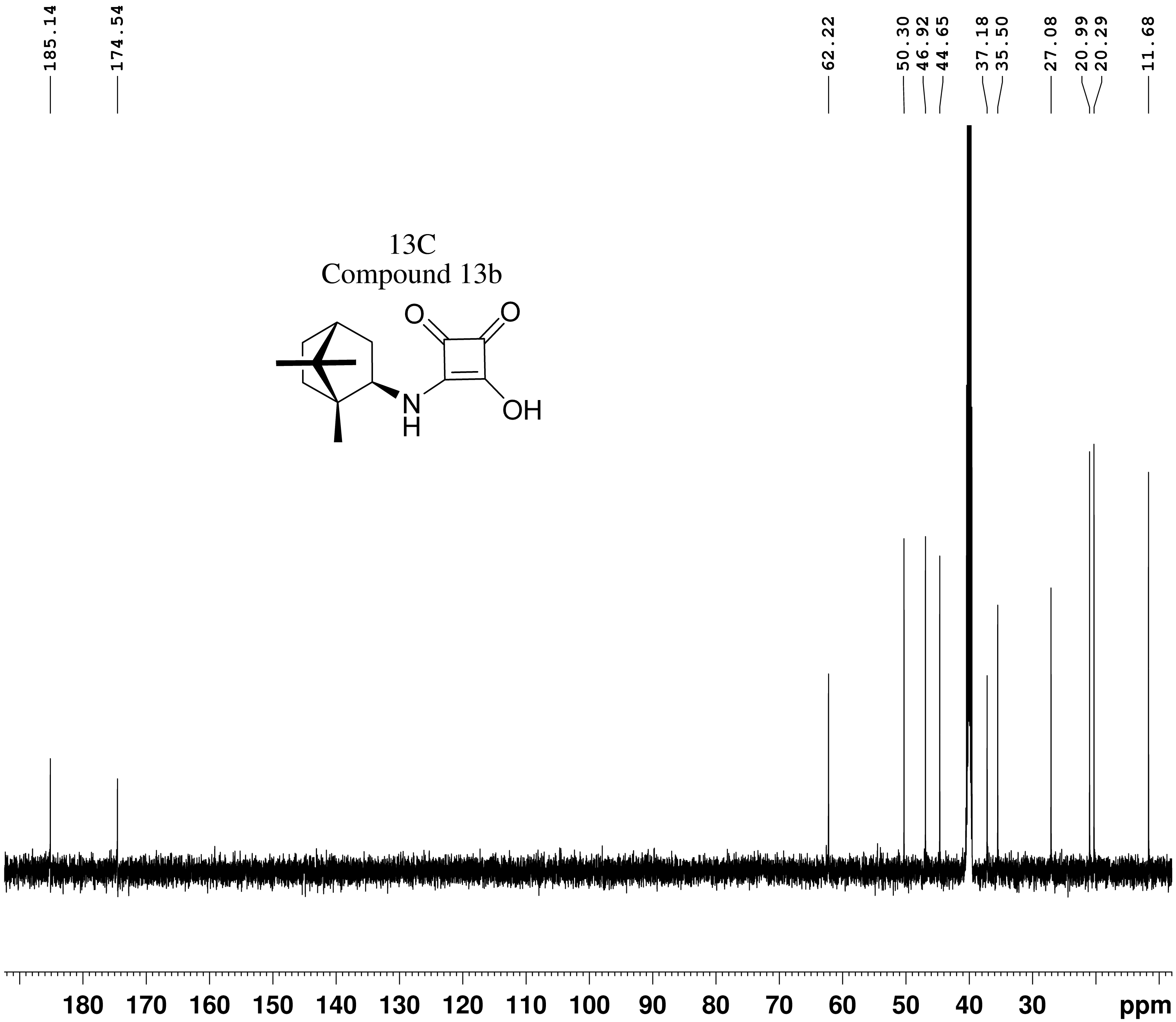

NAME	YNC08602B
EXPNO	12
PROCNO	1
Date_	20150725
Time	0.11
INSTRUM	spect
PROBHD	5 mm PABBO BB-
PULPROG	zgdc30
TD	32768
SOLVENT	DMSO
NS	128
DS	0
SWH	36057.691 Hz
FIDRES	1.100393 Hz
AQ	0.4544329 sec
RG	2050
DW	13.867 usec
DE	6.50 usec
TE	293.0 K
D1	1.50000000 sec
D11	0.03000000 sec
TD0	1

======== CHANNEL f1 ========

SFO1	150.9143788 MHz
NUC1	13C
P1	9.80 usec
SI	65536
SF	150.8977722 MHz
WDW	EM
SSB	0
LB	1.00 Hz
GB	0
PC	1.40

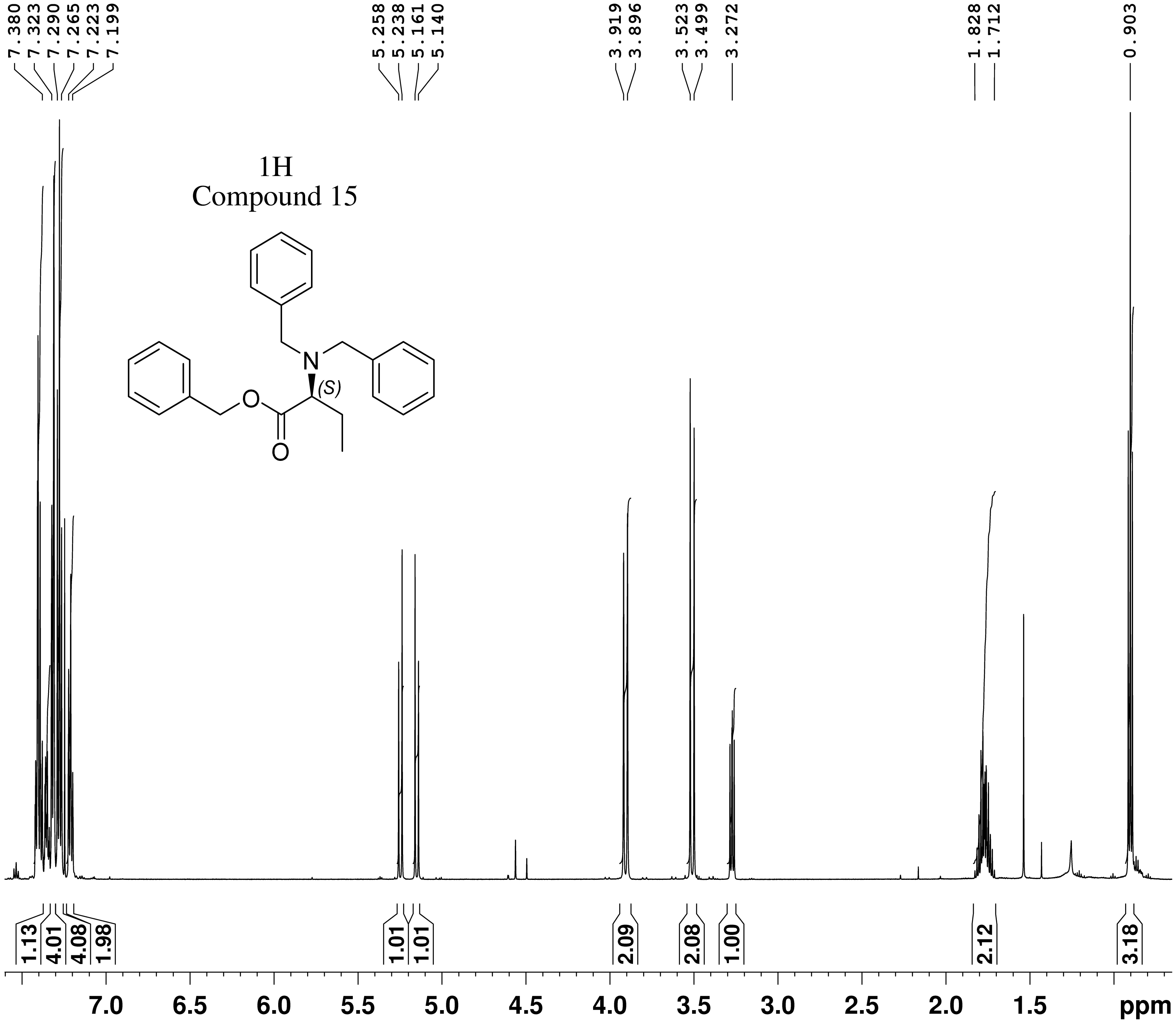

NAME	YND01703A	
EXPNO	11	
PROCNO	1	
Date_	20160916	
Time	14.18	
INSTRUM	spect	
PROBHD	5 mm PABBO BB-	
PULPROG	zg30	
TD	32768	
SOLVENT	CDCl3	
NS	16	
DS	0	
SWH	9615.385	Hz
FIDRES	0.293438	Hz
AQ	1.7039860	sec
RG	161	
DW	52.000	usec
DE	13.95	usec
TE	298.0	K
D1	1.00000000	sec
TD0	1	

======== CHANNEL f1 ========

SFO1	600.1145608	MHz
NUC1	1H	
P1	10.85	usec
SI	65536	
SF	600.1100224	MHz
WDW	EM	
SSB	0	
LB	0.00	Hz
GB	0	
PC	1.00	

13C
Compound 15

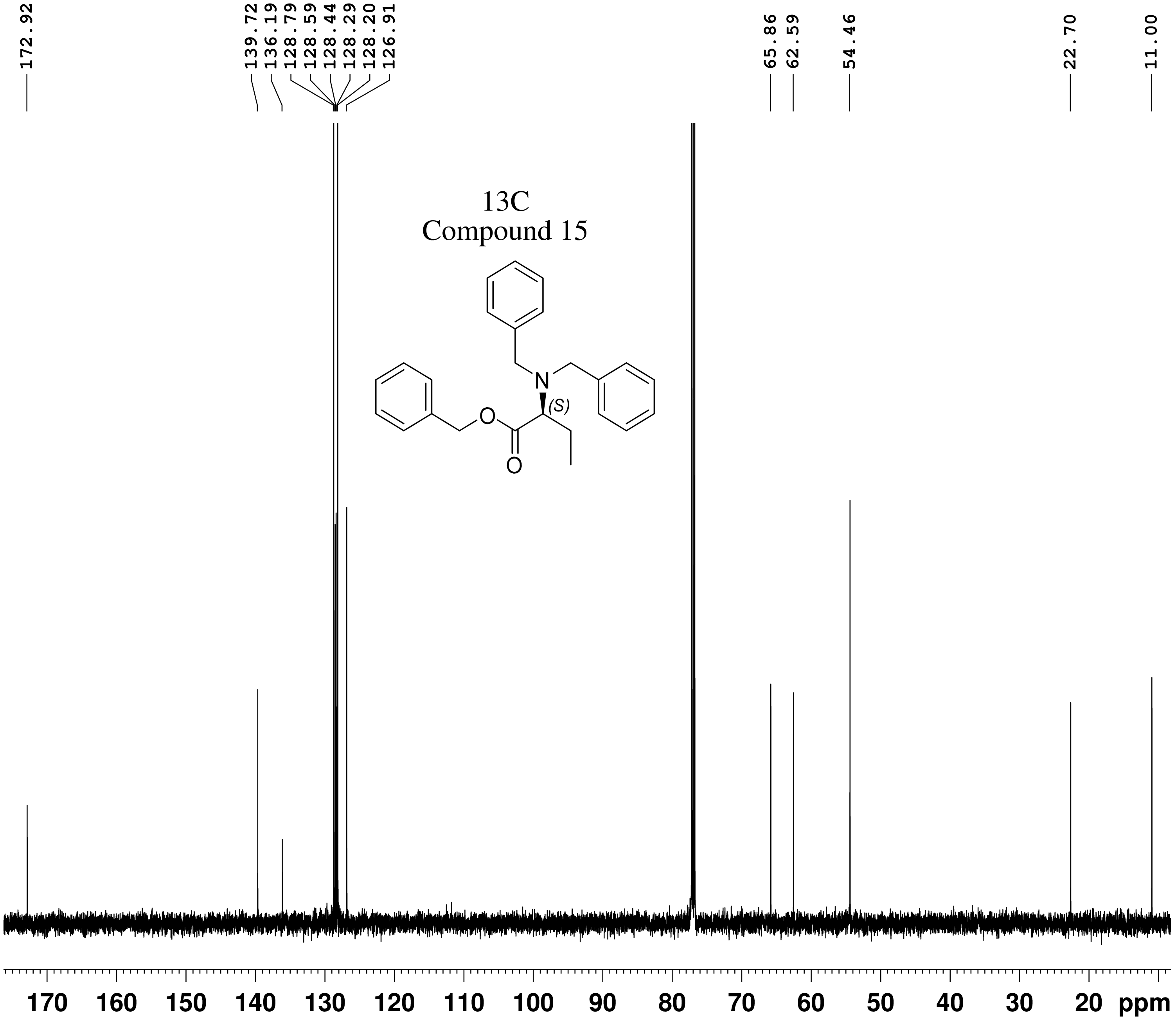

NAME	YND01703A
EXPNO	12
PROCNO	1
Date_	20160916
Time	14.22
INSTRUM	spect
PROBHD	5 mm PABBO BB-
PULPROG	zgdc30
TD	32768
SOLVENT	CDCl3
NS	128
DS	0
SWH	36057.691 Hz
FIDRES	1.100393 Hz
AQ	0.4544329 sec
RG	2050
DW	13.867 usec
DE	6.50 usec
TE	298.0 K
D1	1.50000000 sec
D11	0.03000000 sec
TD0	1

======== CHANNEL f1 ========

SFO1	150.9143788 MHz
NUC1	13C
P1	9.80 usec
SI	65536
SF	150.8977850 MHz
WDW	EM
SSB	0
LB	1.00 Hz
GB	0
PC	1.40

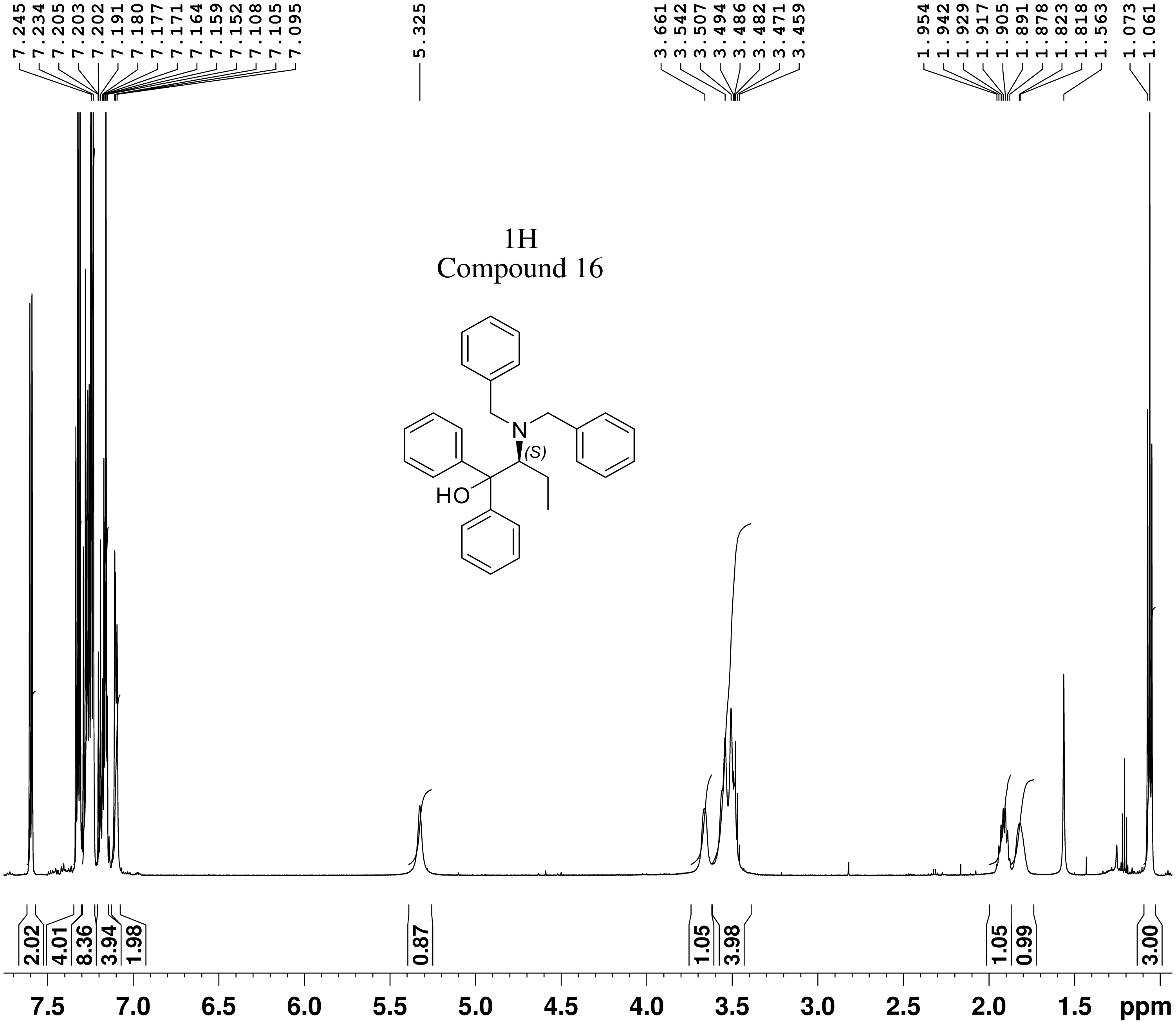

NAME	YNC09203A
EXPNO	11
PROCNO	1
Date_	20150901
Time	19.34
INSTRUM	spect
PROBHD	5 mm PABBO BB-
PULPROG	zg30
TD	32768
SOLVENT	CDCl3
NS	16
DS	0
SWH	9615.385 Hz
FIDRES	0.293438 Hz
AQ	1.7039860 sec
RG	128
DW	52.000 usec
DE	13.95 usec
TE	293.0 K
D1	1.00000000 sec
TD0	1

======== CHANNEL f1 ========

SFO1	600.1145608 MHz
NUC1	1H
P1	10.85 usec
SI	65536
SF	600.1100236 MHz
WDW	EM
SSB	0
LB	0.00 Hz
GB	0
PC	1.00

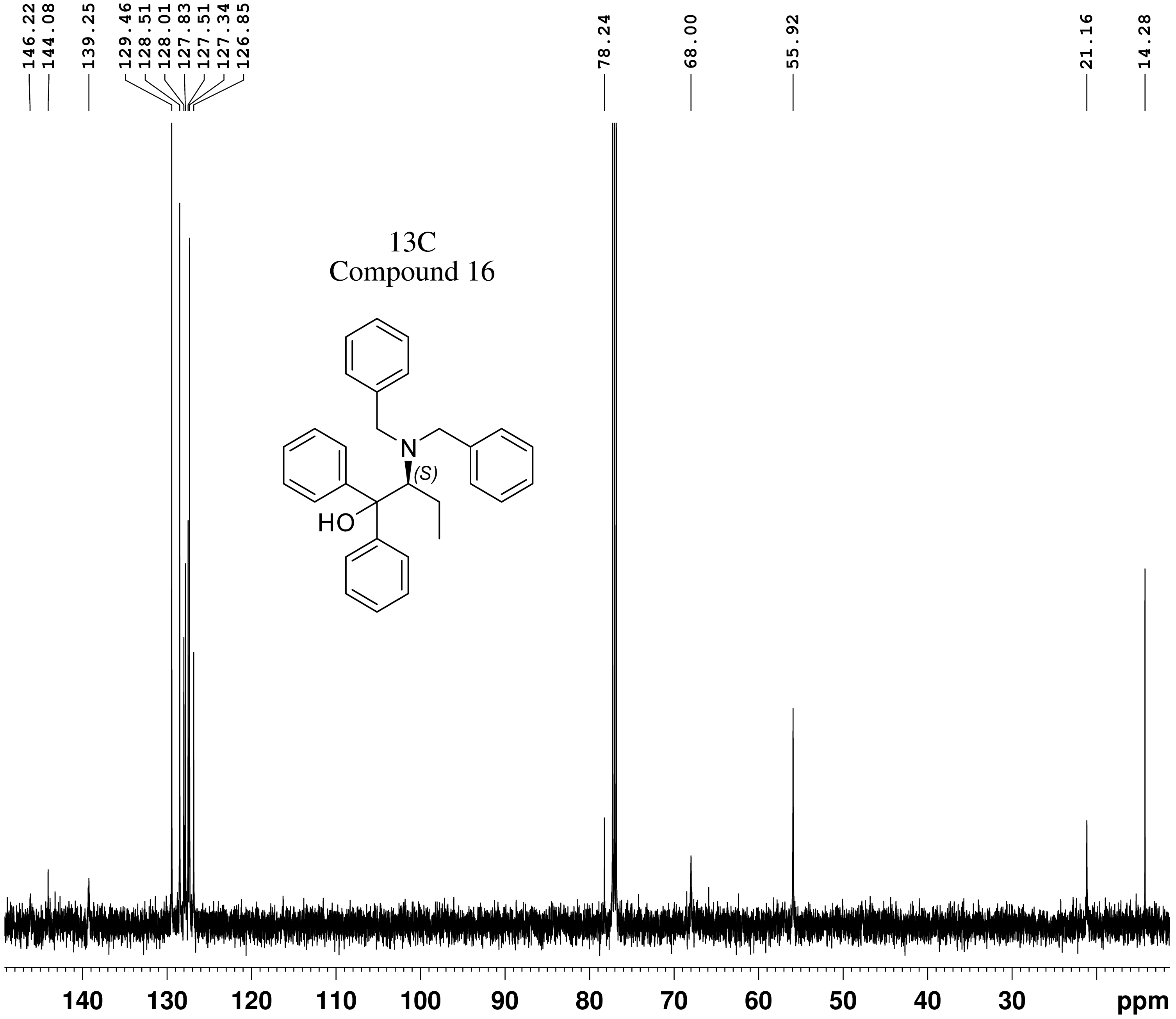

NAME	YNC09203A
EXPNO	12
PROCNO	1
Date_	20150901
Time	19.38
INSTRUM	spect
PROBHD	5 mm PABBO BB-
PULPROG	zgdc30
TD	32768
SOLVENT	CDCl3
NS	128
DS	0
SWH	36057.691 Hz
FIDRES	1.100393 Hz
AQ	0.4544329 sec
RG	2050
DW	13.867 usec
DE	6.50 usec
TE	293.0 K
D1	1.50000000 sec
D11	0.03000000 sec
TD0	1

======== CHANNEL f1 ========

SFO1	150.9143788 MHz
NUC1	13C
P1	9.80 usec
SI	65536
SF	150.8977800 MHz
WDW	EM
SSB	0
LB	1.00 Hz
GB	0
PC	1.40

1H
Compound 17

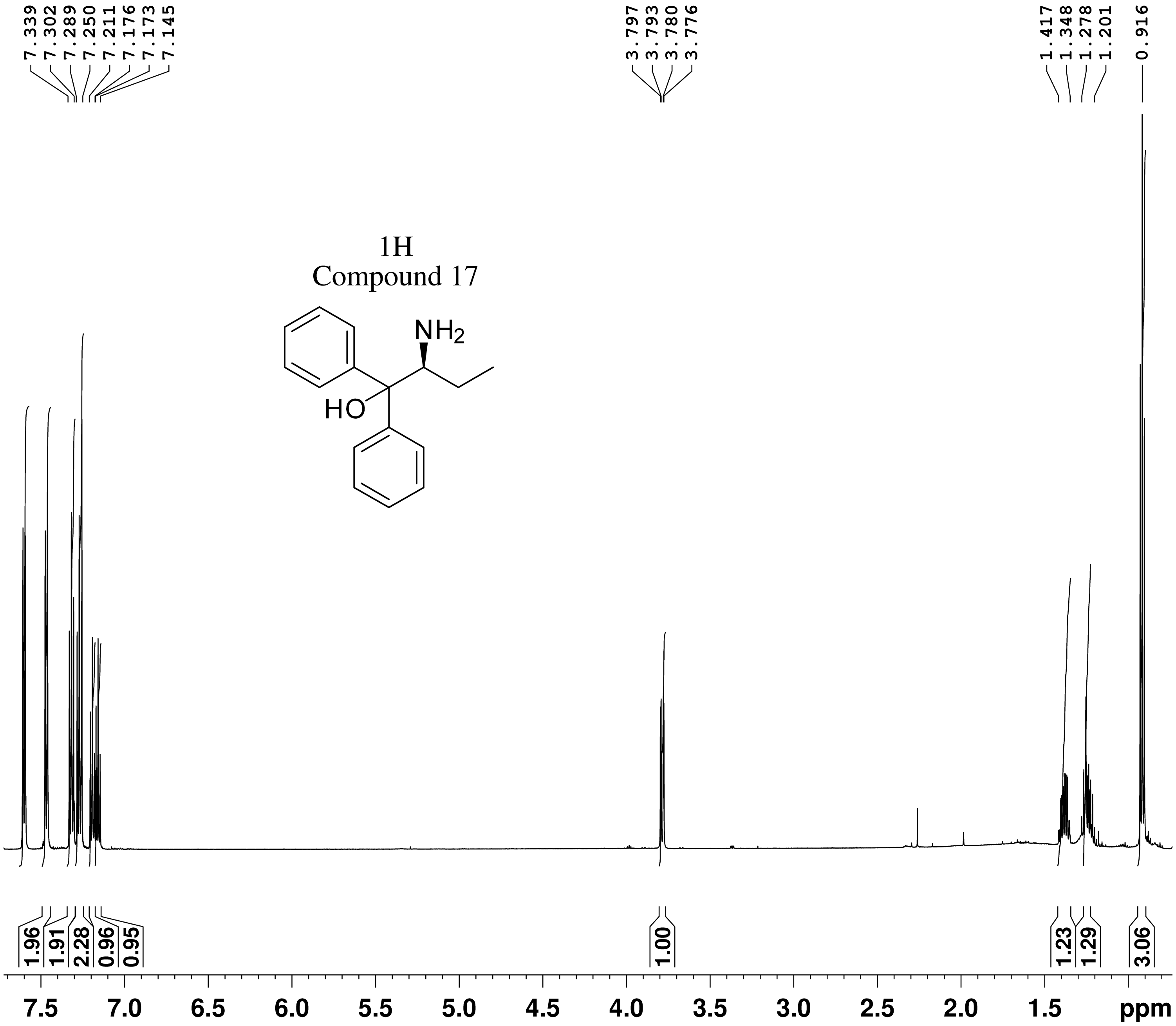

NAME	YNC09604A	
EXPNO	11	
PROCNO	1	
Date_	20150930	
Time	21.23	h
INSTRUM	spect	
PROBHD	Z847801_0047 (	
PULPROG	zg30	
TD	32768	
SOLVENT	CDCl3	
NS	32	
DS	0	
SWH	9615.385	Hz
FIDRES	0.293438	Hz
AQ	1.7039860	sec
RG	181	
DW	52.000	usec
DE	13.95	usec
TE	293.0	K
D1	1.00000000	sec
TD0	1	
SFO1	600.1145608	MHz
NUC1	1H	
P1	10.85	usec
SI	65536	
SF	600.1100185	MHz
WDW	EM	
SSB	0	
LB	0.00	Hz
GB	0	
PC	1.00	

13C
Compound 17

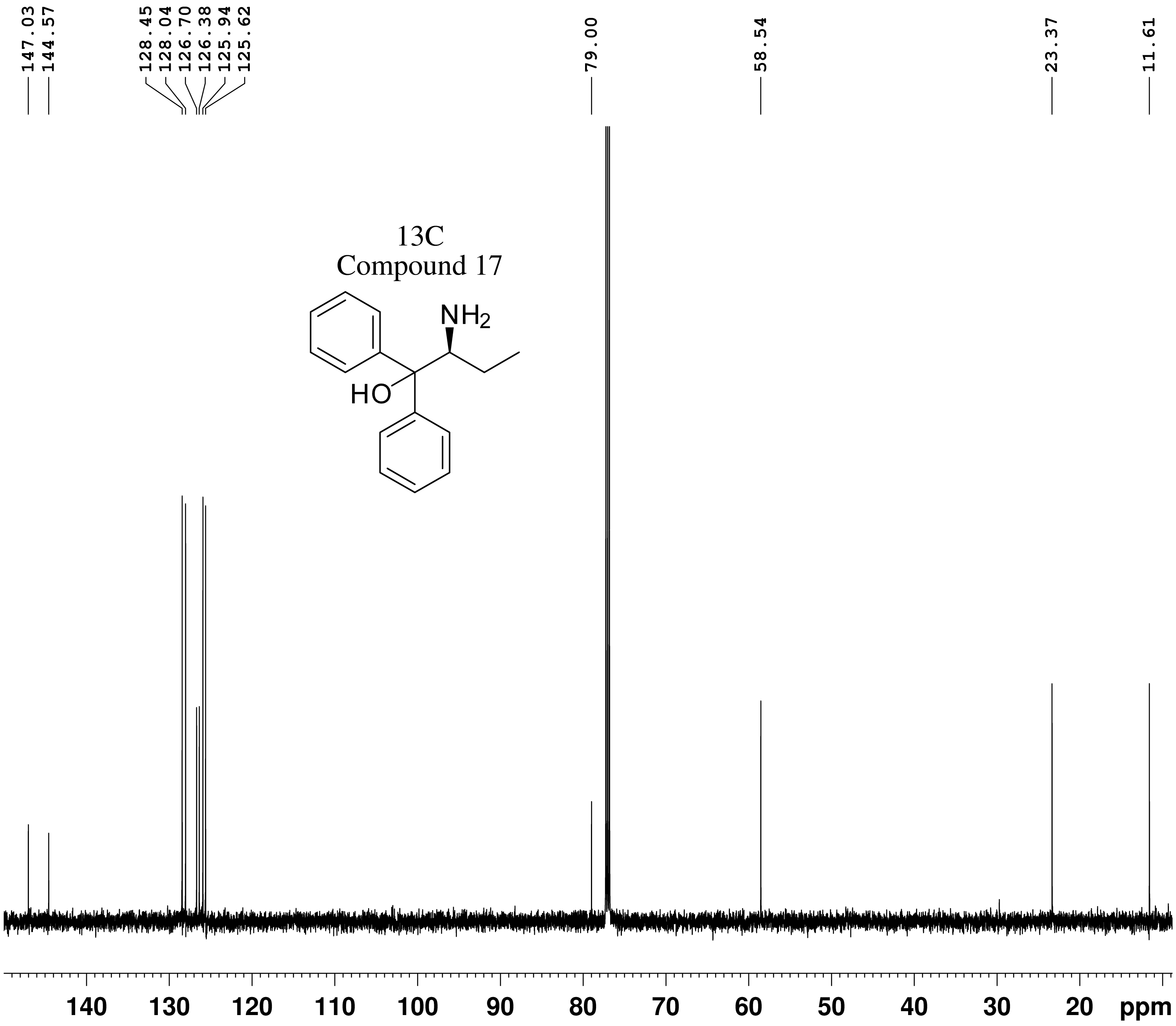

NAME	YNC09604A	
EXPNO	12	
PROCNO	1	
Date_	20150930	
Time	21.28	h
INSTRUM	spect	
PROBHD	Z847801_0047 (	
PULPROG	zgdc30	
TD	32768	
SOLVENT	CDCl3	
NS	128	
DS	0	
SWH	36057.691	Hz
FIDRES	1.100393	Hz
AQ	0.4544329	sec
RG	2050	
DW	13.867	usec
DE	6.50	usec
TE	293.0	K
D1	1.50000000	sec
D11	0.03000000	sec
TD0	1	
SFO1	150.9143788	MHz
NUC1	13C	
P1	9.80	usec
SI	65536	
SF	150.8977800	MHz
WDW	EM	
SSB	0	
LB	1.00	Hz
GB	0	
PC	1.40	

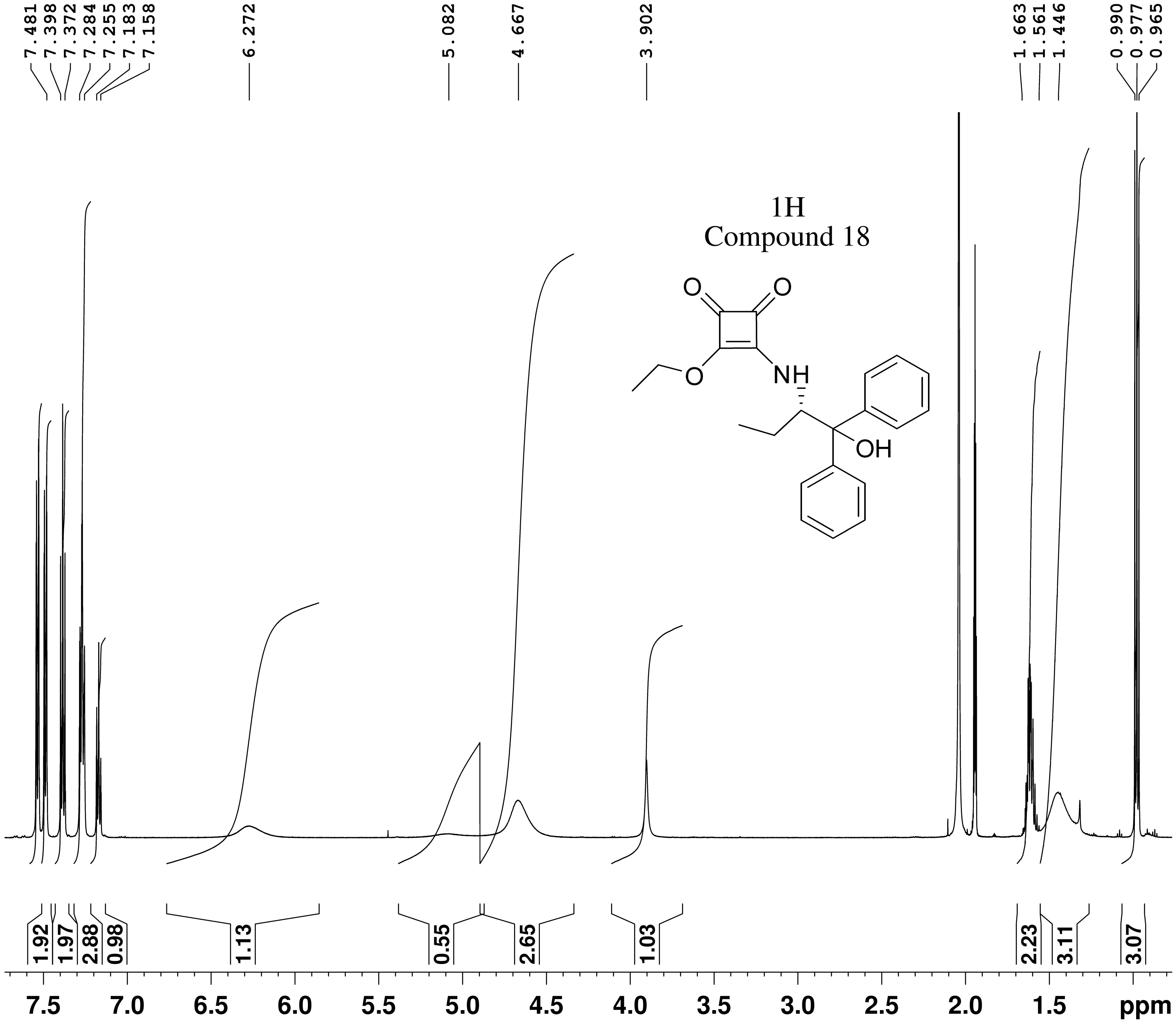

NAME	YNC10405C	
EXPNO	71	
PROCNO	1	
Date_	20160209	
Time	15.23	
INSTRUM	spect	
PROBHD	5 mm PABBO BB-	
PULPROG	zg30	
TD	32768	
SOLVENT	CD3CN	
NS	16	
DS	0	
SWH	9615.385	Hz
FIDRES	0.293438	Hz
AQ	1.7039860	sec
RG	161	
DW	52.000	usec
DE	13.95	usec
TE	348.1	K
D1	1.00000000	sec
TD0	1	
========	CHANNEL f1 ========	
SFO1	600.1145608	MHz
NUC1	1H	
P1	10.85	usec
SI	65536	
SF	600.1100139	MHz
WDW	no	
SSB	0	
LB	0.00	Hz
GB	0	
PC	1.00	

13C
Compound 18

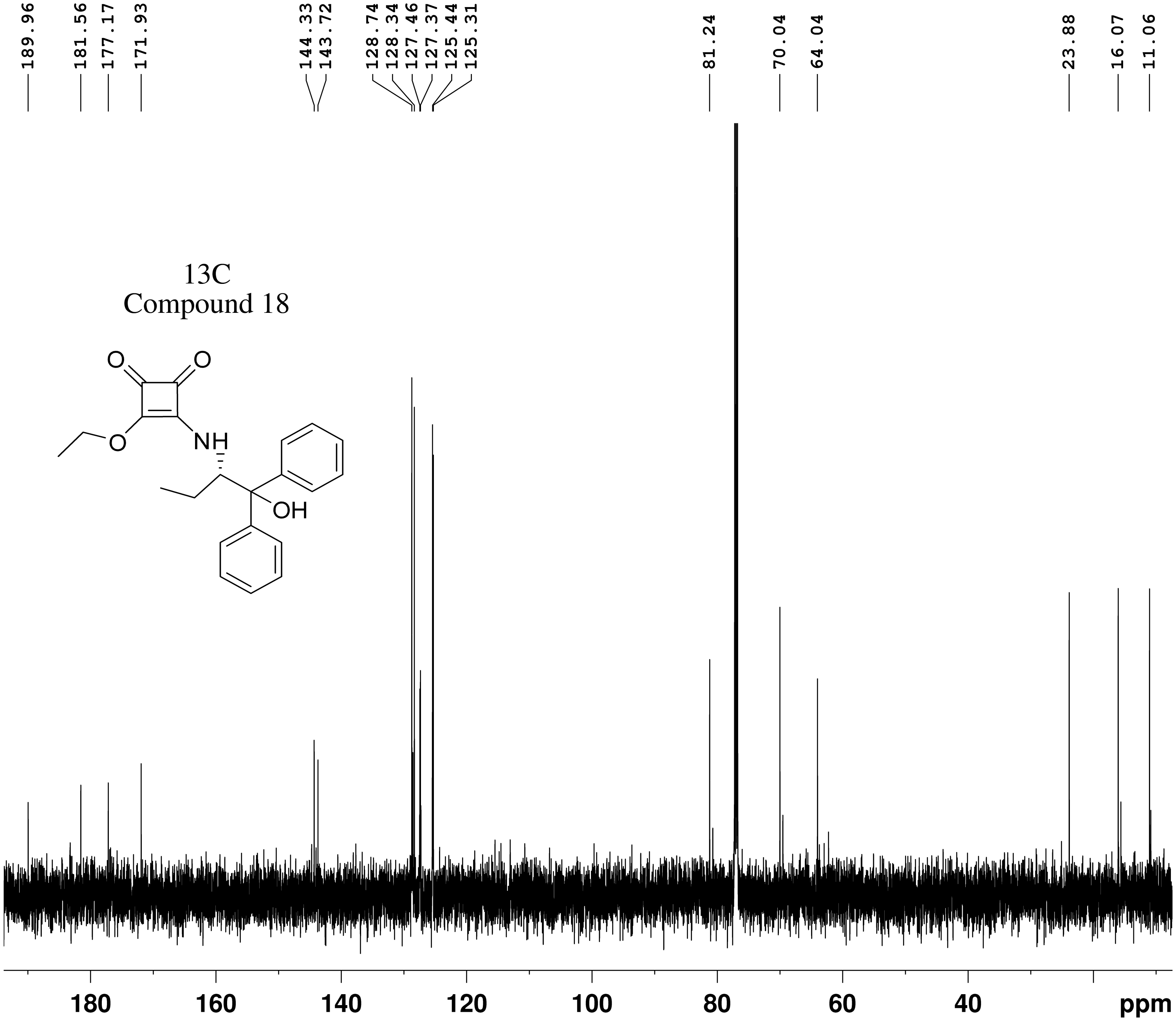

NAME	YNC10405A
EXPNO	12
PROCNO	1
Date_	20151217
Time	14.58 h
INSTRUM	spect
PROBHD	Z847801_0047 (
PULPROG	zgdc30
TD	32768
SOLVENT	CDCl3
NS	128
DS	0
SWH	36057.691 Hz
FIDRES	1.100393 Hz
AQ	0.4544329 sec
RG	2050
DW	13.867 usec
DE	6.50 usec
TE	293.1 K
D1	1.50000000 sec
D11	0.03000000 sec
TD0	1
SFO1	150.9143788 MHz
NUC1	13C
P1	9.80 usec
SI	65536
SF	150.8977837 MHz
WDW	EM
SSB	0
LB	1.00 Hz
GB	0
PC	1.40

GC: Table 2, Entry 1

SHIMADZU LabSolutions

Analysis Report

<Sample Information>

Sample Name : phprop
Sample ID : phprop
Filename : YNC05002_01.dat.gcd
Method Filename : Yana1a.met
Batch Filename :
Vial # : 3
Injection Volume : 1 uL   Level : 1
Date Acquired : 12/10/2014 5:09:59 PM
Date Processed : 11/1/2021 1:52:33 PM
Sample Type : ME

<Chromatogram>

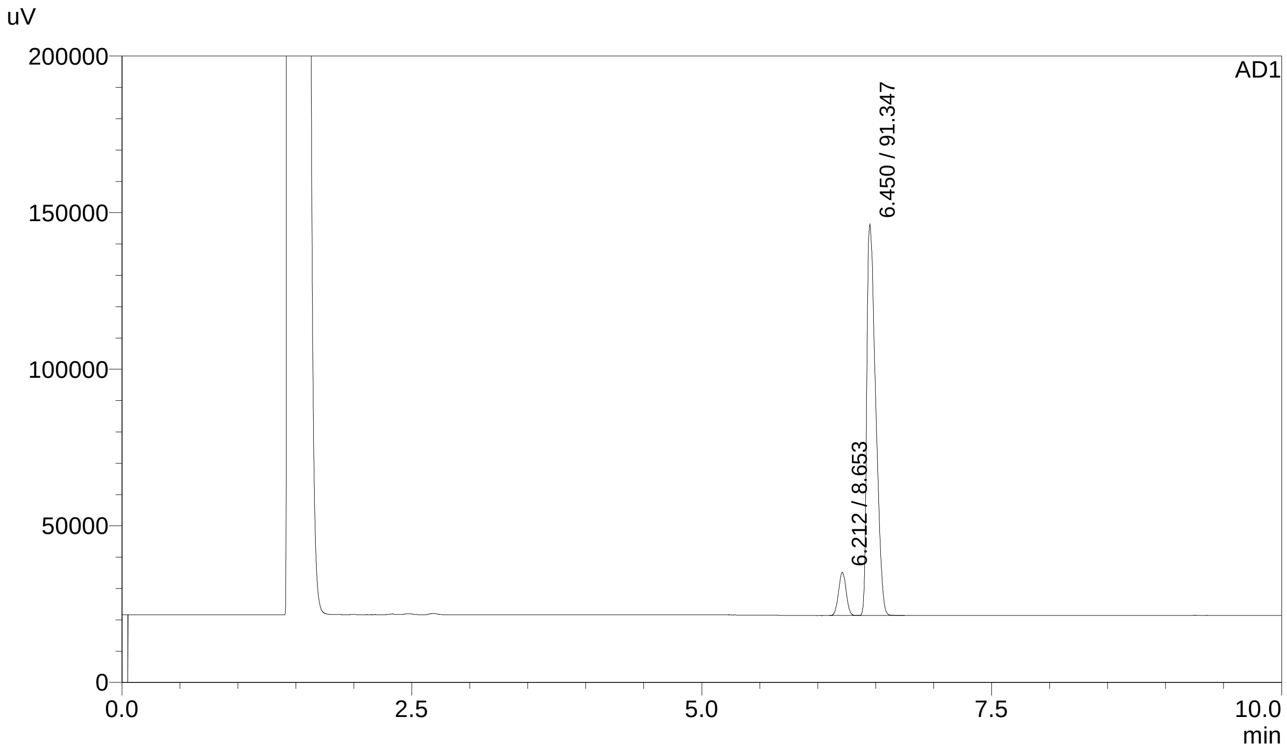

<Peak Table>

AD1

Peak#	Name	Area%	Ret. Time	Area	Height
1		8.653	6.212	63354	13813
2		91.347	6.450	668762	124379
Total		100.000		732116	138192

GC: Table 2, Entry 2

SHIMADZU LabSolutions

Analysis Report

<Sample Information>

Sample Name : clphpr
Sample ID : clphpr
Filename : YNC11202_03.dat
Method Filename : Yana1a.met
Batch Filename :
Vial # : 3
Injection Volume : 1 uL Level : 1
Date Acquired : 2/18/2016 12:18:30 PM
Date Processed : 11/1/2021 2:55:38 PM
Sample Type : ME

<Chromatogram>

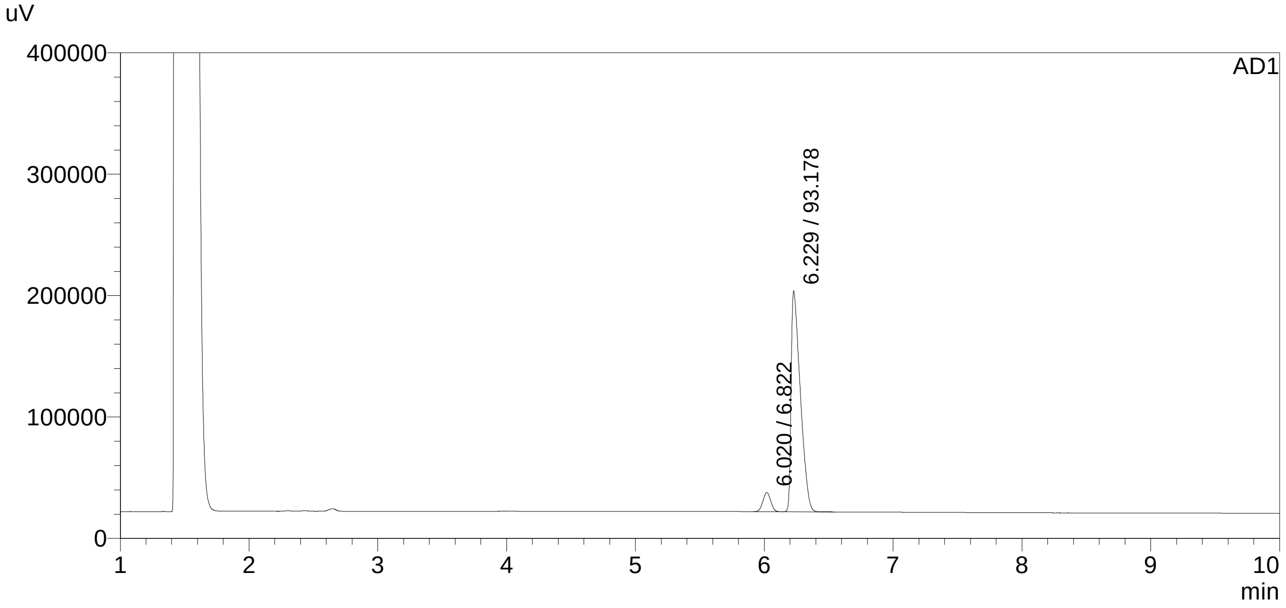

<Peak Table>

AD1

Peak#	Name	Area%	Ret. Time	Area	Height
1		6.822	6.020	66449	15827
2		93.178	6.229	907649	181887
Total		100.000		974098	197714

GC: Table 2, Entry 3

SHIMADZU LabSolutions

Analysis Report

<Sample Information>

Sample Name : clphpr
Sample ID : clphpr
Filename : YNC06603_01.dat
Method Filename : Yana1a.met
Batch Filename :
Vial # : 2
Injection Volume : 1 uL   Level : 1
Date Acquired : 2/12/2015 11:32:41 AM
Date Processed : 11/1/2021 3:16:33 PM
Sample Type : ME

<Chromatogram>

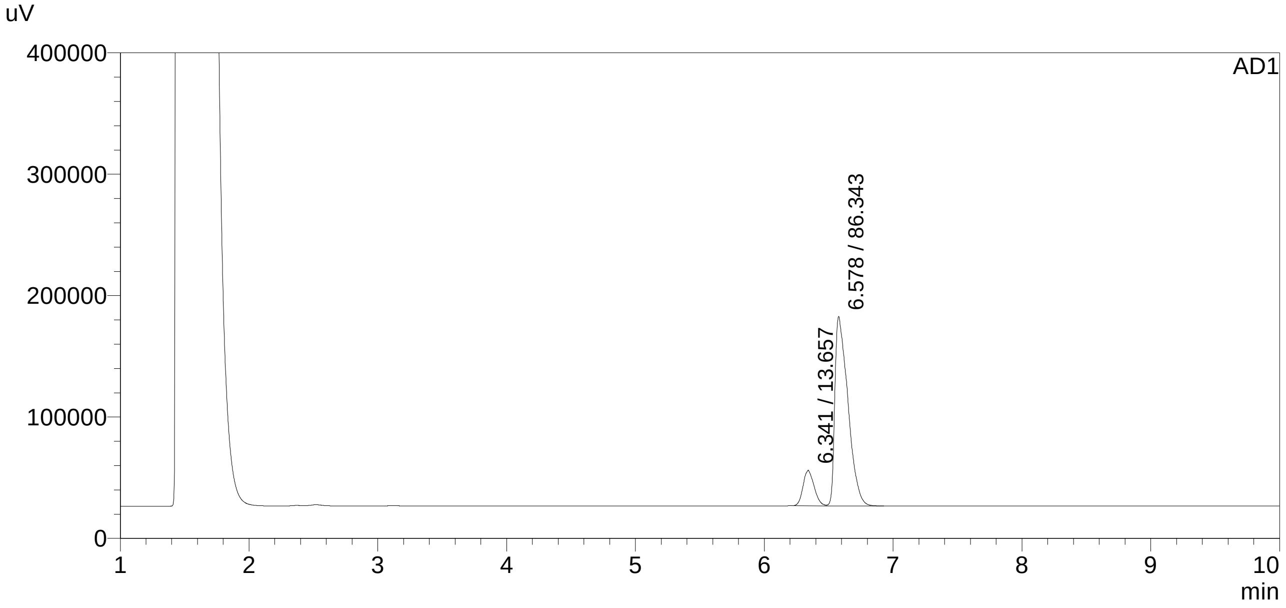

<Peak Table>

AD1

Peak#	Name	Area%	Ret. Time	Area	Height
1		13.657	6.341	174014	29156
2		86.343	6.578	1100147	156002
Total		100.000		1274161	185158

GC: Table 2, Entry 4

SHIMADZU LabSolutions

Analysis Report

<Sample Information>

Sample Name : yana
Sample ID : yana
Filename : YNC06703_03.dat
Method Filename : Yana1a.met
Batch Filename :
Vial # : 3
Injection Volume : 1 uL Level : 1
Date Acquired : 2/12/2015 12:33:51 PM
Date Processed : 11/1/2021 3:22:39 PM
Sample Type : ME

<Chromatogram>

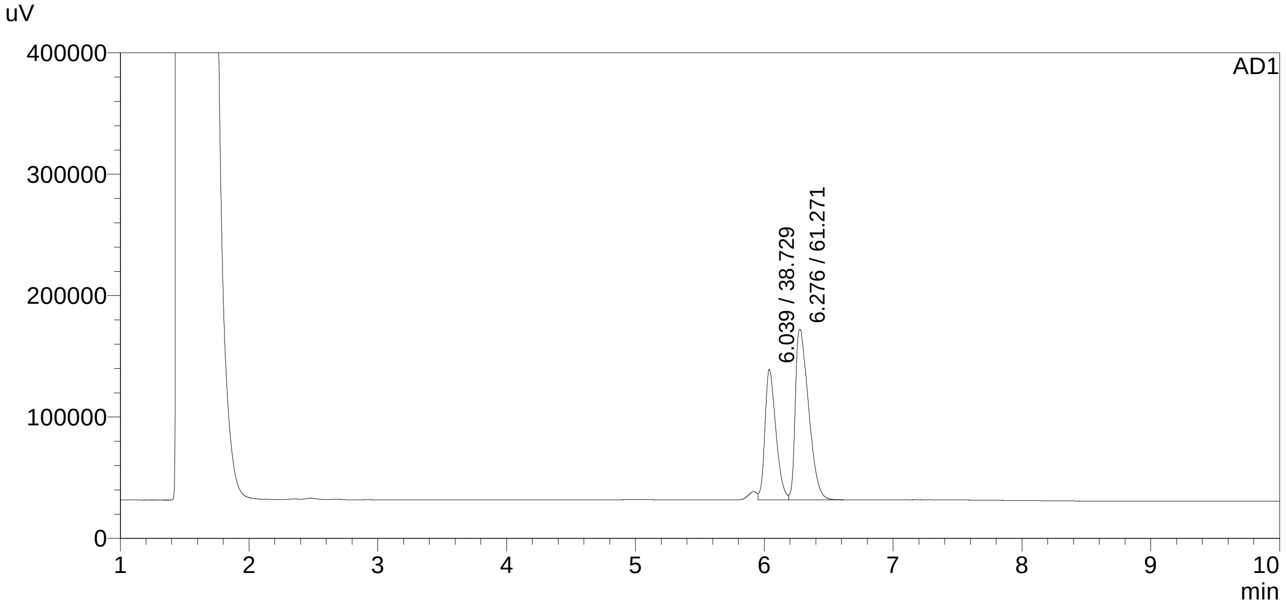

<Peak Table>

AD1

Peak#	Name	Area%	Ret. Time	Area	Height
1		38.729	6.039	635549	107584
2		61.271	6.276	1005468	140261
Total		100.000		1641017	247845

GC: Table 2, Entry 5

SHIMADZU LabSolutions

Analysis Report

<Sample Information>

Sample Name : clphpr
Sample ID : clphpr
Filename : YNC10703_01.dat
Method Filename : Yana1a.met
Batch Filename :
Vial # : 3
Injection Volume : 1 uL Level : 1
Date Acquired : 1/15/2016 1:43:49 PM
Date Processed : 11/1/2021 3:29:53 PM
Sample Type : ME

<Chromatogram>

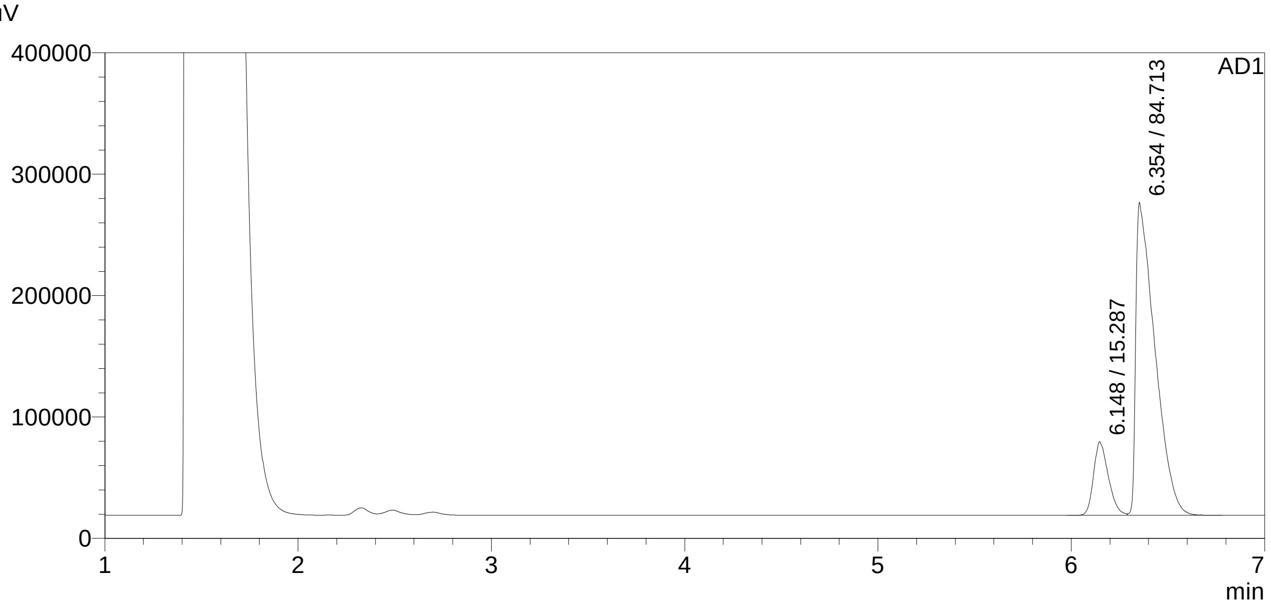

<Peak Table>

AD1

Peak#	Name	Area%	Ret. Time	Area	Height
1		15.287	6.148	319915	60507
2		84.713	6.354	1772820	257371
Total		100.000		2092735	317878

GC: Table 2, Entry 6

SHIMADZU LabSolutions

Analysis Report

<Sample Information>

Sample Name : clphpr
Sample ID : clphpr
Filename : YNC11602_01.dat
Method Filename : Yana1a.met
Batch Filename :
Vial # : 2
Injection Volume : 1 uL Level : 1
Date Acquired : 3/2/2016 11:14:36 AM
Date Processed : 11/1/2021 3:37:05 PM
Sample Type : ME

<Chromatogram>

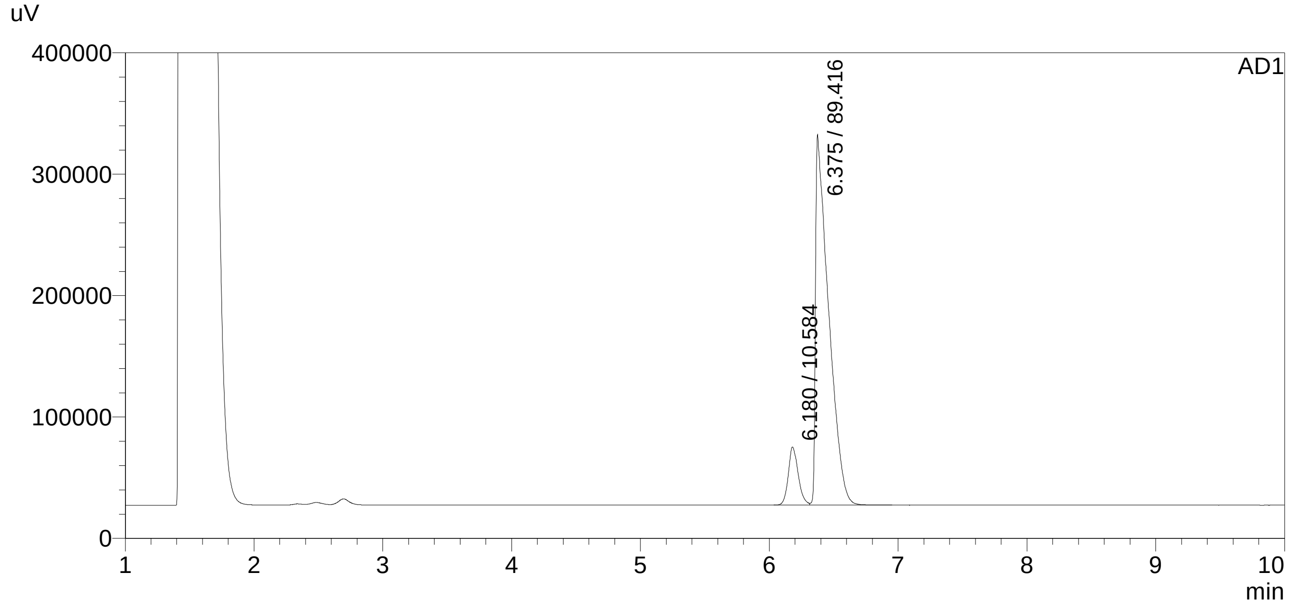

<Peak Table>

AD1

Peak#	Name	Area%	Ret. Time	Area	Height
1		10.584	6.180	256097	47861
2		89.416	6.375	2163605	304062
Total		100.000		2419702	351923
